# Mapping the absorption landscape of far-red Photosystem II

**DOI:** 10.1038/s41467-026-73964-7

**Published:** 2026-06-06

**Authors:** Ho Fong Leong, Giovanni Consoli, Geoffry A. Davis, Ben Hancox-Lachman, Kenta Renard, Fiazall Tufail, Lauren E. Lee, Lucas Gautier, James W. Murray, Andrea Fantuzzi, A. William Rutherford

**Affiliations:** 1 Department of Life Sciences, Imperial College, London, UK; 2 Present Address: Department of Plant Biochemistry, Biology, Ludwig Maximilian University of Munich; Planegg-Martinsried, Munich, Germany; 3 Present Address: Department of Chemistry, University of York, York, UK

**Keywords:** Bioenergetics, Cryoelectron microscopy, Photosystem II

## Abstract

Far-red light photoacclimation enables some cyanobacteria to survive in white-light-depleted environments by extending the red limit of photosynthesis. In far-red Photosystem II, paralogous subunits replace their canonical counterparts, allowing the incorporation of some chlorophyll *f* molecules and one chlorophyll *d* that are red-shifted and spectrally distinct from the chlorophyll *a* manifold, and from each other. Here, we present a comparative study of far-red Photosystem II from *Chroococcidiopsis thermalis* PCC 7203 and *Calothrix* sp. NIES-3974. In *C. thermalis*, the cryo-electron microscopy structure reveals the far-red-exclusive subunit, PsbH2’, which forms part of a chlorophyll *f* binding site. We also assign four chlorophyll *f* sites using sequence comparisons and electrostatic potential analyses. In *Calothrix, psbH2’* is absent, and the same analyses show that only two of these chlorophyll *f* sites are present. Comparative phylogenetic, structural, and spectroscopic analyses allow the assignment of specific wavelengths to all the red-shifted chlorophylls. This provides the framework needed to model excitation energy transfer in far-red Photosystem II, and to understand the conserved features that allow survival under far-red light.

## Abstract (web summary)

Some cyanobacteria do oxygenic photosynthesis with near-infrared photons using specialized pigments. Here, the authors identify their positions and colors by combining phylogenetic, spectroscopic, and structural methods.

## Introduction

Photosystem II (PSII) catalyzes a unique multi-electron redox reaction in the photosynthetic electron transport chain. Using energy from the sun, it abstracts electrons from water and reduces plastoquinones. As a by-product, it energizes the atmosphere through the release of molecular oxygen. Under increased ratios of far-red to white light, a diverse subset of cyanobacteria undergoes a process called far-red light photoacclimation (FaRLiP), in which all the light-driven components of the photosynthetic apparatus are dismantled and rebuilt with far-red light adapted paralogues, encoded in the FaRLiP cluster^1^. The far-red adapted photosystems incorporate a small number of longer wavelength pigments (i.e., chlorophyll *d* and chlorophyll *f*) in place of some chlorophyll (Chl) *a* molecules^1–4^.

Canonical “white light” PSII (WL-PSII) contains ~20 subunits and binds 35 Chl *a* molecules, most of which are used to harvest photons and direct excitation energy to the reaction center, where chlorophyll *a*-based photochemistry occurs^5^. In the FaRLiP cluster, four genes coding for far-red adapted paralogues of the main PSII subunits are present^1,6,7^: *psbA* and *psbD*, coding for the reaction center subunits (D1 and D2), *psbB* and *psbC*, coding for the two main antenna proteins of PSII (CP47 and CP43), and many FaRLiP clusters also contain the PSII subunit *psbH*. These subunits coordinate all of the Chl *f* molecules and the single Chl *d*^2^.

Most spectroscopic, biophysical, sequence, and structural data agree on the nature of the four chlorophylls present in the core of the reaction center of far-red-adapted PSII (FR-PSII): three Chl *a* molecules (P_D1_, P_D2_ and Chl_D2_) and a Chl *d* at the Chl_D1_ position which acts as the primary electron donor in charge separation^2,3,8^. Some experimental ambiguities in the spectroscopy^9^ and structure (i.e., inability to distinguish formyl groups from vinyls) have been discussed^9,10^, but the consensus has remained in favor of the original assignment that the primary donor is a Chl *d* at the Chl_D1_ position^2,3,8,10^.

In contrast, the assignments of the four antenna Chl *f* pigments in previously published FR-PSII structures were less well supported. The first structure determined, a monomeric FR-PSII from *Synechococcus* sp. PCC 7335^3^, lacked several extrinsic subunits, including PsbH2 (the far-red paralogue of PsbH). Additionally, some chlorophylls were absent, and some structural features differed from those of intact WL-PSII, which may have arisen from damage during purification. More recently, the structure of a dimeric FR-PSII from the same organism was reported^4^, showing all 35 expected chlorophyll molecules and partial occupancy of some of the extrinsic proteins. However, the improved resolution allowed only some of the Chl *f* molecules to be assigned by analysis of the electrostatic potential (ESP) around the C2 substituent, differentiating methyl groups (Chl *a*) from formyl groups (Chl *f*)^11^, while the others were inferred mainly by sequence comparisons^4^.

The low temperature absorption spectrum of FR-PSII in *Chroococcidiopsis thermalis* PCC 7203 (referred to as *C. thermalis* hereafter) shows five partially resolved absorption peaks red-shifted beyond the absorptions of the manifold of overlapping chlorin molecules (i.e., 30 Chl *a* and 2 Pheophytin *a*)^2^. These features originate from the long-wavelength chlorophylls and provide an unprecedented opportunity in oxygenic photosynthesis to identify the location and site energies of several antenna pigments. These assignments, and their relation to conserved structural changes, are essential to understand the routes of excitation energy transfer selected by evolution in far-red photosystems, and how these compare to those in their white-light counterparts.

To address these questions, we obtained a cryo-electron microscopy (cryoEM) map of a dimeric, intact FR-PSII complex from *C. thermalis* PCC 7203 at a Gold-Standard Fourier Shell Correlation (GS-FSC) resolution of 2.17 Å (Supplementary Fig. 1, Supplementary Table 1) (pdb_00009T5T) and located the four antenna Chl *f* molecules. The map shows ESP for the far-red-exclusive PSII subunit PsbH2’^7^, allowing the characterization of its location and interactions with PsbH2. Phylogenetic analyses of PsbH2’ are presented, along with hypotheses on its function and origin.

We also report a cryoEM map of a monomeric FR-PSII from *Calothrix* sp. NIES-3974 (referred to as *Calothrix* sp. hereafter) at a GS-FSC resolution of 2.33 Å (Supplementary Fig. 2, Supplementary Table 2) (pdb_00009T5U). Unlike *C. thermalis*, this species lacks coding sequences for the far-red light paralogue *psbH2* and the additional subunit *psbH2’* in its genome, and possesses amino acid differences in PsbB2 (the far-red paralogue of PsbB) relative to *C. thermalis*. The *Calothrix* sp. map does not present density for the extrinsic subunits PsbO, PsbV, PsbU (Supplementary Fig. 3, Supplementary Table 3), but both maps present ESP density for the manganese cluster (Supplementary Fig. 4). Combined structural, spectroscopic and pigment quantification (Supplementary Fig. 5) analyses indicate the absence of two Chl *f* molecules in *Calothrix* sp., enabling the long wavelength peak assignments of all the far-red chlorophylls by correlating sequence polymorphisms with low-temperature absorption and fluorescence spectra and the ESP maps of the two species.

## Results and discussion

### Structural identification of the far-red light exclusive subunit PsbH2’

PsbH2’ has been proposed as a putative far-red specific PSII subunit^7^, with no known white light counterpart. PsbH2’ exhibits differences across lineages, with fused PsbH2:PsbH2’ protein isoforms present in Leptolyngbyales and Elainellales^7^. In all other species possessing a putative PsbH2’, the *psbH2’* coding sequence is immediately downstream of *psbH2*, and *psbH2’* is never found in the absence of *psbH2*. We note that a PsbH2’ phylogeny congruent with the species tree^12–14^ is inferred only when accounting for variable evolutionary rates across lineages^15^ (Supplementary Fig. 6, Supplementary Table 4, Supplementary Table 5), despite potential overfitting (Supplementary Fig. 7), suggesting possible functional heterogeneity of PsbH2’ in different cyanobacteria. Although the origin of this subunit remains unresolved, we hypothesize its emergence from the C-terminus of an ancestral PsbH sequence (see Supplementary Note S2).

**Fig. 1:**
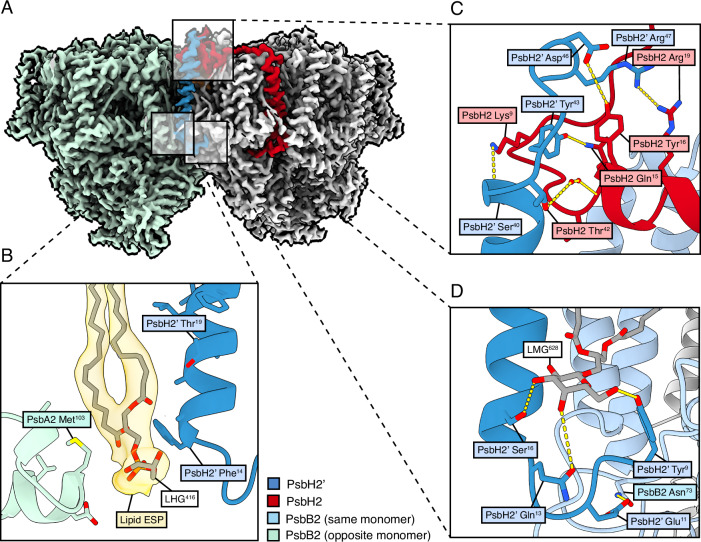
Structural description of the PsbH2’ subunit binding sites. **A** FR-PSII map from *C. thermalis*. The two monomers are shaded in gray and green, in the gray monomer PsbH2 is colored in red while PsbH2’ is in blue. **B** Interface between the two FR-PSII monomers, showing lack of protein-protein interaction between PsbH2’ and PsbA3 in the opposite monomer. The ESP density of the tentatively fitted lipid is represented in yellow. **C** Cytoplasmic interaction between PsbH2’ and PsbH2. **D** Cytoplasmic interaction between PsbH2’ with PsbB2 (in light blue) involving LMG^628^.

In the map of FR-PSII from *C. thermalis*, clear ESP for PsbH2’ is present (Fig. 1A). This single transmembrane helix is adjacent to PsbB2 and PsbH2. PsbH2’ is proximal to the dimeric interface of PSII but does not interact directly with any subunit of the opposite monomer (Fig. 1B). Matching conserved far-red specific residues are present throughout the sequences of PsbH2 and PsbH2’, suggesting the possible coevolution of the two proteins. The conserved residues seem to be specifically relevant to the interaction of PsbH2 with PsbH2’, in both the cytoplasmic (Fig. 1C) and lumenal (Fig. 1D) regions, and indicate that PsbH2’ can only bind to FR-PSII. On the lumenal side, the interaction of PsbH2 and PsbH2’ is also stabilized by the presence of a distearoyl-monogalactosyl-diglyceride (PsbB2 LMG^628^). In the high-resolution cryoEM map of WL-PSII from *T. vestitus* BP-1^16^, this lipid is characterized by poor ESP that does not allow its identification. By contrast, in this map the head group of PsbB2 LMG^628^ is defined by clear ESP due to the presence of conserved H-bonding residues in PsbH2’ that stabilize it (Fig. 1D).

### Chl B617 as a Chl *f* in *C. thermalis* PCC 7203

**Fig. 2:**
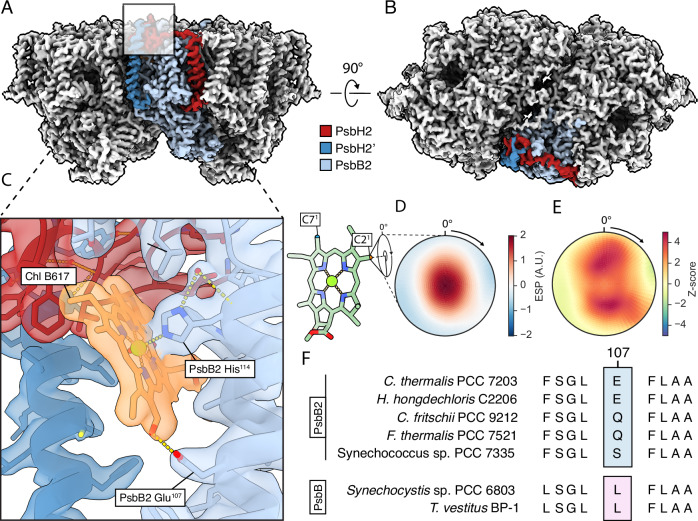
Structural evidence for Chl *f* in the B617 site. **A** CryoEM map of *C. thermalis* FR-PSII (white), highlighting PsbH2 (red), PsbH2’ (blue), and PsbB2 (light blue) from one of the monomers. **B** Position of PsbH2 and PsbH2’ in the FR-PSII map as seen from the cytoplasmic side. Colored as (**A**). **C** ESP map and atomic model of the Chl B617 site. The Chl *f* density is represented in orange, and the density of the amino acid residues from PsbB2, PsbH2 and PsbH2’ are colored as in (**A**). H-bonds and the coordinate bond are indicated as dotted lines. **D** ESP polar plot indicating the presence of an increased potential around the C2 substituent. The cone placement for the analysis of the C2 substituent is displayed on the left. **E** Z-score polar plot indicating the presence of an increased potential around the C2 substituent. **F** Alignment of FR and WL PsbB sequences around the amino acid that provides the B617 Chl *f* formyl H-bond.

Distinguishing the identity of chlorophyll molecules based solely on the ESP density of CryoEM maps has proven to be challenging at the typical resolutions obtained^3,4,11,17^. Therefore, to evaluate pigment identity in the maps presented here, other lines of evidence were used. Among these, statistically calibrated ESP scan analyses were used to assess whether the C2 substituent exhibits deviations from the methyl distribution expected for Chl *a* (Supplementary Fig. 8). This approach quantifies the electrostatic environment around each substituent, compares it to an internal methyl reference, and determines its statistical significance using dataset-specific Z-score thresholds derived from confusion matrix analysis (see Materials and Methods; Supplementary Note S3, Supplementary Fig. 9). This ESP analysis is interpreted in the context of the overall map quality and structural environment, and therefore complements, rather than replaces, visual inspection of the map. Of the previously assigned Chl *f* sites, three are consistent with the map of *C. thermalis* FR-PSII presented here (i.e., B608, B614, C507)^4^ (Supplementary Figs. 10, 11, 12). The other proposals for the fourth Chl *f* site from the *Synechococcus sp*. PCC 7335 structures were Chl B605 and B611^3,4,18^. In the present work, based on the updated ESP cone scan method (see Supplementary Note S3 and Materials and Methods), there is no evidence of increased ESP from the putative C2 formyl groups of these two chlorophylls. In contrast, here we assign Chl B617 as a Chl *f*, positioned at the interface between PsbB2, PsbH2, and PsbH2’ (Figs. 1, 2, Supplementary Fig. 13). To avoid confusion, the chlorophyll positional nomenclature is maintained from previous work on FR-PSII (Supplementary Fig. 15). This differs from other PSII chlorophyll nomenclatures, and so a nomenclature conversion table is presented in Supplementary Table 6.

Chl *f* B617 presents an increased ESP around the C2 substituent, and a clear protruding density attributed to the presence of a formyl oxygen (Fig. 2C–E). Moreover, the site has a far-red conserved change: a Leu (fully conserved in WL-PSII) is substituted with an amino acid capable of hydrogen bonding the Chl *f* formyl oxygen (Glu, Gln or Ser) at position 107 of PsbB2 (Fig. 2F, Supplementary Fig. 13). Supporting this conclusion, in the structure of FR-PSII from *Synechococcus* sp. PCC 7335^4^, the density of an unmodeled water molecule can be observed, which would bridge the formyl group of Chl *f* B617 and PsbB2 Ser^107^. Additionally, a second far-red conserved change is present in this site: PsbH Thr^5^ in WL-PSII, which hydrogen bonds the 13-keto group of Chl B617, is substituted in PsbH2 by the non-hydrogen-bonding Ala^2^.

Given the position of PsbH2’ within the complex and its proximity to Chl *f* B617, a possible function of PsbH2’ may be to stabilize Chl *f* specifically in the B617 site. Chl B617 is a peripheral chlorophyll that is membrane exposed in the absence of PsbH2’ and in WL-PSII. This site presents structural features that suggest inherently weaker binding for a Chl *f*. Chl *f* B617 is ligated by a histidine, which is considered to be a less favorable ligand compared to more polar residues or water molecules^19^. In addition, PsbH2 lacks Thr^5^ (present in PsbH) and its stabilizing H-bond to the 13^1^-keto group of this chlorophyll. In FR-PSII, the position of PsbH2’ reduces the exposure of Chl *f* B617 to the membrane. This may shield it from dissociation, compensating for its weaker coordinating environment. Supporting this, in *Synechococcus* sp. PCC 7335, where *psbH2’* is not present in the genome, the structure showed no evidence for Chl *f* B617, which was lost together with PsbH2 during isolation^3^. A similar situation is observed in far-red Photosystem I, where Chl *f* B30, a histidine-ligated peripheral chlorophyll, is absent in structures with partial or no occupancy of the stabilizing subunit PsaJ2^20,21^.

### Wavelength assignments of Chl *f* sites in FR-PSII

**Fig. 3:**
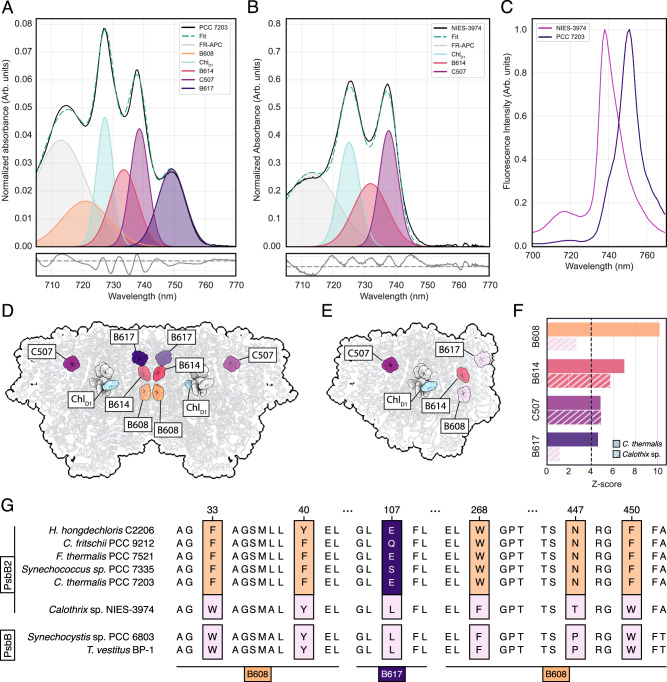
Wavelength and site assignments of far-red chlorophyll molecules in *C. thermalis* and *Calothrix* sp. **A** 77 K absorbance spectra and Gaussian deconvolution of FR-PSII from *C. thermalis*. Peaks are colored according to the in-figure legend. Fitting residuals are shown in the box below. **B** 77 K absorbance spectra and Gaussian deconvolution of FR-PSII from *Calothrix* sp. Peaks are colored according to the in-figure legend. Fitting residuals are shown in the box below. **C** 77 K fluorescence emission spectra of FR-PSII from *C. thermalis* and *Calothrix* sp. **D** Dimeric structure from *C. thermalis*, Chl *f* and *d* colored following assignment in (**A**). **E** Monomeric structure from *Calothrix* sp., Chl *f* and *d* colored following assignment in panel A. Pigments in pink are assigned as Chl *a*. **F** Maximum Z-score values in the C2 ESP substituent analysis (see materials and methods) with a cut-off of 4 standard deviations. **G** Multiple sequence alignment of PsbB and PsbB2. Conserved changes associated with Chl *f* sites are highlighted in the same color key as in the other panels. Source data are provided as a Source Data file.

The genetic variability of FaRLiP-capable strains and associated functional differences enable comparative analyses to determine relevant relationships between sequence and site energies. Notably, *Calothrix* sp. lacks both *psbH2* and *psbH2’* in its genome and is therefore likely to use white light PsbH in its FR-PSII. In addition, the *Calothrix* sp. PsbB2 lacks some of the specific amino acid changes that are conserved in most other far-red cyanobacteria. These conserved residues, which are missing in *Calothrix* sp., are important for Chl *f* binding in *C. thermalis* FR-PSII (Fig. 3G).

Therefore, FR-PSII from *Calothrix* was purified and characterized spectroscopically and structurally. The map, at a GS-FSC resolution of 2.33 Å, shows, as expected, the presence of PsbH in place of PsbH2 and no increased ESP around the C2 substituent of Chl B617 (Supplementary Figs. 13, 14), suggesting the presence of a Chl *a* instead of a Chl *f* in this site. Supporting this is the presence of a Leu instead of PsbB2 Glu^107^ which would hydrogen bond the C2 formyl oxygen if it were present (Fig. 3G).

In addition, several lines of evidence indicate that FR-PSII from *Calothrix* sp. lacks a second Chl *f* site. Chl B608 does not present any increased ESP around the C2 substituent, or any of the far-red conserved changes associated with the presence of a Chl *f* in this site (Supplementary Figs. 10, 14). In PsbB2 from *Calothrix* sp., the residues Phe^268^ and Thr^447^ are present in place of Trp^268^ and Asn^447^ found in most other far-red species. Their absence leads to the loss of the hydrogen-bonding water molecules that would stabilize a Chl *f* at this site. The presence of Chl *f* in the other two sites (i.e., B614, and C507) is confirmed by the analysis of the ESP around the C2 substituent of these chlorophylls (Supplementary Fig. 14), and by their associated far-red specific sequence changes (Supplementary Figs. 11, 12). The difference in the quantity of Chl *f* was confirmed by pigment quantification using HPLC (Supplementary Fig. 5).

This provides a unique opportunity to assign site energies to each respective long-wavelength pigment by comparing the low-temperature spectra of FR-PSII from both species. The two low-temperature absorption spectra show several different features. First, the absorption feature at 749 nm present in *C. thermalis* is absent in *Calothrix* sp. Second, the ratio between the two absorption peaks at ~725 nm and ~738 nm is different between the two organisms. The absence of two Chl *f* molecules (B608 and B617) in *Calothrix* sp. therefore corresponds to the loss of two absorptions in the low-temperature absorption spectrum, namely at 721 nm and 749 nm (Fig. 3A, B).

Previous spectroscopic data from *Synechococcus* sp. PCC 7335, a strain in which *psbH2’* is absent, showed that its FR-PSII has a single emission peak at ~740 nm at 77 K^3,22^. In comparison *C. thermalis* FR-PSII has two fluorescence emission peaks at ~740 nm and ~751 nm (Fig. 3C)^2,9,23^. Among all of the Chl *f* sites in FR-PSII from the two organisms, the only one that presents structural differences is Chl *f* B617. Specifically, this Chl is membrane exposed in *Synechococcus* sp. PCC 7335 and appears to be hydrogen bonded to a water coordinated by Ser^107^, while in *C. thermalis* Chl *f* B617 is protected by PsbH2’ and hydrogen bonded by Glu^107^ (Fig. 2). Similarly, the FR-PSII low-temperature fluorescence emission spectrum of *Calothrix* sp. reveals comparable changes in its terminal emitter (Fig. 3C), with a single emission peak at ~740 nm (Fig. 3C). The loss of the 750 nm low-temperature emission in both the *Synechococcus* sp. PCC 7335 and *Calothrix* sp. strongly suggests that Chl *f* B617 is responsible for the observed 750 nm fluorescence in *C. thermalis*, allowing Chl *f* B617 to be assigned as the 749 nm absorbing pigment. It follows that Chl *f* B608, the other Chl *f* site missing in *Calothrix* sp., corresponds to the 721 nm absorbing pigment. This conclusion holds irrespective of whether B617 is considered to be a Chl *a* in *Synechococcus* PCC 7335, as previously reported^3,4^, or to be a Chl *f* as suggested by the structural observations presented here. Since the Chl *d* located at the Chl_D1_ site (Supplementary Fig. 16) was previously shown to absorb at 727 nm based on light-minus-dark difference spectra^2^, the two pigments in common between *C. thermalis* and *Calothrix* sp. (B614 and C507) are the only candidates left for the 734 nm and 737 nm absorption bands.

The presence of two distinct fluorescence emission peaks at 77 K in the FR-PSII of *C. thermalis* suggests that the corresponding terminal emitters are excitonically disconnected and therefore likely located in separate antenna subunits^2,23^. By excluding pigments near Chl *f* B617 (specifically Chl *f* B608 and Chl *f* B614), the ~740 nm emission peak can be attributed to Chl *f* C507, likely absorbing ~737 nm. This leaves Chl *f* B614 responsible for the broader 734 nm low-temperature absorption^2^. Additional evidence also supports the assignment of Chl *f* B614 as the 734 nm pigment. Chl *f* B614 is the closest pigment to Q_A_, and the only one that has its transition dipole moment oriented so that it would give rise to the previously reported electrochromic redshift centered around 734 nm in the presence of Q_A_^·^^−^^2^.

### Towards understanding the absorption landscape of FR-PSII

**Fig. 4:**
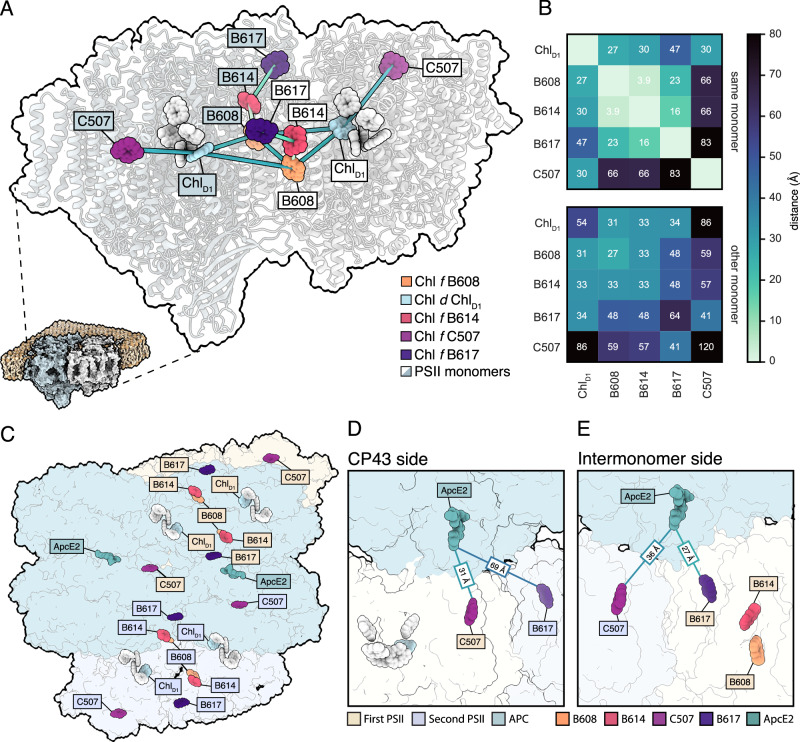
Long-wavelength pigment organization in FR-PSII and FR-APC - FR-PSII supercomplexes. **A** Overview of the FR-PSII complex viewed from a tilted cytoplasmic/side view, highlighting the spatial distribution of long-wavelength pigments. The only Chl *a* molecules shown are in the reaction center (gray). Long wavelength pigments are colored according to the legend in (**A**). Distances between pigments are represented using the color scale shown in (**B**). **B** Edge-to-edge distance matrix of long-wavelength pigment sites within a FR-PSII dimer. **C** Cytosolic view of two adjacent FR-PSII dimers (yellow and lilac) connected to a bicylindrical FR-APC (blue). The FR-PSII model (pdb_00009T5T) and the FR-APC model (pdb_00009I1R) were aligned to the structure of the supercomplex from *A. platensis* (pdb_00008WQL). **D** Close-ups of the FR-APC–FR-PSII interface viewed parallel to the membrane plane on the side where the ApcE2 subunit is positioned above CP43. Chl *f* molecules and labels colored according to the legend below. **E** Close-ups of the FR-APC–FR-PSII interface viewed parallel to the membrane plane on the side where the ApcE2 subunit is located between the adjacent FR-PSII dimers. Chl *f* molecules and labels colored according to the legend below.

FR-PSII has yields of charge separation comparable to those of WL-PSII despite having a reduced number of pigments (i.e., the antenna Chl *f* molecules) involved in excitation energy transfer to the reaction center^24^. In the photon-limited environments in which FaRLiP evolved, there was likely an evolutionary pressure for antenna Chl *f* molecules to be strategically placed and tuned to increase the rates of excitation energy transfer not only to the Chl *d* primary donor in the same monomer, but also to the antenna Chl *f* sites in the adjacent monomer to maximize the number of productive charge separation events for available photons (Fig. 4).

In the dimeric assembly of PSII, CP43 is in a distal position with respect to the dimer interface, while CP47 is one of the main components of the dimerization interface. The asymmetry in the long wavelength pigments present in CP43 and CP47 (a 1:3 Chl *f* ratio), suggests an advantage for Chl *f*-containing photosystems to share long wavelength pigments over multiple monomers (Fig. 4). As noted in FR-PSI trimers, the location of most of the long wavelength pigments in the core of the multimeric assembly might facilitate excitation energy transfer between monomers, ensuring efficient energy redistribution when a reaction center is closed or damaged^21^. Chl *f* B608 and B614 seem key to the excitation energy transfer landscape of FR-PSII in *C. thermalis*, being not only the closest Chl *f* molecules to Chl_D1_ in the same monomer, but also the closest to the primary donor of the adjacent monomer, allowing them to act as an excitation energy bridge between the reaction centers (Fig. 4B). The loss of the B608 as a Chl *f* in *Calothrix* sp., however, highlights the diversity in far-red light absorption and excitation transfer strategies between different Chl *f* producing species.

### Connection to the FR-APC

The structural identification of PsbH2’ might be relevant to the connection of far-red allophycocyanin (FR-APC) to FR-PSII. To evaluate this possibility, the models of *C. thermalis* FR-APC^25^ and FR-PSII from the present work were rigid-body-fitted to the in situ structure of the phycobilisome-photosystem II supercomplex from *Arthrospira platensis*^26^ (Fig. 4). In the FR-PSII-FR-APC arrays, ApcE2 and ApcD3 are located between adjacent FR-PSII dimers close to PsbH2 and PsbH2’ (Supplementary Fig. 17), with several polar residues on both complexes available for hydrogen bonding with one another. This suggests that PsbH2’ may also play a role in stabilizing the interaction between FR-PSII and FR-APC, and/or that it prevents the association of white light phycobilisomes to FR-PSII through steric hindrance.

Based on this structural modeling, Chl *f* C507 and Chl *f* B617 are the two closest pigments to the cytoplasmic surface of FR-PSII and to the terminal emitters of FR-APC (ApcE2) in *C. thermalis*, making them the best candidates for bridging excitation energy transfer between the two complexes. The distances of the FR-APC terminal emitters to Chl *f* molecules in this complex reveal two different pictures when comparing the side in which ApcE2 is positioned on top of CP43 (Fig. 4D), and the one in which ApcE2 is located between the two adjacent FR-PSII dimers (Fig. 4E). On the CP43 side, the closest Chl *f* pigment is C507, at ~31 Å (Fig. 4D). Conversely, on the other side, the closest long-wavelength pigment is Chl *f* B617 at ~27 Å, with Chl *f* C507 at ~36 Å (Fig. 3D). One limitation of the modeling approach is that the overall phycobilisome - PSII architecture derived from a non-FaRLiP strain was used to position the FR-APC and FR-PSII components. Consequently, potential far-red specific structural rearrangements in the supercomplex may not be captured in the current model.

Previous calculations, based on an earlier structural model^3^, together with fast fluorescence emission data^23^, identified Chl *f* C507 as the sole viable excitation injection point in FR-PSII from the FR-APC, due to its proximity to the cytoplasmic surface of FR-PSII and its calculated rapid energy transfer to Chl_D1_^23^. This is consistent with the site energy assignments presented here, where the absorption spectrum of Chl *f* C507 overlaps with the fluorescence emission spectrum of FR-APC. The fast fluorescence data^23^, taken together with the present results, indicate that Chl *f* C507 remains the most plausible excitation injection point from the FR-APC, with Chl *f* B617 possibly acting as an energy dissipation point. It should be noted that while the fast fluorescence measurements were performed with far-red adapted *C. thermalis*^23^, the data were originally interpreted using the Chl *f* site assignments from *Synechococcus* sp. PCC 7335 FR-PSII^4^, which did not propose B617 as a Chl *f* site.

### Broader implications

Due to spectral overlap, chlorophyll wavelength assignments have been limited for WL-PSII. Here, the diversity of FaRLiP has allowed the assignment of both the locations and site energies of the long wavelength chlorophylls in FR-PSII based on the integration of genetic, structural, and spectroscopic data. In light of the updated Chl *f* site assignments, computational modeling of the excitation energy transfer pathways within FR-PSII, from the FR-APC^25^ to FR-PSII, as well as in ordered arrays of FR-PSII - FR-APC supercomplexes, should now be achievable.

A particularly interesting aspect of the current assignments is the Chl *f* in position B617. This site is one of the main candidates for the “red-most” chlorophyll in WL-PSII and has been suggested to be part of an excitation energy dissipation pathway^27^. The finding that this chlorophyll is maintained in FR-PSII as the lowest energy pigment suggests a universal role in excitation dissipation through this chlorophyll across the phylogenetic diversity of PSII.

The impact of having fewer long-wavelength pigments in *Calothrix* sp. remains unclear, but could reflect changes in the selective pressure for FaRLiP, or an adaptation to a different environmental niche. FR-PSII in *Calothrix* sp. is not, however, an isolated case where Chl *f* sites differ in far-red adapted photosystems, as variations in 77 K absorbance of FR-PSI from different species have also been shown^28^. In the extreme, significant loss of FR-PSI genes within the FaRLiP cluster has been observed in *Chroococcidiopsis* sp. CCMEE 010, and complete loss of FR-PSII and FR-APC genes in the FaRLiP cluster of *Nostoc* sp. C052^29^.

The existence of FaRLiP on a genetic and functional spectrum presents a resource to pinpoint divergent and conserved features that are necessary for survival in far-red light conditions, as well as contributing to our understanding of canonical WL-PSII.

## Methods

### Culture conditions

*Chroococcidiopsis thermalis* PCC 7203 and *Calothrix* sp. NIES-3974 were grown in liquid BG11 medium^30^ at 30 °C under 750 nm LED illumination at an intensity of ~30 μmol photons m^–2^s^−1^ (750 nm, Epitex; L750-01AU). Cultures were harvested, pelleted and flash frozen after at least 3 months of exposure to exclusively far-red illumination.

### Isolation of FR-PSII complexes

All steps were performed in darkness or in dim green light. 750 nm far-red grown cells were broken with two passages in a flow cell disruptor (Constant Systems) at a pressure of 39 kPsi and then centrifuged at 1000 × *g* to remove any unbroken material. The supernatant was centrifuged at 186,000 × *g* in a Ti45 rotor at 4 °C for 20 min to pellet the membranes. Membranes were resuspended in low salt buffer A (20 mM MES, pH 6.5, 5 mM MgCl_2_, 5 mM CaCl_2_, 1.2 M betaine, 0.03% β-DM), adjusted to a chlorophyll concentration of 0.4 mg ml^−1^ and solubilized at 4 °C for 1 h by the addition of β-DM to a final concentration of 0.4% (w/v). Non-solubilized material was removed by centrifugation at 205,100 × *g* at 4 °C in a Ti45 rotor for 30 min. The supernatant was then loaded onto a DEAE Toyopearl 650S column (inner diameter: 50 mm, length: 450 mm, 500 mL column volume, Tosoh Bioscience GmBH, Griesheim, Germany) and washed with 1 column volume of buffer A. Protein complexes were eluted with a 0%-100% linear gradient of buffer B (20 mM MES, pH 6.5, 5 mM MgCl_2_, 5 mM CaCl_2_, 0.5 M NaCl, 1.2 M betaine, 0.03% β-DM) over 5 column volumes. Chlorophyll and bilin containing fractions were collected, concentrated (100 kDa Filters, Amicon) and stored at 4 °C or −80 °C depending on the use. Samples were loaded for a further purification step on a DEAE Toyopearl 450S (inner diameter: 25 mm Length: 450 mm, 150 mL column volume, Tosoh Bioscience GmBH) and washed with 1 column volume of buffer A. Protein complexes were eluted with a 0–100% linear gradient of buffer B over 5 column volumes. Chlorophyll and bilin containing fractions were collected, concentrated (100 kDa Filters, Amicon) and stored at 4 °C or −80 °C depending on the use.

### 77 K absorption measurements

77 K absorption measurements were made with a Cary 6000 Series UV-Vis-NIR Spectrophotometer (Agilent Technologies, Santa Clara, CA, USA) using a OptistatDN cryostat (Oxford Instruments, High Wycombe, UK). The cuvette comprised two quartz high precision cell slides (Hellma Analytics GmbH, Müllheim, Germany) separated by a 1 mm polytetrafluoroethylene spacer with a central hole aligned to the spectrophotometer beam, all housed within a custom-built gasket. A total volume of 40 µL, comprising 24 µL of 100% glycerol and 16 µL of sample at approximately 1 mg mL^−1^ chlorophyll, was suspended under dim green light between the two sides with a resulting path length of 1 mm. The sample was cooled by one degree per minute in darkness until it reached 77 K, and spectra were recorded from 1000 to 600 nm. A separate blank measurement in which glycerol was mixed with buffer A was taken to allow for baseline subtraction. The 77 K absorption spectrum from *C. thermalis* was taken from Nürnberg et al.^2^.

### 77 K fluorescence spectroscopy of PSII complexes

1 mL samples of isolated FR-PSII complexes at an OD_680_ of 0.1 were frozen in liquid nitrogen in darkness and fluorescence measured with a FluoroMax 4 spectrofluorometer (HORIBA Ltd., Japan) while suspended in liquid nitrogen to maintain the temperature. Fluorescence emission was measured from 650 nm to 850 nm with an excitation light of 440 nm, 2 nm emission slit and 0.5 s integration time.

### Grid preparation

Gold quantifoil R 2/1 300 mesh grids were glow-discharged for 30 s at 25 mA, and 3.5 μL of PSII sample (~1 mg mL^−1^ of Chl concentration) was applied in the presence of dim green light. Grids were blotted for 3 s at 4  °C and 100% humidity and plunge-frozen in liquid ethane with a Vitrobot Mark IV (Thermo Fisher Scientific).

### CryoEM data collection

For the ESP map associated with *C. thermalis* (pdb_00009T5T), micrographs were acquired using a Krios III (Thermo Fisher Scientific) operated at 300 kV and a magnification of 155,000x. Images were recorded on a Falcon 4i (Thermo Fisher Scientific) with a pixel size of 0.723 Å and a dose of 40 electrons per Å^2^ for a total of 40 frames. Images were collected in super resolution mode with a SelectrisX energy filter with a slit width of 20 eV. The targeted defocus range was varied from –0.8 to –2 µm using the EPU software (Thermo Fisher). A total of 23,311 movies were collected from a single grid. The frames were aligned, dose weighted, and the contrast transfer function (CTF) was estimated in CryoSPARC v4.4.1^31^. Micrographs were curated by removing those with CTF fits worse than 10 Å. The subset contained 93% of the initial micrographs and was used to hand pick ~1000 PSII-type particles across the defocus range. Particles were 2D classified and used to template pick across the entire dataset, yielding 1,087,739 picks. After multiple rounds of 2D classification and ab initio refinement, duplicated particles were removed from the stack, and ~20,000 particles were used for homogeneous refinement to obtain an initial map at a resolution of ~3 Å, which confirmed C2 symmetry. After multiple rounds of per particle CTF refinement and local motion correction, a complete set of 49,763 particles was used to perform non-uniform refinement imposing C2 symmetry, producing a map at a global resolution of 2.17 Å at the GS-FSC cut-off of 0.143. The Guinier analysis reports an estimated B-factor of 39.7.

For the ESP map associated with *Calothrix* sp. (pdb_00009T5U), micrographs were acquired using a Krios G3i (Thermo Fisher Scientific) operated at 300 kV and a magnification of 105,000x. Images were recorded on a K3 Bioquantum (Ametek-Gatan) with a pixel size of 0.827 Å and a dose of 60 electrons per Å^2^. Images were collected in super resolution mode with a SelectrisX energy filter with a slit width of 20 eV. The targeted defocus range was varied from –0.4 to –1.8 µm using the EPU software (Thermo Fisher). A total of 17,792 movies were collected from a single grid. The frames were aligned, dose weighted, and the CTF was estimated in CryoSPARC v4.4.1^31^. Micrographs were curated by removing those with CTF fits worse than 10 Å. The subset was used to hand pick ~1000 PSII-type particles across the defocus range. Particles were 2D classified and used to template pick across the entire dataset. After multiple rounds of 2D classification and ab initio refinement, duplicated particles were removed from the stack, and ~20,000 particles were used for homogeneous refinement to obtain an initial map at a resolution of ~3 Å. After multiple rounds of per particle CTF refinement and local motion correction, a complete set of 285,844 particles was used to perform non-uniform refinement, producing a map at a global resolution of 2.33 Å at the GS-FSC cut-off of 0.143. The Guinier analysis reports an estimated B-factor of 63.7.

### Model building

The *Synechococcus* sp. PCC 7335 FR-PSII dimeric model (pdb_00008EQM)^32^ was fitted to the ESP map associated with *C. thermalis* (pdb_00009T5T) using the Phenix software suite^33^, mutated to the correct sequence with Chainsaw (Phenix) and refined in Coot^34^. The density of each subunit was evaluated amino acid by amino acid, and the model was then refined in real space in Phenix. The ESP map associated with *Calothrix* sp. (pdb_00009T5U) was fitted with a monomer from the *C. thermalis* FR-PSII model (pdb_00009T5T) and then processed in the same way as pdb_00009T5T.

### De novo sequence identification of PsbH2’

The density of the unknown subunit present in the *C. thermalis* ESP map was built with Modelangelo^35^, and at the same time fitted with a polyalanine helix of the correct length. Amino acids retrieved with Modelangelo that presented a confident density in the map were mutated on the polyalanine helix. The resulting sequence was then blasted against the genome of *C. thermalis* PCC 7203 and corresponded to PsbH2’. The sequence was adjusted accordingly and the model refined in Phenix.

### Quantification and statistical analysis of the electrostatic potential

The ESP is interpolated on the surface of a cone with its axis extending from that of the CX – CX’ bond, where X is the IUPAC number given to the chlorin carbons that have relevant substitutions, and with an aperture of 120° ($2\theta$). The ESP is sampled within the range of distances from 0 Å to 2.5 Å ($d$), with a step size of 0.1 Å. This is repeated for every 5° rotation of the torsion angle ($\varphi$). The 90° torsion angle is defined as being parallel to the plane defined by the positions of the three carbon atoms, CX-1, CX and CX’, in the direction of CX-1. The direction of rotation of the torsion angle is clockwise by IUPAC convention.

The implementation is written in Python using the GEMMI library^36^. The average (μ) and standard deviation (σ) of the ESP on the surface of the cone are computed for each of the substituents (C2, C3, C7, C8, C12). It thus provides visual information on the quality of the map and on the preferential orientation of the vinyl (C3) and ethyl (C8) substituents with respect to the chlorophyll plane. It also calculates the precision, recall and accuracy as metrics of the quality of the analysis. To classify the substituents in the C2 position, the Z-score of each sampling point is computed with respect to the average methyl substituent in position C7.

$${Z}_{exp }(d,\varphi )=\frac{{{ESP}}_{exp }^{C2}(d,\varphi )-{\mu }_{exp }^{C7}(d,\varphi )}{{\sigma }_{exp }^{C7}(d,\varphi )}$$

The ESP and the Z-scores on the surface of the cones are plotted as 2D projections looking down the CX – CX’ axis. This approach allows for the possibility that maximum differences between substituents could occur outside of the predicted length of the formyl carbon–oxygen double bond. It thus explores a larger area compared with the original cone-scan method which sampled a single distance and gave a one-dimensional reading around the cone^11^. Moreover, using Z-scores instead of the strict $\mu+3\sigma$ cutoff as used previously^11,17^ allows a more quantitative investigation of both positive and negative features in the environment of the substituent.

The calculations presented are obtained using a PDB in which every chlorophyll molecule was substituted with a Chl *a* and then refined in the unsharpened map as described in the methods section to prevent rotations in the Chl ring that could bias the results of the scan^37^.

To select an appropriate Z-score threshold for classifying substituents as significantly different from a methyl distribution, confusion matrices were computed across a range of Z-score cutoffs (0–5). C3 and C8 were used as positive references (non-methyl substituents), and C7 and C12 as negative references (methyl substituents). True positives (TP) were defined as non-methyl substituents outside the methyl distribution, false positives (FP) as methyl substituents outside the methyl distribution, true negatives (TN) as methyl substituents within the methyl distribution, and false negatives (FN) as non-methyl substituents within the methyl distribution. Precision, recall, and accuracy were calculated for each cutoff:

$${{{\rm{Precision}}}}=\frac{{TP}}{{TP}+{FP}},\,{{{\rm{Recall}}}}=\frac{{TP}}{{TP}+{FN}},\,{{{\rm{Accuracy}}}}=\frac{{TP}+{TN}}{{TP}+{TN}+{FP}+{FN}}$$

The Z-score threshold maximizing accuracy was selected as the optimal cutoff, providing a dataset-specific criterion for identifying substituents with statistically significant deviations in ESP.

### Sequence retrieval and alignment

PsbH2’ sequences were retrieved using BLASTP^38^ from the NCBI non-redundant database on 16/10/24 and 13/11/24 using *Gloeobacter kilaueensis* JS1 PsbH and *Chroococcidiopsis thermalis* PCC 7203 PsbH2’ sequences as queries. A word size of 2 was used. PsbH2’ and PsbH2 sequences were aligned using the MAFFT G-INS-i (v7.520) algorithm^39^ and manual readjustments were made to unalign PsbH2’ regions aligned to PsbH2 regions. Genetically non-fused PsbH2’ sequences were appended to PsbH2 sequences of the same species in AliView^40^. Regions homologous to the PsbH2’ region and C-terminus of the PsbH2 region were taken and re-aligned using the MAFFT E-INS-i (v7.520) algorithm^39^. Residues not aligned to unfused PsbH2’ isoforms were removed. C-termini of *Gloeobacter* PsbH sequences were added to the dataset using the MAFFT (version 7) E-INS-i algorithm^39^.

### Phylogenetic analyses and model evaluation

Phylogenetic analyses and model testing were performed using IQ-TREE2 (v2.3.6)^41,42^. All starting trees were set to BIONJ. The -nstop value was set to 300. Branch supports correspond to Shimodaira–Hasegawa-like approximate likelihood ratio test values^43^. Root testing was performed by re-rooting the unrooted tree at every position and evaluating each rooted tree under the N.Q.Pfam+H3 model^44^. For each rooted tree, log-likelihoods were evaluated, and Approximately Unbiased tests were performed^45^.

### Modeling of FR-APC-FR-PSII supercomplex

The structural models of FR-PSII (pdb_00009T5T) and FR-APC (pdb_00009I1R)^25^ from *C. thermalis* were aligned to the phycobilisomes-PSII supercomplex from *Arthrospira platensis* (pdb_00008WQL)^26^. using the matchmaker function in ChimeraX 1.9^46^.

### Pigment composition analysis via high performance liquid chromatography

5 μL of FR-PSII sample was added to 400 μl of methanol to extract pigments from the proteins. Samples were mixed via pipetting and centrifuged at 16,000 × *g* for 10 min to remove large insoluble fractions. The samples were then passed through a 0.22 μm PTFE filter and subjected to high performance liquid chromatography (Infinity II HPLC, Agilent Technologies) using an Infinity Lab Poroshell 120 EC-C18 2.7 μm 3 × 150 mm column (Agilent Technologies) kept at 35 °C and pre-equilibrated with 42% methanol, 33% acetonitrile, and 25% water (Mixture A).

5 μL of pigment sample was injected into the column at a flow rate of 0.8 mL min^−1^. The column was washed for 10 min with Mixture A, followed by 5 min of Mixture B (48% methanol, 50% acetonitrile, 2% water), 10 min of Mixture C (88% methanol, 10% ethyl acetate, 2% water), then 10 min of Mixture D (48% methanol, 50% ethyl acetate, 2% water). The column was then returned to its initial state by washing with 10 min of Mixture B followed by 10 min of Mixture A. Pigment elution was detected via diode array readings at 665 nm, 696 nm, and 707 nm, corresponding to the absorption peaks for chlorophyll *a*, chlorophyll *d*, and chlorophyll *f*, respectively. Identities of the elution peaks were confirmed via the absorption spectra of each peak. Pigment quantification was performed by calculating the peak area ratio of each chlorophyll. Peak area integration was done automatically using the Agilent OpenLab CDS software for each respective absorption maximum of the pigments. The peak area was then scaled according to their extinction coefficients^47^ as a proxy estimate of molar quantities.

### Constrained Gaussian deconvolution of FR-PSII low-temperature absorption spectra

Low-temperature absorption spectra of isolated FR-PSII complexes from *Calothrix* sp. NIES-3974 and *C. thermali*s PCC 7203 were analyzed to resolve the far-red absorbing chlorophyll contributions. Prior to analysis, the spectra were normalized to the maximum of the chlorophyll a Q_y_ band at ~680 nm to ensure comparable chlorophyll content between samples. The spectra were then restricted to the 700–770 nm region, and baseline correction was performed by subtracting the intensity at 770 nm from all data points. Spectral decomposition was performed by fitting each dataset to a sum of Gaussian functions. The two spectra were fit simultaneously using a shared parameter vector. The model consisted of: (i) A Gaussian component centered at 713 nm, corresponding to residual FR-APC contaminant. This component was included in both spectra with independent amplitudes but a shared width parameter. No area constraint was imposed on this peak, reflecting variable FR-APC content between preparations. (ii) Three chlorophyll Gaussian components representing far-red chlorophyll pigments in *Calothrix* sp. FR-PSII. (iii) Five chlorophyll Gaussian components representing far-red chlorophyll pigments in *C. thermalis* FR-PSII. Each chlorophyll-associated Gaussian had independently optimized center wavelength and width parameters. However, the integrated areas of all chlorophyll-specific Gaussians were constrained to be equal. This constraint reflects the expectation that the Q_y_ transitions of individual chlorophyll molecules possess similar oscillator strengths and that differences in spectral shape arise primarily from site-dependent energy shifts rather than differences in absorption strength. Imposing equal integrated areas also reduces parameter degeneracy inherent to multi-Gaussian decompositions of partially overlapping bands. The FR-APC 713 nm component was excluded from this equal-area constraint, as its intensity depends on variable levels of co-purified pigment. Nonlinear least-squares optimization was performed using Sequential Least Squares Programming (SLSQP) from SciPy to minimize the combined residual sum of squares across both spectra. All amplitudes were constrained to be non-negative, widths were constrained to positive values, and center wavelengths were restricted to physically reasonable bounds within the fitted spectral window. Optimization was performed until convergence (maximum 10,000 iterations; function tolerance 10^−9^). Goodness of fit was assessed using the coefficient of determination (R²: *C. thermalis* PCC 7203 = 0.997, *Calothrix* sp. NIES-3974 = 0.997), and the root mean square error (RMSE). The residuals display no systematic structure across the fitted wavelength range.

### Reporting summary

Further information on research design is available in the Nature Portfolio Reporting Summary linked to this article.

## Supplementary information

Supplementary Information

Reporting Summary

Transparent Peer Review file

## Source data

Source Data

## Data Availability

The atomic coordinates of the PDB models generated in this study have been deposited in the PDB under accession codes pdb_00009T5T [10.2210/pdb9T5T/pdb] (*C. thermalis* PCC 7203) and pdb_00009T5U [10.2210/pdb9T5U/pdb] (*Calothrix* sp. NIES-3974). The cryoEM volumes have been deposited in the EMDB under accession codes EMD-55593 (*C. thermalis* PCC 7203) and EMD-55594 (*Calothrix* sp. NIES-3974). Phylogenetic data has been deposited in Figshare as entry 30789941. Source data are provided with this paper.

## Code availability

Data and scripts used in data processing, analysis, and visualization are available as Zenodo repository entry 19827093.
