# Supplementary material for: Mapping the absorption landscape of far-red Photosystem II
Source: Nat Commun. 2026 Jun 6;17:7223. doi: 10.1038/s41467-026-73964-7 (PMC13396731; doi:10.1038/s41467-026-73964-7)
Supplement: Supplementary file 1 — Supplementary Information [file 41467_2026_73964_MOESM1_ESM.pdf]

Supplementary Materials for

Mapping the absorption landscape of Far-Red Photosystem II

Authors: Ho Fong Leong[1]†, Giovanni Consoli[1]†, Geoffry A. Davis[1]†‡, Ben Hancox-Lachman[1], Kenta Renard[1], Fiazall Tufail[1]§, Lauren E. Lee[1], Lucas Gautier[1], James W. Murray[1], Andrea Fantuzzi[1]*, A. William Rutherford[1]*

Corresponding authors: a.rutherford@imperial.ac.uk, a.fantuzzi@imperial.ac.uk.

The PDF file includes:

figs. S1 to S17
Supplementary notes S1 to S3
tables S1 to S6

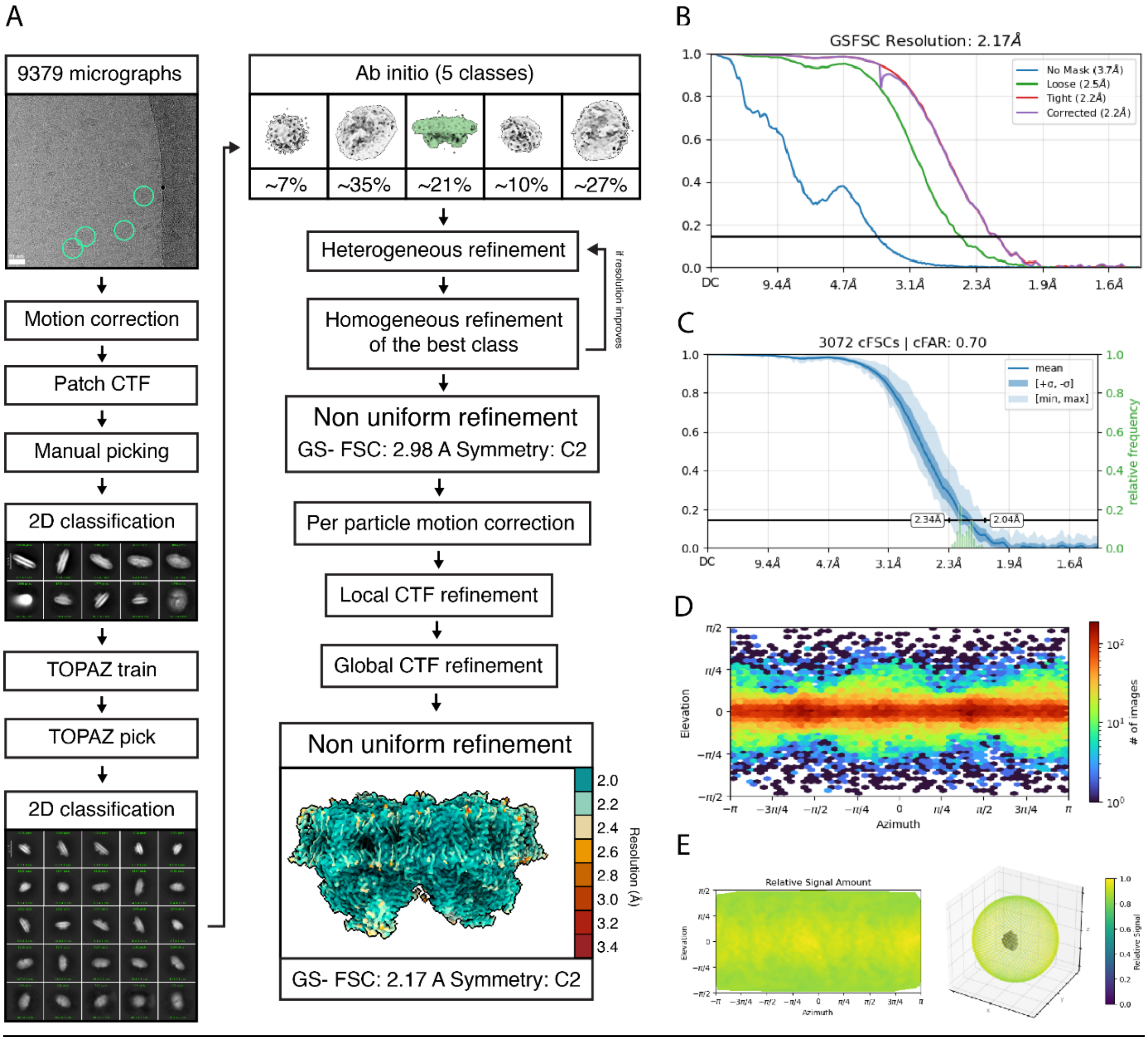

fig. S1 – CryoSPARC processing workflow for *C. thermalis* PCC 7203 FR-PSII pdb_00009T5T

A) Processing workflow to obtain the final reconstruction of the map associated with pdb_00009T5T. B) GS-FSC curve of the map associated with pdb_00009T5T. C) cFSC curves of the map associated with pdb_00009T5T. D) Viewing direction distribution of the particle stack. E) Relative signal amount for each of the viewing directions.

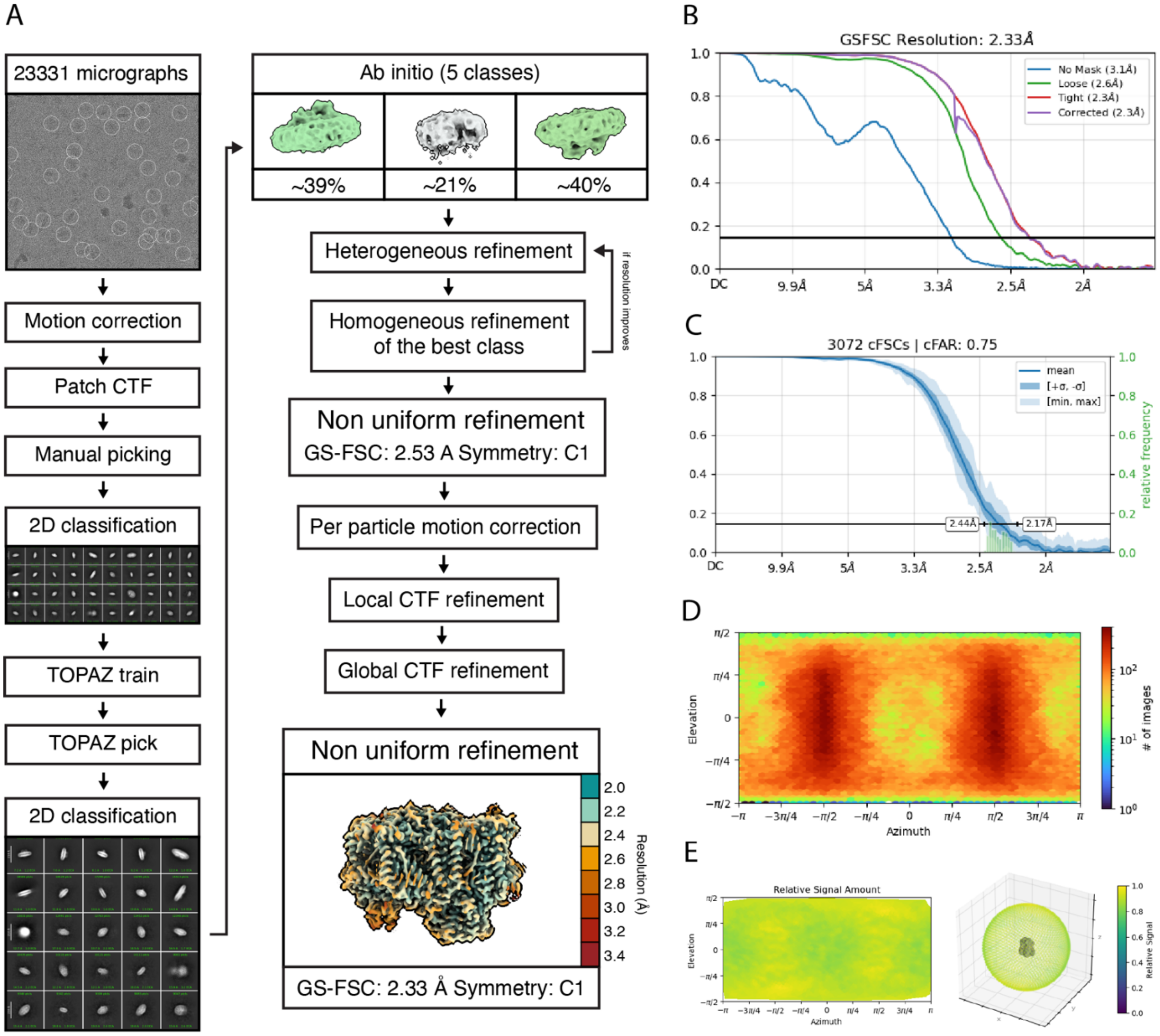

fig. S2 – CryoSPARC processing workflow for *Calothrix* sp. NIES-3974 FR-PSII pdb_00009T5U

A) Processing workflow to obtain the final reconstruction of the map associated with pdb_00009T5U. B) GS-FSC curve of the map associated with pdb_00009T5U. C) cFSC curves of the map associated with pdb_00009T5U. D) Viewing direction distribution of the particle stack. E) Relative signal amount for each of the viewing directions.

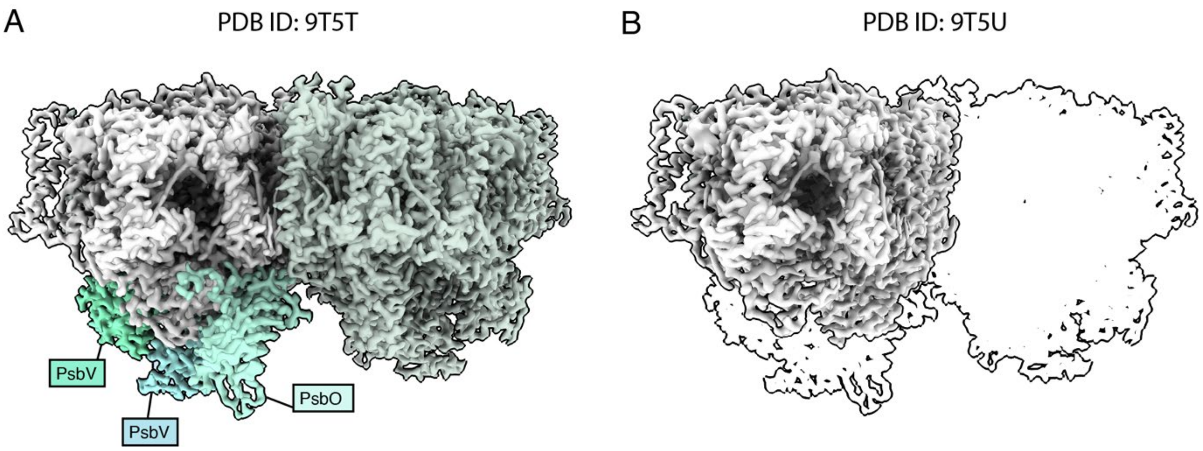

fig. S3 – Comparison between the two structures presented

A) Map related to pdb_00009T5T for the dimeric FR-PSII from *C. thermalis.* The portions of the map that are absent in pdb_00009T5U are highlighted in different shades of green. B) Map related to pdb_00009T5U for the monomeric FR-PSII from *Calothrix* sp. The outline of pdb_00009T5T is overlaid for clarity.

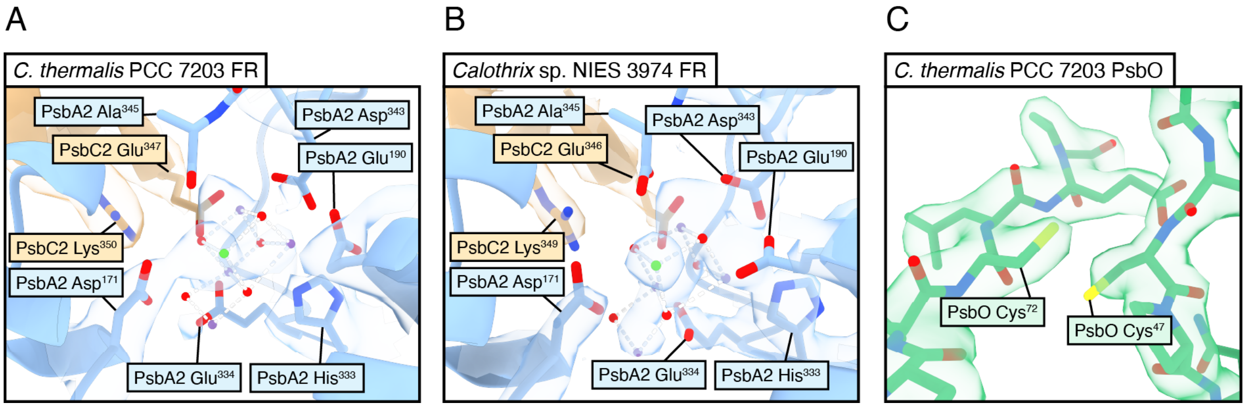

fig. S4 – OEC

A) Model and density snapshot of the oxygen evolving complex in *C. thermalis.* B) Model and density snapshot of the oxygen evolving complex in *Calothrix* sp.. C) Broken disulfide bond in PsbO from *C. thermalis* indicating electron damage.

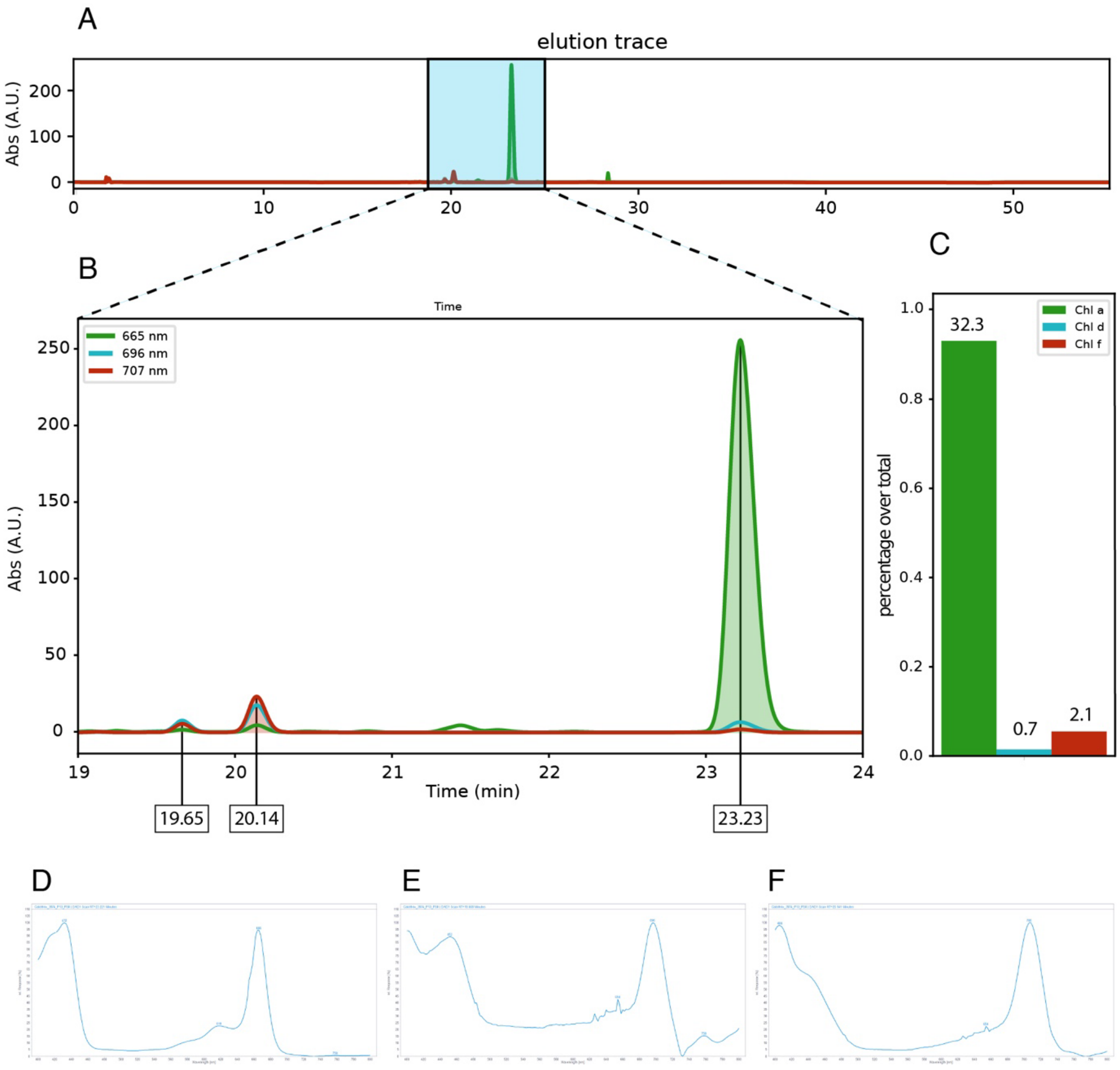

fig. S5 – HPLC pigment quantification analysis

A) Elution trace of an HPLC run performed on the FR-PSII samples from *Calothrix* sp. B) Zoom on the section in which Chl molecules eluted. C) Quantification of the area under the elution peaks. D) Absorbance spectra of the fraction eluting at 23.23 minutes, corresponding to the spectra of Chl *a*. E) Absorbance spectra of the fraction eluting at 19.65 minutes, corresponding to the spectra of Chl *d*. F) Absorbance spectra of the fraction eluting at 20.14 minutes, corresponding to the spectra of Chl *f*.

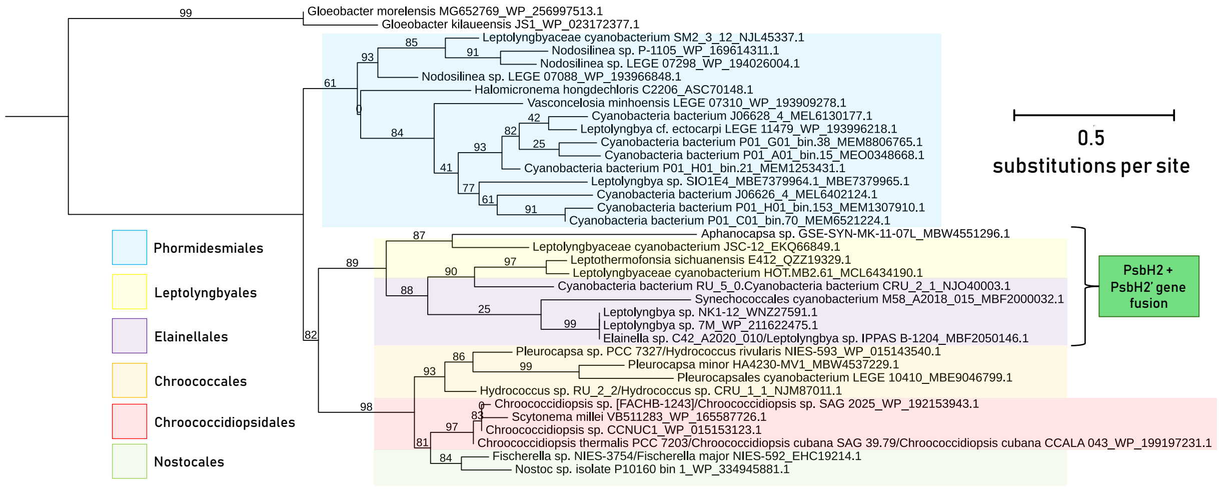

fig. S6 – Maximum Likelihood phylogenetic trees of PsbH2' sequences

Maximum likelihood tree of PsbH2' sequences inferred using IQ-TREE2 (v2.3.6) [41]. Branch supports correspond SH-aLRT support values [43]. PsbH2' tree rooted using an outgroup of Gloeobacter PsbH C-termini sequences. Taxonomic annotations based on GTDB and 16s rRNA taxonomy [14,52]. Sequences with PsbH2 and PsbH2' gene fusion indicated. Tree figures exported from TreeViewer [53].

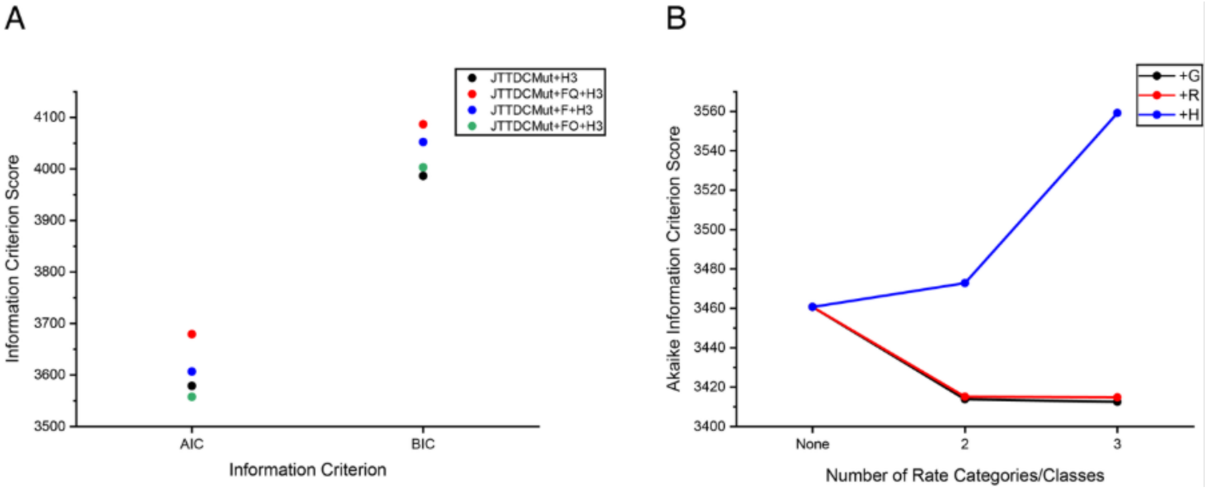

fig. S7 – Akaike Information Criterion scores for tested phylogenetic models

A) Akaike Information Criterion (AIC) and Bayesian Information Criterion (BIC) scores for various descriptors of equilibrium frequencies for PsbH2'. Values were obtained using ModelFinder[42]. Each descriptor of equilibrium frequencies was paired with the JTTDCMut substitution matrix and three heterotachy classes. +FQ corresponds to equal frequencies, +F to empirical frequencies and +FO to optimized frequencies. B) Akaike Information Criterion scores for various descriptors of substitution rate heterogeneity for PsbH2'. Akaike Information Criterion scores were retrieved through model parameter optimization in IQ-TREE2 (v2.3.6) with the -n 0 flag. Each descriptor of substitution rate heterogeneity was paired with the LG substitution matrix[55] and optimized equilibrium frequencies (+FO). +G corresponds to Gamma rate. +R corresponds to Free rates. +H corresponds to heterotachy classes.

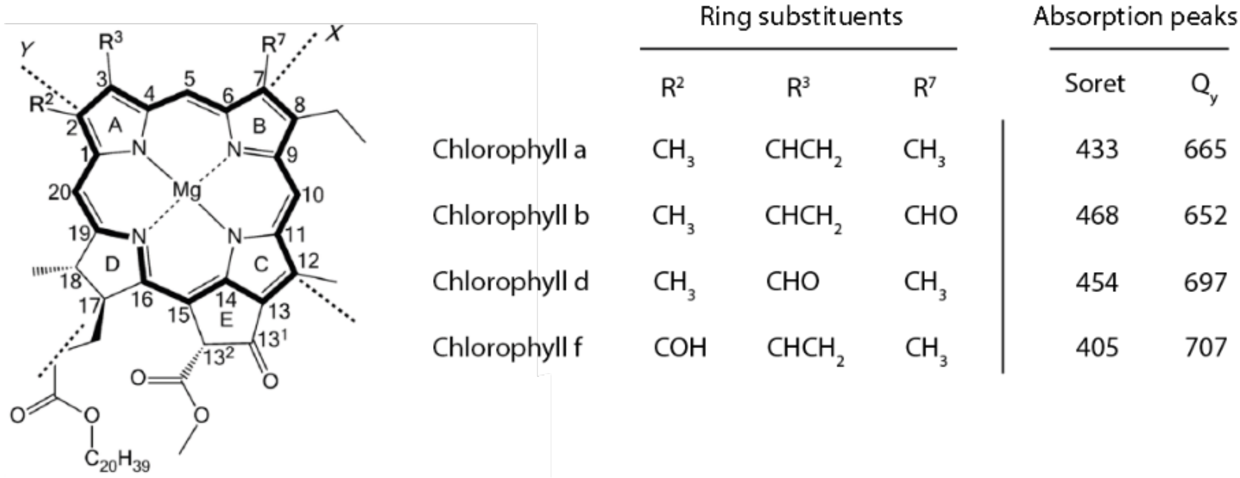

	Ring substituents			Absorption peaks	
	R²	R³	R⁷	Soret	Q_y
Chlorophyll a	CH_3	$CHCH_2$	CH_3	433	665
Chlorophyll b	CH_3	$CHCH_2$	CHO	468	652
Chlorophyll d	CH_3	CHO	CH_3	454	697
Chlorophyll f	COH	$CHCH_2$	CH_3	405	707

fig. S8 – Structural differences among Chl *a*, Chl *b*, Chl *d* and Chl *f*

A chlorophyll tetrapyrrole backbone is shown with positions of the key substituents indicated. For each chlorophyll, the side table lists the chemical groups at each position (R2, R3, R7) and the corresponding absorption maxima in the Soret band and Q_y transition measured in methanol.

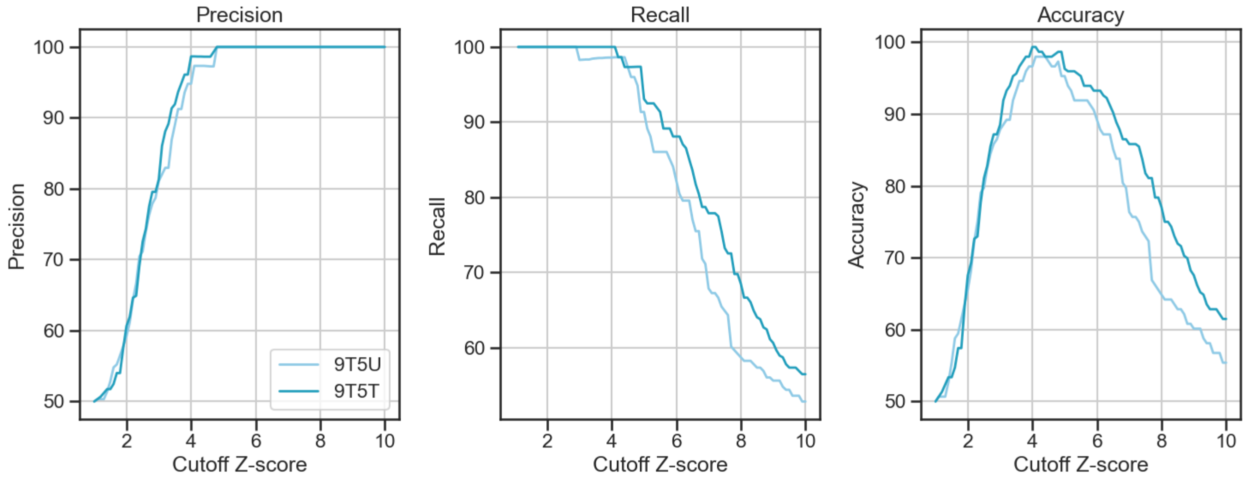

fig. S9 – Precision, Recall and Accuracy metrics of the maps and models presented

Evaluation of the cone-scan ESP analysis for two map/model combinations. Precision, recall, and accuracy were calculated across a range of Z-score threshold from 1 to 10 to assess the reliability of substituent classification. The Z-score cutoff maximizing accuracy is around 4 σ, providing a quantitative measure of the quality of the analysis for each map/model combination.

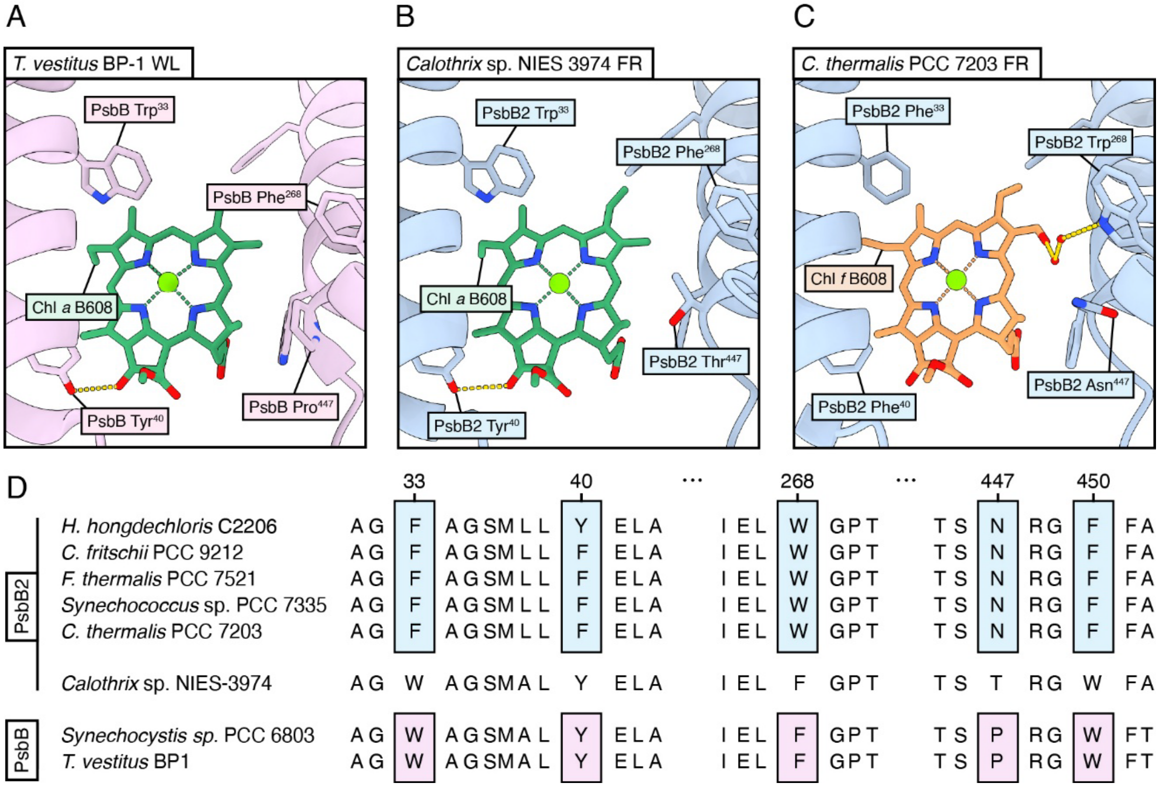

fig. S10 – Chl *f* B608 site

A) Atomic model around the B608 site in *T. vestitus* BP-1. Chl *a* is represented in green, and WL PsbB is represented in pink. H-bonds indicated as yellow dotted lines. B) Atomic model around the B608 site in *Calothix* sp. FR-PSII. Chl *a* is represented in green, and FR PsbB2 is represented in light blue. H-bonds indicated as yellow dotted lines. C) Atomic model around the B608 site in *C. thermalis* FR-PSII. Chl *f* is represented in orange, and FR PsbB2 is represented in light blue. H-bonds indicated as yellow dotted lines. D) Multiple sequence alignment of PsbB and PsbB2 in the Chl B608 binding region. Conserved changes that support the presence of a Chl *f* in the site are highlighted.

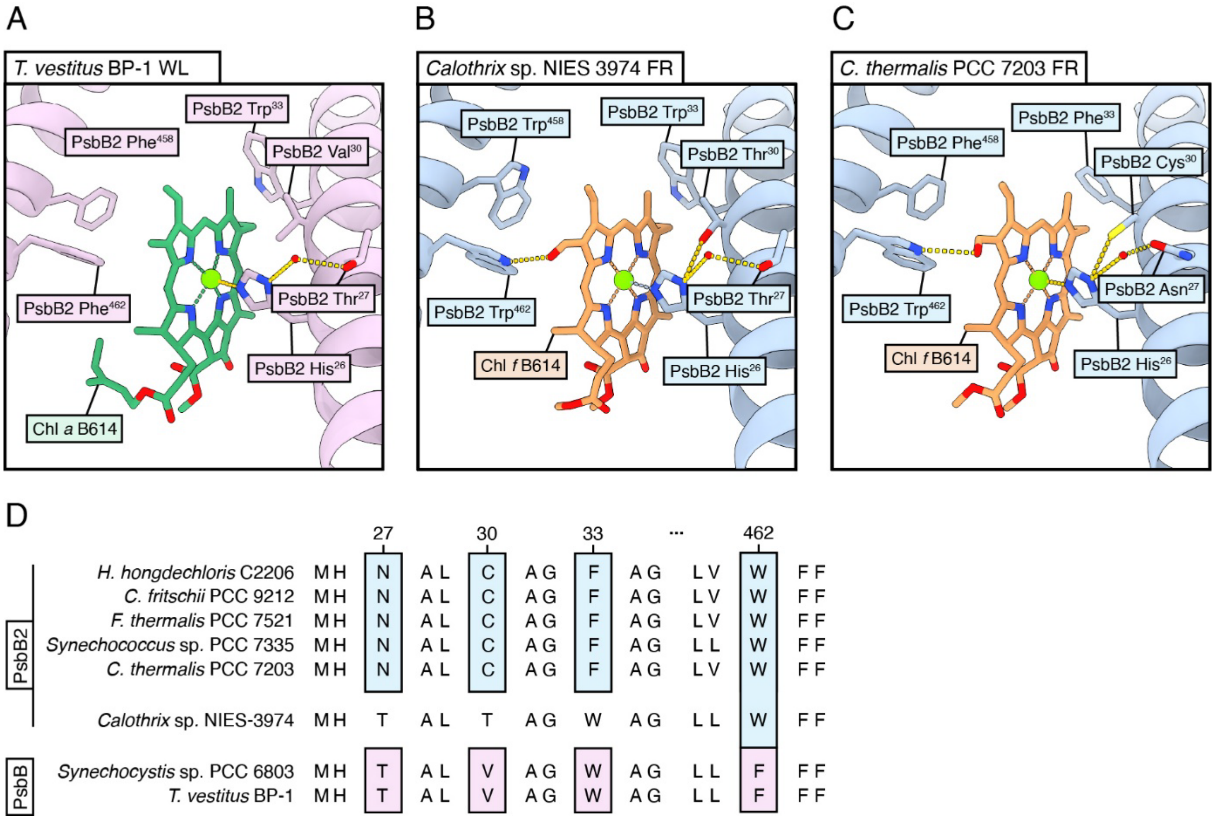

fig. S11 – Chl *f* B614 site

A) Atomic model around the B614 site in *T. vestitus* BP-1. Chl *a* is represented in green, and WL PsbB is represented in pink. H-bonds indicated as yellow dotted lines. B) Atomic model around the B614 site in *Calothrix* sp. FR-PSII. Chl *f* is represented in orange, and FR PsbB2 is represented in light blue. H-bonds indicated as yellow dotted lines. C) Atomic model around the B614 site in *C. thermalis* FR-PSII. Chl *f* is represented in orange, and FR PsbB2 is represented in light blue. H-bonds indicated as yellow dotted lines. D) Multiple sequence alignment of PsbB and PsbB2 in the B614 coordinating region. Conserved changes that support the presence of a Chl *f* in the site are highlighted.

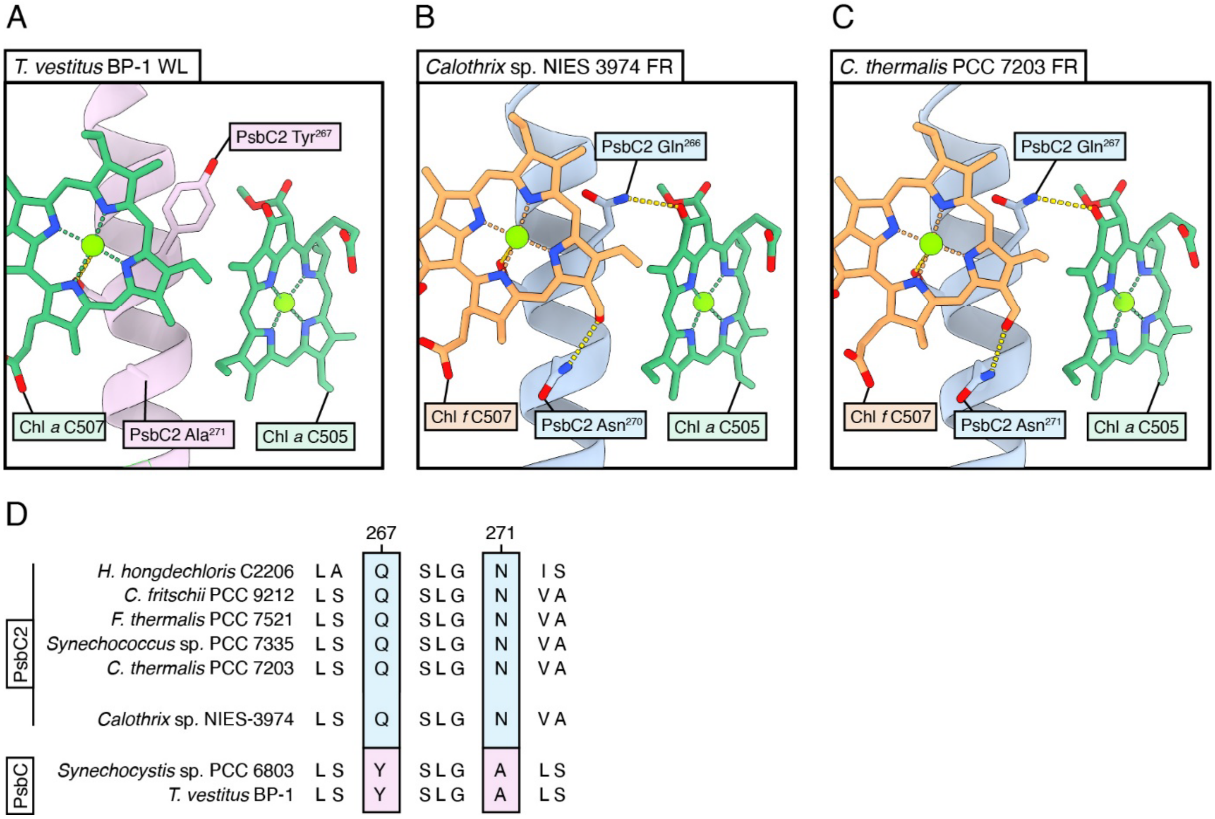

fig. S12 – Chl *f* C507 site

A) Atomic model around the C507 site in *T. vestitus* BP-1. Chl *a* is represented in green, and WL PsbC is represented in pink. B) Atomic model around the C507 site in *Calothix* sp. FR-PSII. Chl *f* is represented in orange, Chl *a* is represented in green, and FR PsbC2 is represented in light blue. H-bonds indicated as yellow dotted lines. C) Atomic model around the C507 site in *C. thermalis* FR-PSII. Chl *f* is represented in orange, Chl *a* is represented in green, and FR PsbC2 is represented in light blue. H-bonds indicated as yellow dotted lines. D) Multiple sequence alignment of PsbC and PsbC2 in the C507 region. Conserved changes that support the presence of a Chl *f* in the site are highlighted.

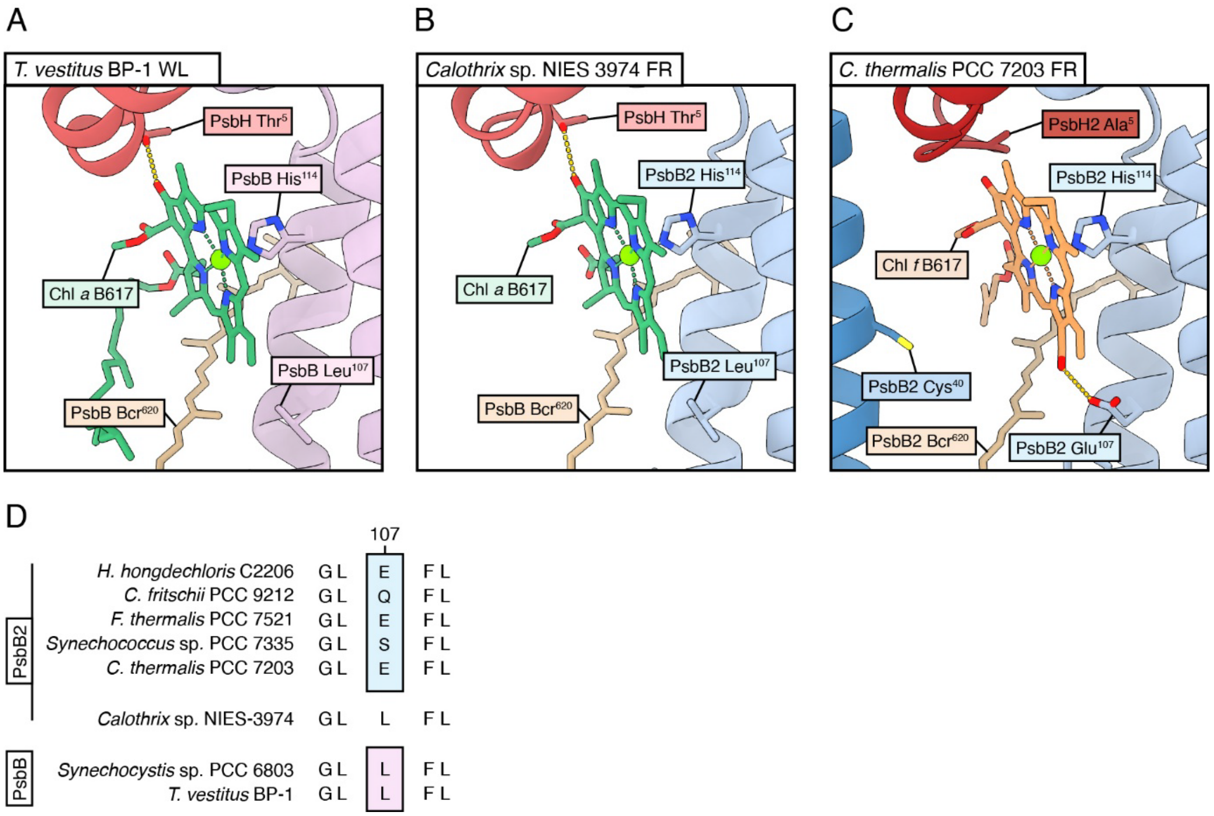

fig. S13 – Chl *f* B617 site

A) Atomic model around the B617 site in *T. vestitus* BP-1. Chl *a* is represented in green, PsbH in salmon, and WL PsbB is represented in pink. B) Atomic model around the B617 site in FR *Calothix sp*. Chl *a* is represented in green, PsbH in salmon and FR PsbB2 is represented in light blue. C) Atomic model around the B617 site in FR *C. thermalis*. Chl *f* is represented in orange, FR PsbH2 in red and FR PsbB2 is represented in light blue. D) Multiple sequence alignment of PsbB and PsbB2. Conserved changes that support the presence of a Chl *f* in the site are highlighted.

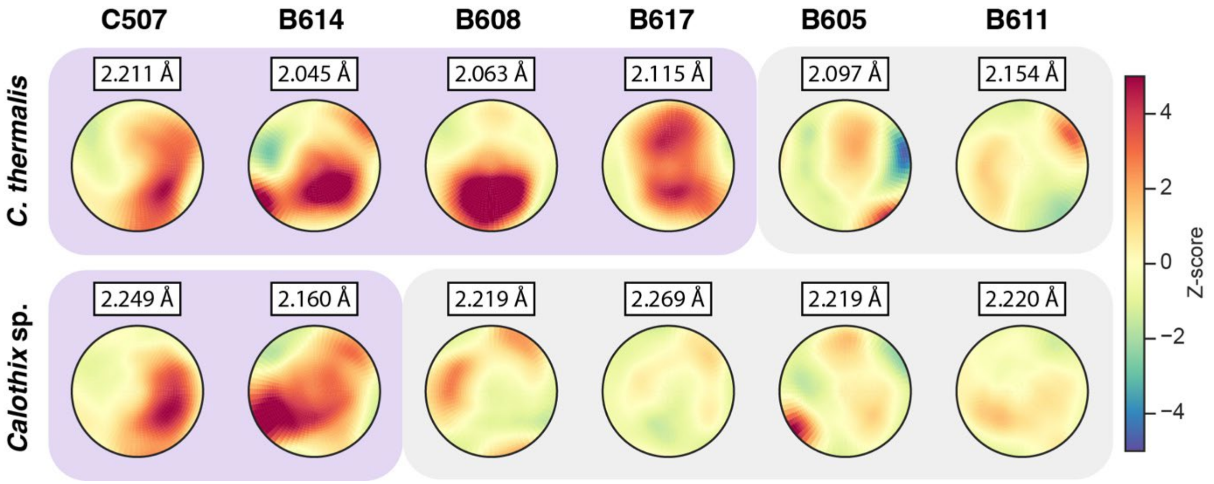

fig. S14 – Cone scan analysis of the pdb_00009T5T and pdb_00009T5U ESP maps and models

A) ESP quantification of the C2 formyl groups of Chl *f* candidates presented from the structural models and associated maps from pdb_00009T5T and pdb_00009T5U. The local resolution of the Mg atoms is displayed above the scan for each of the Chl sites. For further description see materials and methods.

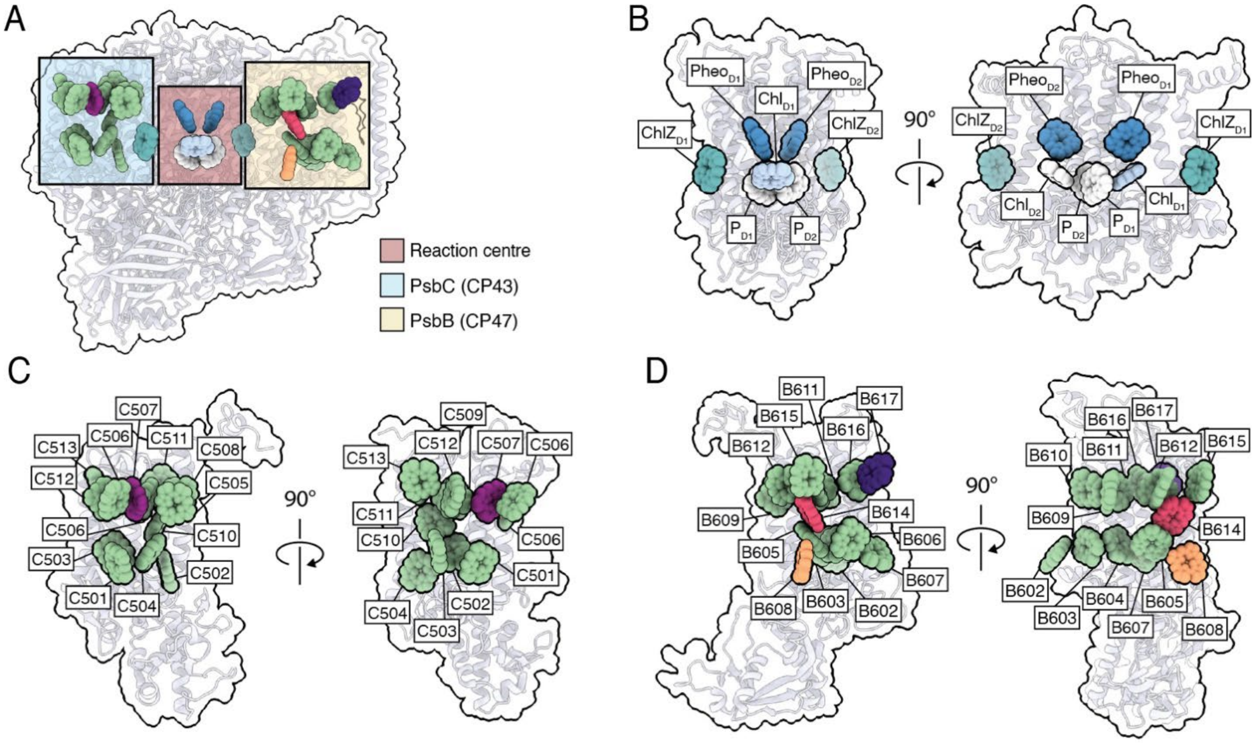

fig. S15 – *C. thermalis* FR-PSII chlorophyll sites in FR-PSII

A) General composition of a FR-PSII monomer from *C. thermalis*, antenna and reaction center positioned as in colored boxes in legend. Antenna chlorophyll *a* molecules colored in green, reaction center chlorophylls in gray, pheophytin *a* molecules in dark blue, chlorophyll *d* in light blue, and chlorophyll *f* molecules colored as in Fig. 3. B) Chlorophyll positions in the reaction center subunits of FR-PSII. C) Chlorophyll positions in the CP43 PsbC2 subunit of FR-PSII. D) Chlorophyll positions in the CP47 PsbB2 subunit of FR-PSII. (A-D) Views shown relative to the membrane plane.

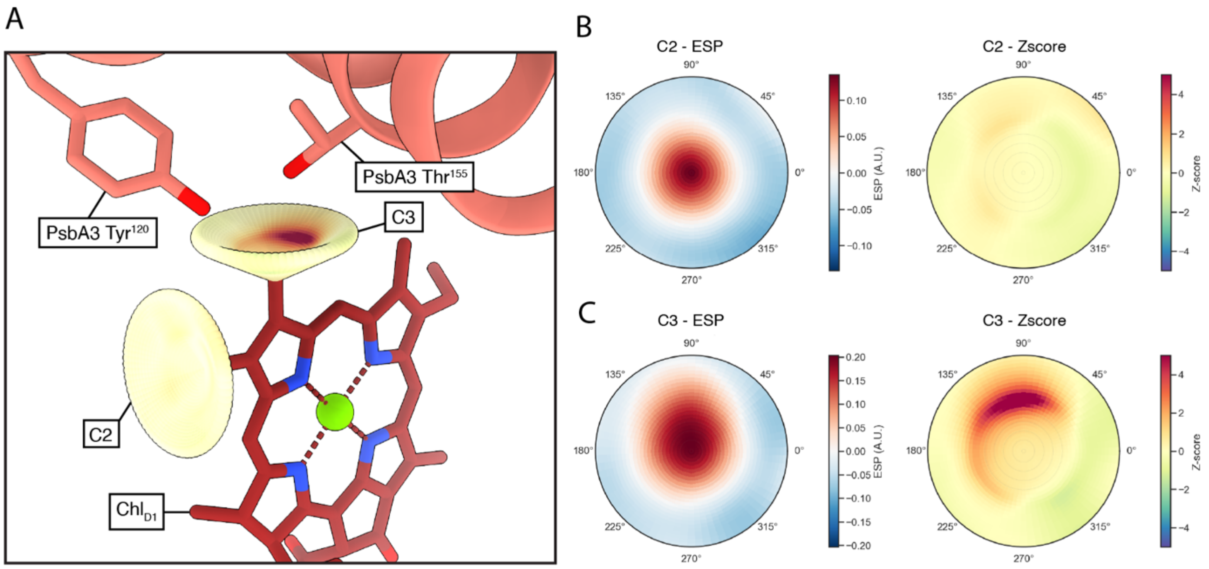

fig. S16 – Cone scan analysis of the substituents of Chl$_{D1}$

A) Chemical environment of Chl$_{D1}$ in FR-PSII from *C. thermalis*. The PsbA3 backbone is represented in pink and Chl$_{D1}$ is represented in red. The cone scans in the positions C2 and C3 of the ring are colored according to their ESP Z-score. B) Cone scans of the raw ESP and Z-score of the C2 substituent of Chl$_{D1}$. C) Cone scans of the raw ESP and Z-score of the C3 substituent of Chl$_{D1}$.

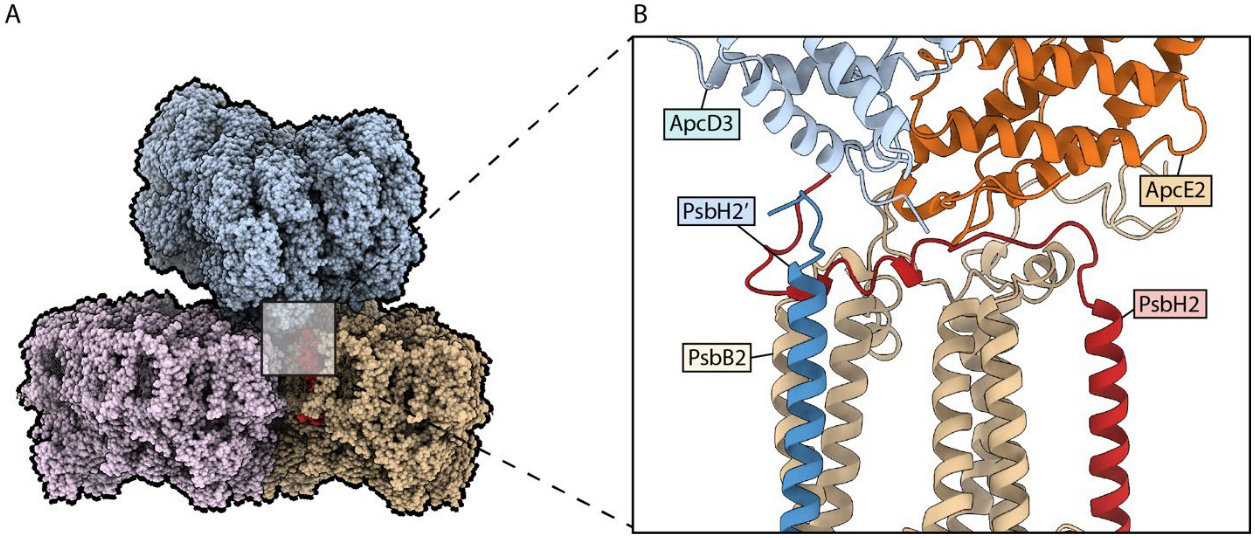

fig. S17 – Relative position of PsbH2 and PsbH2' to the FR-APC

A) Representation of the FR-PSII – FR-APC supercomplex rigid-body-fitted to the *A. platensis* PSII-APC supercomplex (pdb_00008WQL) [26]. B) Detail of the possible interactions between the terminal emitter subunits of the FR-APC (ApcE2 and ApcD3) with the PsbH2 and PsbH2' subunits.

Supplementary Note 1: Structural description of FR-PSII structures

Structure comparison between pdb_00009T5T and pdb_00009T5U

The ESP of the dimeric FR-PSII from *C. thermalis* shows density for all expected subunits in an intact PSII, including the integral membrane subunits PsbA3, PsbB2, PsbC2, PsbD2, PsbE, PsbF, PsbH2, PsbI, PsbJ, PsbK, PsbL, PsbM, PsbT, PsbX, PsbY, PsbZ, Ycf12, the far-red exclusive subunit PsbH2', and the extrinsic subunits PsbO, PsbU and PsbV (Table S3). The map also shows density for all expected cofactors, including 60 chlorophyll *a* molecules, 8 chlorophyll *f* molecules, 2 chlorophyll *d* molecules, 4 pheophytin *a* molecules, 4 plastoquinone molecules, 2 bicarbonate molecules, 2 non-heme iron, 2 heme *b*, 2 heme *c*, 4 chloride ions, 2 manganese-calcium clusters and several lipids and water molecules.
The ESP of the monomeric FR-PSII from *Calothrix* sp. shows density for subunits including integral membrane subunits PsbA, PsbB, PsbC, PsbD, PsbE, PsbF, PsbH, PsbI, PsbJ, PsbK, PsbL, PsbM, PsbT, PsbX, PsbY, PsbZ, and Ycf12. Extrinsic subunits PsbO, PsbU and PsbV are absent (Table S3). The map also shows density for cofactors including 32 chlorophyll *a* molecules, 2 chlorophyll *f* molecules, 1 chlorophyll *d*, 2 pheophytin *a* molecules, 2 plastoquinone molecules, 1 bicarbonate molecule, 1 non-heme iron, 1 heme *b*, 1 chloride ion, 1 manganese-calcium cluster and several lipids and water molecules.

The ESP at the oxygen-evolving complex (OEC) from pdb_00009T5T shows density for four manganese ions and one calcium ion, with interatomic distances consistent with ones from an OEC reduced and damaged by the electron beam during data collection (fig. S4A). Congruent with this is the reduction of the disulfide bond in PsbO (fig. S4C), between residues Cys^{47} and Cys^{72} [48].
In pdb_00009T5U, despite the absence of the extrinsic subunits, the ESP suggests low occupancy of the C-terminal portion of PsbA and the OEC. Water molecules associated with Mn_4 and Ca are alsonot detected. ESP is present for a calcium ion and all four manganese ions, with interatomic distances larger than ones expected of an intact, undamaged OEC (fig. S4B).

Supplementary Note S2: Phylogenetic analysis of the PsbH2’ subunit

Phylogenetic model testing and reproducibility

Evaluation of PsbH2’ as a dataset shows that biologically plausible topologies can be retrieved despite limited phylogenetic information. Particularly for difficult datasets with low signal, maximum likelihood trees lack reproducibility even with the same arguments [54]. To test if trees were reproducible, 10 independent runs were performed for various models (Table S4) using the same random seed and tree search parameters.

Despite being penalized by the Akaike Information Criterion (fig. S7), the use of heterotachy classes was necessary for congruence with the species tree [12–14] and reproducibility. While formal model testing did not yield a consensus on how equilibrium frequencies should be modelled, the congruent and reproducible topology (fig. S6) was obtained only by using empirically derived frequencies from the JTTDCMut matrix [56] (Table S4), suggesting that model parameter estimation is prone to overfitting. A higher stochastic Nearest Neighbor Interchange perturbation strength of 0.7 [41] was necessary to converge on the same topology that is also congruent with the species tree (Table S4). This more congruent topology had a superior log-likelihood than those obtained using lower perturbation strengths suggesting that the tree search algorithm can get trapped in local optima associated with artefactual trees.
These observations show that while PsbH2’ represents a difficult phylogenetic dataset, congruence with the species tree can be achieved by model evaluation.

On the origins of PsbH2’

We found that the C-terminal region of some *Gloeobacter* PsbH sequences preferentially aligned with PsbH2’ sequences when aligning PsbH2 and PsbH2’ sequences for a variety of alignment algorithms [39,57–59]. In the PsbH2’ phylogeny, these C-terminal regions were inferred to be sister to all PsbH2’ sequences and supported as a possible outgroup by root testing. In line with evidence that FaRLiP was present in the last common ancestor of all extant photosynthetic Cyanobacteriota [7,60,61], we suggest the possibility that PsbH2’ arose from the re-functionalization of the C-terminus of an ancestral PsbH allele.

We consider the possibility of *de novo* gene functionalization or a scenario in which run-on transcription of an ancestral *psbH* gene results in functionalization of previously non-genic DNA. In the latter case, the ancestral population from which all extant FaRLiP systems derive would have had genes for fused and unfused PsbH2 isoform, with each allele being differentially inherited by later-branching cyanobacterial orders. These propositions stand only if the C-terminal region of the *Gloeobacter* PsbH was not derived as an autapomorphy.

Supplementary Note S3: Chl *f* site assignments

Chlorophyll *a*, *b*, *d* and *f* differ from one another only in the substituents of their tetrapyrrole ring. Substituents on the ring of Chl *a* are oxygenated to form other Chl species, specifically to a formyl group at position C3 (Chl *d*), C2 (Chl *f*) or C7 (Chl *b*). These minimal chemical differences require high local resolution to be unambiguously detected in ESP maps.

Moreover, several intrinsic properties of cryo-EM complicate the confident identification of formyl groups:

1) Differently from X-ray crystallography, which provides information on electron density directly, Cryo-EM maps are generated from 2D projections of the Coulomb potential (ψ) of the imaged proteins according to:

$$\psi(r) = q/4\pi r\varepsilon$$

   where q is the charge, ε is the permittivity of the medium, and r is the distance from the charge. Because $\psi(r)$ depends linearly on charge, positively charged nuclei contribute positively to the signal, while surrounding electrons contribute negatively, partially shielding the nucleus[62]. Consequently, electronegative atoms such as the oxygen of the formyl groups of Chl d and *f* contribute less strongly to the overall potential[11]. Even in high-resolution ESP maps, negatively polarized side chains often appear attenuated, and formyl oxygens in previously reported cryo-EM structures have frequently been weak or undetectable. This intrinsic signal attenuation makes discrimination between methyl and formyl substituents particularly challenging.

2) The electron beam can trigger decarboxylation events during the irradiation, leading to a loss of signal corresponding to the Chl f C2 formyl oxygen in later frames of the movies[63,64]. Signal loss related to beam damage is partially compensated during the motion correction steps, in which dose weighting techniques are employed to retrieve high-resolution information prevalently in earlier frames, while relying on later frames only for lower resolution information.

3) For formyl groups that are not hydrogen bonded, the formyl oxygen can adopt a wider distribution of torsion angles where its energy will be minimised, therefore diluting the signal over the two different positions in plane with the tetrapyrrole ring of the chlorophyll[11,17].

Taken together, these factors explain why identification of Chl *f*, based solely on the presence of a formyl oxygen in ESP maps, requires cautious interpretation, high local map quality and multiple independent lines of evidence. In the present work, assignments are not made solely on the basis of ESP analysis, but are supported by complementary structural, phylogenetic, and spectroscopic observations. In particular, conserved amino acid substitutions in far-red light paralogues

compared with their white light counterparts surrounding key chlorophyll substituents (e.g., hydrogen bonding at C2 and changes in Mg-coordinating residues) are taken into account[21].

Within this framework, the cone-scan ESP analysis (See material and methods), is used as a statistical tool to identify substituents that have an electrostatic potential deviating significantly from that expected for a methyl group. The results of this analysis should not be treated as prescriptive in defining pigment identity, since differences in ESP can arise from multiple factors, including map quality, model fitting, and local resolution, and not exclusively from the presence of an additional formyl oxygen. Therefore, interpretation of the ESP analysis must consider the overall map quality and structural context, and other lines of evidence to reliably assign Chl *f* sites.

In previous work [11,17], a fixed cutoff of 3σ was used to classify substituents as significantly different from a methyl distribution. In the present study, a more formal statistical framework to determine an appropriate Z-score threshold for each map/model combination was implemented. To assess the quality of the analysis and to identify an optimal classification cutoff, confusion matrices were computed across a range of Z-score thresholds, and for each, precision, recall, and accuracy were calculated.

To construct the confusion matrix, substituents common to Chl *a* and *f* were used as internal references: C3 and C8 (non-methyl substituents) are expected to be significantly different from the methyl distribution, while C7 and C12 (methyl substituents) are expected not to differ from the methyl distribution.

For a given Z-score threshold, True Positives (TP) were defined as non-methyl substituents with ESP values outside the methyl normal distribution. False Positives (FP) as methyl substituents with ESP values outside the methyl normal distribution. True Negatives (TN) as methyl substituents not outside the methyl normal distribution and False Negatives (FN) as non-methyl substituents not outside the methyl normal distribution.

Precision represents the percentage of correctly assigned non-methyl substituents over the total number of positive assignments, so a high precision is indicative of a low number of FP assignments:

$$\text{Precision} = \frac{\text{TP}}{\text{TP} + \text{FP}}$$

Recall represents the percentage of correctly assigned non-methyl substituents over the total amount of non-methyl substituents, so a high recall is indicative of a low number of FN assignments:

$$\text{Recall} = \frac{\text{TP}}{\text{TP} + \text{FN}}$$

Finally, accuracy represents the percentage of correctly classified substituents for a given Z-score threshold and therefore a high accuracy means a low number of misclassified substituents (FP+FN):

$$\text{Accuracy} = \frac{\text{TN} + \text{TP}}{\text{TN} + \text{FP} + \text{TP} + \text{FN}}$$

In this instance of pdb_00009T5T and pdb_00009T5U, the statistical analysis showed that a Z-score cutoff of 4 yields the highest accuracy (fig. S9) and was therefore chosen as the cutoff value for the ESP analysis.

Chl *f* B608

Phylogenetic and ESP analysis confirms that, in *C. thermalis*, Chl B608 is a Chl *f* [4]. The Mg^{2+} of this Chl is coordinated by a water molecule, in a network involving the residue PsbB2 Glu^{41} and the carbonyl backbone of PsbB2 Gly^{59} as in WL-PSII. The C2 formyl group of Chl B608 is connected to the far-red specific residue PsbB2 Trp^{268} via two water molecules in a chain of hydrogen bonds. In addition, a far-red specific residue PsbB2 Asn^{447} is present. Although not in hydrogen bonding distance in the structure, Asn^{447} could provide further stabilization of the two water molecules in the dynamic protein environment. Analysis of the ESP map around the C2 group of this site indicates the presence of a formyl oxygen. In contrast, *Calothrix* sp. retains the white light conserved PsbB residues and presents a Chl *a* molecule in this site.

Chl *f* B614

Chl B614 has been suggested to be a Chl *f* site from previous structural and phylogenetic data [3,4,18]. Analysis of the ancestral sequence reconstruction of PsbB in FaRLiP-capable organisms suggests this Chl *f* site as one of the first to be evolved, highlighting its importance in the function of FR-PSII [4,18]. Some fully conserved far-red specific changes are present in the vicinity of the C2 substituent of Chl B614 in both species. A Phe present in WL-PSII is substituted with PsbB2 Trp^{462}, acting as a H-bond donor for the C2 formyl oxygen. The C2 formyl group also shows increased electrostatic potential in the cryoEM map, strengthening the assignment as a formyl group. The Chl in this site presents a peculiarity compared to other sites - its central Mg^{2+} ion is coordinated by PsbB2 His^{26}, and not by a water molecule or a more polar residue as would be expected for Chl *f*.

Nonetheless, the delta N-H of PsbB His^{26} is engaged in a H-bond with a water molecule that is in turn H-bonded to either a FR-conserved PsbB Asn^{27} in *C. thermalis* and a number of other FR-PSII; or PsbB Thr^{27}, in *Calothrix* sp. and WL-PSII. It is important to note that in FR-PSII, a residue, either PsbB Cys^{30} in *C. thermalis* and other species, or PsbB Thr^{30} in *Calothrix* sp., replace PsbB Val^{30} in WL-PSII, acting as a H-bond donor to the delta N. The additional H-bond would likely influence the electron delocalization in the His and change the basicity of the epsilon N. This could tune the properties of PsbB His^{26} and make it more polar than it naturally is, affecting its selectivity for a Chl *f*. . In addition, PsbB Cys^{30} could also potentially alter the site energy or properties of the site. The H-bond geometry between the CMB/OMB atoms and PsbB Trp^{462} in C. thermalis appears suboptimal. However, the cone-scan analysis reveals increased C2 ESP density oriented towards Trp^{462}, supporting the presence of a formyl group engaged in hydrogen bonding (or at least, favorable electrostatic interactions). Together, these observations are consistent with the formyl substituent interacting with PsbB2 Trp^{462}.

Chl *f* C507

The Chl *f* containing site at C507 has been already described in the literature for all the structures available from *Synechococcus* sp. PCC 7335. It presents phylogenetically conserved changes that allow the incorporation of a Chl *f* molecule [3,4,18] – the far-red specific PsbC Asn[271] (an Ala in WL-PSII) provides the H-bond partner to the C2 formyl moiety (~3.3 Å). *Calothrix* sp. presents the same conserved changes as *C. thermalis* and presents a Chl *f* in this site.

As observed by Gisriel et al., the environment of an adjacent Chl *a* is also modified [18]. A far-red conserved interaction, originating from PsbC Gln[267], provides a H-bond to the 13^1-keto group of Chl *a* C505. This type of interaction is hypothesized to result in a lowering of the Chl *a* site energy, with a consequent redshift of the absorption profile [65]. This feature is likely to be important for efficient energy transfer to the reaction center.

Nature of the reaction center long wavelength chlorophyll: Chl *d* or Chl *f*?

In FR-PSII, the primary donor (Chl_{D1}) was initially identified as a long wavelength Chl, based on multiple lines of biophysical evidence [2]. First, from an energy levels perspective, the enhancement in the intensity of the thermoluminescence emission peak indicated a decrease in the energy gap between the charge separated and excited state of the reaction center, consistent with a lower-energy, longer-wavelength primary donor/emitter. Second, from a spectroscopic point of view, the changes in the light-minus-dark difference spectra indicated the disappearance of a blue shift assigned to the canonical Chl_{D1} Qy absorption at ~680 nm together with the appearance of a marked blue shift in a pigment around 720 nm, indicating a long wavelength pigment in position Chl_{D1}. From a phylogenetic point of view, the conserved amino acid changes clearly showed two possible H-bonding groups that could potentially bind either the formyl group from a Chl *f* in the C2 position or to the formyl group from a Chl *d* in the C3 position of Chl_{D1} [2]. Spectroscopically, the absorption peak of Chl_{D1} was assigned to 727 nm. Consequently, since Chl *d* is generally considered to be more blueshifted than Chl *f*, and the wavelength (727 nm) of the primary donor was similar to the Chl *d* (725 nm) in this location in *Acaryochloris*, a working hypothesis was that the Chl_{D1} site harbors a Chl *d*.

Nonetheless, given the presence of both Chl *d* and Chl *f* in FR-PSII, the exact nature of the long wavelength pigment in the PSII reaction center could not be addressed at the time. Previous structures of FR-PSII, and more recently computational approaches, have provided new insight on this, agreeing in the assignment of a Chl *d* primary donor but without the precise H-bonding network that stabilizes the reaction center Chl *d* [3,4,8,18]. In the map presented, the improved resolution together with the cone scan analysis has allowed us to rule out the presence of a Chl *f* in position D1, since the substituent does not show any increased ESP around the C2 position (fig. S16B). Moreover, the increased ESP is clearly oriented towards PsbA3 Thr[155] rather than towards PsbA3 Tyr[120], suggesting the potential hydrogen bond network that stabilizes Chl *d* in this position.

The suggestion that the long wavelength Chl in the reaction center could be located at the P_{D2} site rather than at the Chl_{D1} site, based on experimental ambiguities in the light-minus-dark difference spectrum, can be ruled out due to the absence of conserved phylogenetic changes None of the other reaction center Chl presents an increased ESP at the C2 substituent, and there are no amino acids capable of stabilizing a putative formyl group at the position C3 of Chl P_{D2}. The amount of

evidence in favor of Chl *d* being present in position Chl_{D1} allows a confident assignment of the long wavelength pigment to this position.

Data collection

Microscope	Krios III
Camera	Falcon 4i
Magnification	155000x
Voltage (kV)	300
Electron exposure (e-/Å^2)	40
Defocus range (μm)	-0.8 to -2
Pixel size (Å)	0.723
Energy filter	Selectris (20 eV)
Exposures	23311
Image format	EER

Data processing

Box size	650 px
Initial particles (no.)	423204
Final particles (no.)	49763
Symmetry	C2
Map resolution (Å)	2.17
Map sharpening *B* factor	-40.3

Model Refinement

Refinement package	PHENIX
Initial model used	pdb_00008EQM
Real/reciprocal space	Real Space
Resolution cutoff	2.20
MolProbity score	1.33
Clash score	6.04
Ramachandran outliers	0.00 %
Ramachandran allowed	1.39 %
Rotamer outliers	0.23 %
CaBLAM outliers	0.99 %

Table S1 – Data acquisition, processing and model refinement parameters for pdb_00009T5T

Data collection

Microscope	Krios G3i
Camera	K3 Bioquantum
Magnification	105000x
Voltage (kV)	300
Electron exposure (e-/Å^2)	60
Defocus range (μm)	-0.4 to -1.8
Pixel size (Å)	0.827
Energy filter slit	20 eV
Exposures	17792
Image format	EER

Data processing

Box size	600 px
Initial particles (no.)	1,427,197
Final particles (no.)	285,844
Symmetry	C1
Map resolution (Å)	2.33
Map sharpening *B* factor	-63.7

Model Refinement

Refinement package	PHENIX
Initial model used	pdb_00009T5T
Real/reciprocal space	Real Space
Resolution cutoff	2.40
MolProbity score	1.33
Clash score	5.96
Ramachandran outliers	0.00 %
Ramachandran allowed	1.68 %
Rotamer outliers	0.17 %
CaBLAM outliers	0.90 %

Table S2 – Data acquisition, processing and model refinement parameters for pdb_00009T5U

Subunit	Homologue	9T5T	9T5U
PsbA3	FR	Present	Present
PsbB2	FR	Present	Present
PsbC2	FR	Present	Present
PsbD2	FR	Present	Present
PsbE	WL	Present	Present
PsbF	WL	Present	Present
PsbH2	FR	Present	Not in genome
PsbH2’	FR	Present	Not in genome
PsbJ	WL	Present	Present
PsbK	WL	Present	Present
PsbI	WL	Present	Present
PsbL	WL	Present	Present
PsbM	WL	Present	Present
PsbO	WL	Present	Absent
PsbP	WL	Present	Absent
PsbQ	WL	Present	Present
PsbY	WL	Present	Present
PsbT	WL	Present	Present
PsbU	WL	Present	Absent
PsbV	WL	Present	Absent
PsbW	WL	Present	Absent
Ycf12	WL	Present	Present
PsbX	WL	Present	Present
PsbZ	WL	Present	Absent
PsbA	WL	Absent	Absent
PsbB	WL	Absent	Absent
PsbC	WL	Absent	Absent
PsbD	WL	Absent	Absent
PsbH	WL	Absent	Present

Table S3 – Subunits present in the maps and models

PSII subunits present in the pdb_00009T5T and pdb_00009T5U models associated with this work. Each subunit is marked as present or absent in the model and whether the subunit is encoded in the FaRLiP cluster of the organismand expressed during FaRLiP. WL indicates subunits that are not found in the respective FaRLiP cluster and shared in both the WL-PSII and FR -PSII complexes. Subunits are numbered according to the annotations in the *C. thermalis* genome.

Model	Pers	Average Normalized RF Distance	Unique Topologies (Out of 10)	Phormidesmiales topology	First branching
JTTDCMut+G3	0.4	0	1	Paraphyly	Leptolyngbyales/ Elainellales
JTTDCMut+FO+G3	0.4	0.006061	2	Paraphyly	Leptolyngbyales/ Elainellales
JTTDCMut+H3	0.4	0	1	Paraphyly	Leptolyngbyales/ Elainellales
JTTDCMut+FO+H3	0.4	0.028283	2	Paraphyly	Leptolyngbyales/ Elainellales
JTTDCMut+G3	0.7	0	1	Paraphyly	Leptolyngbyales/ Elainellales
JTTDCMut+FO+G3	0.7	0	1	Paraphyly	Leptolyngbyales/ Elainellales
JTTDCMut+H3	0.7	0	1	Monophyly	Phormidesmiales
JTTDCMut+FO+H3	0.7	0	1	Paraphyly	Leptolyngbyales/ Elainellales
JTTDCMut+F+H3	0.7	0	1	Paraphyly	Leptolyngbyales/ Elainellales
JTTDCMut+FQ+H3	0.7	0	1	Paraphyly	Leptolyngbyales/ Elainellales

Table S4 – Reproducibility of PsbH2' maximum likelihood trees using the same tree search parameters for different models

Reproducibility measured using averaged normalized Robinson-Foulds (RF) distance of 10 independent tree searches in IQ-TREE2 (v2.3.6) [41] using the exact same tree search parameters. Stochastic Nearest Neighbor Interchange perturbation strength values are shown (pers 0.4 and pers 0.7).

Stochastic NNI Perturbation Strength	Unique Topologies (Out of 10)	Log-likelihood
0.4	1	-1561.439
0.5	1	-1565.287
0.7	1	-1548.167

Table S5 – Best log-likelihood values of PsbH2' maximum likelihood trees inferred under the JTTDCMut+H3 model using different Nearest Neighbor Interchange perturbation strengths

Best log-likelihoods of 10 independent tree searches in IQ-TREE2 (v2.3.6) [41] using the same tree search parameters except different Stochastic Nearest Neighbor Interchange (NNI) perturbation strength.

Chl[a]	3WU2[b]	6DHE[c]	7SA3[d]	8EQM[d]	9T5T[b]	9T5U[b]
P_{D1}	A405/a409	A404/a405	A403	A404	A405	A405
P_{D2}	D402/d402	D403/d403	D403	D403	D402	D402
Chl_{D1}	A406/a410	A411/d402	A404	D401	A406	A406
Chl_{D2}	A407/a411	A405/a406	A405	A405	A407	A407
$Pheo_{D1}$	A408/a412	A406/a407	A406	A406	A408	A408
$Pheo_{D2}$	A409/a413	D402/d401	D402	D402	A409	A409
$Chlz_{D1}$	A410/a414	A407/a408	A407	A407	A410	A410
$Chlz_{D2}$	D403/d403	D404/d404	D404	D404	D403	D403
B1	B602/b604	B601/b602	-	B601	B602	B602
B2	B603/b605	B602/b603	B601	B602	B603	B603
B3	B604/b606	B603/b604	B602	B603	B604	B604
B4	B605/b607	B604/b605	B603	B604	B605	B605
B5	B606/b608	B605/b606	B604	B605	B606	B606
B6	B607/b609	B606/b607	B605	B606	B607	B607
B7	B608/b610	B607/b608	B606	B607	B608	B608
B8	B609/b611	B608/b609	B607	B608	B609	B609
B9	B610/b612	B609/b610	B608	B609	B610	B610
B10	B611/b613	B610/b611	B609	B610	B611	B611
B11	B612/b614	B611/b612	B610	B611	B612	B612
B12	B613/b615	B612/b613	B611	B612	B613	B613
B13	B614/b616	B613/b614	B612	B613	B614	B614
B14	B615/b617	B614/b615	-	B614	B615	B615
B15	B616/b618	B615/b616	B613	B615	B616	B616
B16	B617/b619	B616/b617	B614	H101	B617	B617
C1	C501/c902	C501/c501	C501	C502	C501	C501
C2	C502/c903	C502/c502	C502	C503	C502	C502
C3	C503/c904	C503/c503	C503	C504	C503	C503
C4	C504/c905	C504/c504	C504	C505	C504	C504
C5	C505/c906	C505/c505	C505	C506	C505	C505
C6	C506/c907	C506/c506	C506	C507	C506	C506
C7	C507/c908	C507/c507	C507	C508	C507	C507
C8	C508/c909	C508/c508	C508	C509	C508	C508
C9	C509/c910	C509/c509	C509	C510	C509	C509
C10	C510/c911	C510/c510	C510	C511	C510	C510
C11	C511/c912	C511/c511	C511	C512	C511	C511
C12	C512/c913	C512/c512	C512	C513	C512	C512
C13	C513/c914	C513/c513	C513	C514	C513	C513

Table S6 – Conversion table for chlorophyll nomenclature between different PDB structures.

Chl *f* assignments are colored in red, and chlorophyll *d* assignments are colored in blue.

[a] Recommended unified numbering [49]

[b] Each monomer (uppercase and lowercase chains) has its own numbering in pdb_00003WU2. In this manuscript, the numbering from the uppercase chains is used [50]

[c] Each monomer (uppercase and lowercase chains) has its own numbering in pdb_00006DHE [51].

[d] Note that although pdb_00007SA3 and pdb_00008EQM use a different numbering system than pdb_00003WU2, the publications that describe these structures use the numbering system (uppercase chains) from pdb_00003WU2 in writing. - indicates that the chlorophyll is absent in the structure.

48. Kato, K. et al. High-resolution cryo-EM structure of photosystem II reveals damage from high-dose electron beams. Commun. Biol. 4, (2021).
49. Müh, F. & Zouni, A. Structural basis of light-harvesting in the photosystem II core complex. Protein Science 29, 1090–1119 (2020).
50. Umena, Y., Kawakami, K., Shen, J.-R. & Kamiya, N. Crystal structure of oxygen-evolving photosystem II at a resolution of 1.9 Å. Nature 473, 55–60 (2011).
51. Kern, J. et al. RT XFEL structure of the dark-sSupplementary Table tate of Photosystem II (0F, S1-rich) at 2.05 Angstrom resolution. Worldwide Protein Data Bank https://doi.org/10.2210/pdb6dhe/pdb (2018) doi:10.2210/pdb6dhe/pdb.
52. Parks, D. H. et al. GTDB: an ongoing census of bacterial and archaeal diversity through a phylogenetically consistent, rank normalized and complete genome-based taxonomy. Nucleic Acids Res. 50, D785–D794 (2022).
53. Bianchini, G. & Sánchez-Baracaldo, P. TreeViewer: Flexible, modular software to visualise and manipulate phylogenetic trees. Ecol. Evol. 14, (2024).
54. Shen, X.-X., Li, Y., Hittinger, C. T., Chen, X. & Rokas, A. An investigation of irreproducibility in maximum likelihood phylogenetic inference. Nat. Commun. 11, 6096 (2020).
55. Le, S. Q. & Gascuel, O. An Improved General Amino Acid Replacement Matrix. Mol. Biol. Evol. 25, 1307–1320 (2008).
56. Kosiol, C. & Goldman, N. Different Versions of the Dayhoff Rate Matrix. Mol. Biol. Evol. 22, 193–199 (2005).
57. Sievers, F. et al. Fast, scalable generation of high-quality protein multiple sequence alignments using Clustal Omega. Mol. Syst. Biol. 7, (2011).
58. Edgar, R. C. Muscle5: High-accuracy alignment ensembles enable unbiased assessments of sequence homology and phylogeny. Nat. Commun. 13, 6968 (2022).
59. Lassmann, T. Kalign 3: multiple sequence alignment of large datasets. Bioinformatics 36, 1928–1929 (2020).
60. Kim, T. D., Pretorius, D., Murray, J. W. & Cardona, T. Exploring the Structural Diversity and Evolution of the D1 Subunit of Photosystem II Using AlphaFold and Foldtree. Physiol. Plant. 177, (2025).
61. Cardona, T., Murray, J. W. & Rutherford, A. W. Origin and Evolution of Water Oxidation before the Last Common Ancestor of the Cyanobacteria. Mol. Biol. Evol. 32, 1310–1328 (2015).
62. Marques, M. A., Purdy, M. D. & Yeager, M. CryoEM maps are full of potential. Curr. Opin. Struct. Biol. 58, 214–223 (2019).
63. Wang, J. On the appearance of carboxylates in electrostatic potential maps. Protein Science 26, 396–402 (2017).
64. Wang, J. & Moore, P. B. On the interpretation of electron microscopic maps of biological macromolecules. Protein Science 26, 122–129 (2017).
65. Llansola-Portoles, M. J. et al. Tuning antenna function through hydrogen bonds to chlorophyll a. Biochimica et Biophysica Acta (BBA)-Bioenergetics 1861, 148078 (2020).
