# Supplementary material for: Mapping the absorption landscape of far-red Photosystem II
Source: Nat Commun. 2026 Jun 6;17:7223. doi: 10.1038/s41467-026-73964-7 (PMC13396731; doi:10.1038/s41467-026-73964-7)

nature portfolio

Peer Review File

Mapping the absorption landscape of Far-Red Photosystem II

Corresponding Author: Professor A Rutherford

Parts of this Peer Review File have been redacted as indicated to remove third-party material.

This file contains all reviewer reports in order by version, followed by all author rebuttals in order by version.

Attachments originally included by the reviewers as part of their assessment can be found at the end of this file.

Version 0:

Reviewer comments:

Reviewer #1

(Remarks to the Author)
The manuscript reports cryo-EM structures of two far-red photosystem II complexes from different organisms. Critical results include the confident identification of far-red chlorophylls (d and f), specifically their number and location in the two different PSII structures, and the detection of a previously unidentified protein subunit (PsbH2') that is far-red exclusive and contributes to the binding of a Chl f pigment. Spectroscopic analysis was able to disentangle and assign specific wavelengths to the far-red pigments.

The work is performed at the highest standard combines several methodological approaches (structural investigation, phylogenetic analysis, spectroscopy) to paint a complete and compelling picture of the systems. It provides substantial new insight into the mechanisms, structural basis, but also variability in far-red photoacclimation. The manuscript is well-referenced and the discussion highlights clearly the broader implications, importance, and connections (e.g. Chl f is found at a specific position hypothesized as the red-most chlorophyll of white light PSII). It is certain that the present study will have a significant impact in the field and will motivate further research, particularly in terms of modeling excitation energy transfer pathways.

A point that can be considered concerns clarifications about the labeling/numbering of chlorophylls to facilitate comparisons with the literature. Since it might not be immediately possible to correlate labels used here with other PSII models, it could be useful to indicate the standardized nomenclature for CP43 and CP47 proposed in Protein Sci. 2020;29:1090 and provide a supporting table listing corresponding nomenclature in major structures.

A figure with side-on views of each of the major proteins showing all chlorophylls individually labeled would also aid orientation.

Is any correlation with computational studies of WL-PSII models meaningful? [J Chem Phys. 2022;156(21):215101, Chem Sci. 2021;12(12):4463, Chem Sci. 2024;15(19):7269].
Have there been attempts at simulating similar FR-PSII systems?

The connection to FR-APC is interesting but I do not find the argumentation and statements on pages 10,11 entirely clear. Is it eventually proposed that Chl f C507 is the most plausible excitation injection point, whereas Chl f B617 serves as an excitation energy dissipation point? How was the alignment (docking?) of FR-PSII and FR-APC performed, and is there a fitness score for the two positions shown in Figure 4?

Reviewer #2

(Remarks to the Author)
In this manuscript, the authors have provided high-resolution (2.17/2.33 Å) cryo-EM structures of the far-red type PSII from two cyanobacteria: Chroococcidiopsis thermalis PCC 7203 and Calothrix sp. NIES-3974, complementing the previous

structural studies of Synechococcus sp. The authors identified a new far-red–exclusive subunit, PsbH2', and used the ESP approach to refine the site assignment to Chl f. Their method used genetic diversity from FaRLiP as a basis for determining the site energy shown in the comparison (Fig 3). The discovery of PsbH2, as well as the modelling of FR-APC (Fig 4), are both significant contributions to the understanding of oxygenic photosynthesis.

At the same time, several major questions require certain clarification.

Major questions.
1. Since both Chroococcidiopsis thermalis PCC 7203 and Calothrix sp. NIES-3974 have been cultivated entirely under far-red light for more than three months, please clarify the following:
(a) Is there any evidence that WL-PSII can be found or formed in any of these two strains after long-term growth in a far-red light environment, or does FaRLiP completely replace WL-PSII?
(b) Without direct structural comparisons between WL-PSII from either of the two strains and FaRLiP from both (i.e., the structural characteristics of WL-PSII and FaRLiP do not overlap), what are the criteria for identifying the alterations specific to far-red light adaptation (i.e., alterations such as PsbH2', Chl f binding sites, and sequence variations) attributable to the respective species, C. thermalis and Calothrix?
(c) Does the conclusion drawn from a comparative analysis of the two structures of Far-Red PSII presuppose that both structures are in the same physiological state (i.e., pure FaRLiP), or do species differences confound the FaRLiP interpretation?

2. Could the authors provide additional information regarding their rationale for the selection of C. thermalis and Calothrix species as subjects for their studies. Additionally, was the presence of a monomeric PSII a priori apparent in Calothrix, or did it arise during a particular cultivation or purification technique?

3. The manuscript identifies PsbH2' as a PSII subunit specific to far-red light. Could the authors tell us if PsbH2' has been identified in isolation from PsbH2 in any of the FaRLiP cyanobacteria? Or is PsbH2' always associated with PsbH2 (including fusion proteins)?

4. The HPLC analysis (Fig. S5) shows that the pigment compositions (Chl a/d/f) are highly consistent with previous reports. However, could you please provide additional information on any biochemical/proteomic techniques you may have used to verify the far-red PSII subunit composition at the protein level (e.g., mass spectrometry, immunoblotting)? Or was the identity of these subunits inferred largely from their genomic context and/or their densities in cryo-EM images?

5. I would like to request a table that clearly outlines which subunits of PSII in each reviewed organism exhibit a far-red change or modification, as well as which core PSII subunits are conserved with respect to white-light PSII in these studied organisms, so as to clarify the findings of the authors in a more conclusive manner.

6. Fig. 3A and 3B illustrate the application of Gaussian deconvolution to the absorbance spectra, with the fitted residuals shown; however, there is very little detail added to the methods section about the fitting procedures or the validations carried out to assess the methods. Could the authors provide more detailed information on the software used for model fitting, the fitted constraints, and how 'goodness-of-fit' was determined?

7. The study describes the radiation-induced damage done, including the breakdown of disulfide bonds in PsbO (Fig. S4C) and changes in OEC interatomic distances (Fig. S4A/B), but does not include a discussion of the effects of these radiolytic degradation products on assignments of Chl f using ESP analysis.
I would suggest that the authors provide clarifications on a few points:
(a) Are there any limitations relating to beam effects on chlorophyll formyl groups?;
(b) Are the Chl f ESP signals stable at the applied doses?;
(c) Can you provide a dose-fractionation analysis or add a discussion?

8. Fig. 3F and Supp. Text S3 reference papers/GitHub for the 4σ Z-score cutoff on Chl C2 classifications (Chl f vs. a) do not provide a rigorous empirical basis for using 4σ as opposed to other alternative cutoff values (i.e., Chl C2 = 3σ or Chl C2 = 3.5σ), nor do they provide any form of sensitivity/specificity on the results of using this cutoff and without confidence thresholds, nor do they confirm the use of known Chl a locations as being used for confirming corrected Chl a classifications for the data generated during the development of this work.
Please provide additional information or cite another document in your response.

9. Differential Chl f content was identified using HPLC (Fig. S5), further supporting the proposed structures.
However, the methods did not provide the following:
(a) retention time of the Chl a/d/f plus standard compounds;
(b) evidence that the retention of Chl d (696nm) and Chl f (707nm) was fully resolved;
(c) use of peak integration/baseline integration to quantify the concentrations;
(d) provides replicates (n=?) for all measurements and uncertainty (mean ± std);
(e) co-elution samples for Chl d and Chl f.
A complete record of this information is essential to ensure reproducibility of the results.

10. Your wavelengths have been assigned based solely on your comparison between C. thermalis (dimeric with four Chl f (completely extrinsic subunits)) and Calothrix sp. (monomeric with two Chl f (with PsbO, PsbU, and PsbV absent)).
For this comparison to be valid, all of the following must be addressed:

(a) Were the proteins PsbO, PsbU, and PsbV not present in the native Calothrix FR-PSII, or did they get removed during purification with 0.4% β-DM?
(b) When you removed the extrinsic subunits, did it not affect the water oxidation complex structure, the coordination of the Chl pigments, or any water networks?
(c) What are the reasons that you can conclude that the loss of the peaks at 721 and 749 nm is due to the absence of Chl f and not to structural changes caused by purification?
(d) If you compare a completely dimeric structure (C. thermalis) to an incompletely monomeric structure (Calothrix), should Calothrix also possess complete extrinsic proteins to allow a valid comparison?
(e) Have you looked at the absorption spectra of intact Calothrix cells or thylakoids derived from Calothrix sp. NIES-3974 to determine if your purified Calothrix structure resembles that of the native Calothrix?

Minor issues.
1. Could you please add the scale bar to Fig. 1 and the panels in the proper way?
2. Fig. 1 D – the authors use the term “Stromal”. Perhaps it could be a bit more accurate to use the term “cytoplasmic” for cyanobacteria.
3. Fig. 3 in the title, a small typo - “assignment”. Please change to “assignment”
4. Fig. S9 – In the figure description, we see “FRL-PSII”. Is it the same as FR-PSII used in the main manuscript? Please correct or define.
5. Grid preparation – the authors mentioned “Gold quantifoil 2 μm hole size and 1 μm hole spacing” Did they mean Quantifoil Au R2/1, 300 mesh?
6. The microscope used in the acknowledgement part is specified as Krios G3i, but in the Data collection table, as Krios III. Please use consistent nomenclature throughout.
7. Please check the particle number consistency throughout the text and tables again.

In summary, this study offers new structural information for a far-red light-responsive form of PSII with atomic resolution and provides valuable information on how organisms can adapt their photosynthetic functon(far-red adaptation) under changing conditions (far-red light intensity), even if certain aspects of this study require additional explanation and clarification with respect to methodological aspects.
Therefore, I recommend this manuscript for publication in Nature Communications upon satisfactory revisions.

Reviewer #3

(Remarks to the Author)
The manuscript by Leong and coworkers describes new results regarding the molecular layout and specific properties of photosystem II (PSII) from far-red adapted organisms. The authors combine structural insights based on cryo electron microscopic studies and results from spectroscopic and phylogenetic analysis to describe the energy transfer network used in the red light adapted version of PSII. The authors describe the structure of far red PSII from C. thermalis 7203 at 2.17 Å and from Calothrix 3974 at 2.33 Å resolution using single particle cryo electron microscopy.
In the Thermalis structure the position of 4 Chl f in the antenna system of the complex was described, 3 of them matching with previous reports from structures of FR adapted PSII from other organisms and one assigned differently from previous work. In addition, the presence of a new subunit, PsbH2', that has not been described before is described. In contrast, the Calothrix structure does not have PsbH2' and only shows two Chl f molecules in the antenna system. By directly comparing spectroscopic data for both together with the structural information Leong et al are able to specifically assign the different far red absorption bands in both complexes to specific Chl f molecules. In addition the authors suggest a model of the cofactor arrangement in a far red adapted PSII allophycocyanin super-complex and depicted energy transfer pathways from the phycobilin external antenna towards the reaction center involving specific Chl f molecules that show red shifted spectral properties.
Several recent studies have also targeted similar far-red acclimated versions of PSII and this is a very active field of investigation in the photosynthesis research community, trying to decipher the changes in the entire photosynthetic apparatus, including PSII, Photosystem I and the antenna complexes upon shift from normal to far red light conditions.

Assignment of Chl f:
A previous study by Gisriel and colleagues already provided assignment of four Chl f in the far red light adapted PS II complex from Synechococcus 7335. While the authors of the current manuscript state that the assignment in that work (ref. 4, Gisriel et al, 2023) was not conclusive based on electrostatic potential maps for all four Chl f, the Gisriel paper actually shows ESP scans for all four Chl f and provides assignment of these based on the scans. The local resolution for Chl f 507 in Gisriel et al 2023 was reported to be only 2.74 Å and hence the ESP scans are less conclusive compared to the high resolution data presented in the current manuscript. But the authors here should maybe more clearly state that previous work also used ESP scans for assignment of four Chl f. Also it would be nice to report the local resolution of the current data for each of the Chl f that are discussed in the manuscript so that it is easier for the reader to appreciate the local data quality.
The authors describe the possible location of a water next to Ser B107 in the Synechococcus structure by Gisriel et al as an indication that the Chl B617 could also be a Chl f in Synechococcus. Does this imply that the authors conclude that the Chl f assignment by Gisriel and colleagues in their Synechococcus RL structure is not correct? Do you suggest that instead one of the other Chl's assinged to Chl f by Gisriel et al should be instead assigned as Chl a to keep the number of Chl f at 4 in both the Synechococcus and the Thermalis structure? If I understand it correctly the authors suggest that Chl B605 is not a Chl f in Synechoccus. But is surprising that Chl B605 in the Synechococcus structure showed the clearest result of the cone scanning procedure and has also the highest local resolution reported. If at the same time this Chl was wrongly assigned as Chl f, as indicated in the current manuscript, then it becomes questionable if the cone scanning method is actually useable to decide about the nature of Chl in these ESP maps. The authors should comment on this.

The fig. 2 showing the location of a Chl f at position B617 needs to be improved. The figure or the figure legend should state that you are actually showing data for C. thermalis here. The enlarged view shown in panel C should actually be connected with panel B and not with panel A as the view direction is similar in B and C (maybe you could swap orientation in A and B?). Panels D and E are not clear for non-expert readers. I suggest if space permits to include parts of figure S13 into figure 2 to illustrate the density scanning approach that is utilized in the present manuscript to decide about Chl identity. Accordingly it would be good to include some text in the main manuscript explaining the density scanning and the resulting polar and Z score plots. Also it might be nice to show the structures of Chl a, Chl d and Chl f in either the main text figures or in the SI as many non expert readers might not know the subtle structural differences between them.
In figure 3 E the monomeric structure of Calothrix 3974 PSII is shown but the legend says dimeric. Please correct.
LHG416 (fig. 1) is not modelled well into the map, not sure if it can be clearly identified based on the map (see below). Please check and comment on this.

The additional PsbH2' subunit:
The reported identification of the additional subunit PsbH2' is very interesting. It seems to modulate the monomer-monomer interface and while no direct contacts are formed between PsbH2' with residues from the other monomer in a dimer its presence clearly reduces the volume in the monomer-monomer interface accessible to lipids. Are there any indications that the presence of PsbH2' influences the dimer stability? Could the authors compare the yields of dimeric and monomeric PSII obtained from samples under FR and WL conditions for C. thermalis?

Connection to the FR-APC:
It would be nice to actually show where PsbH and PsbH' is located in the APC-PSII complex to allow the reader to better follow the argument about the importance of PsbH' for better interaction with the FR form of APC. One caveat in the modeling is that the overall phycobilisome-PSII structure from a white light acclimated sample was used to fit the FR-APC and the FR-PSII together. It should be stated that there might be differences in the overall assembly upon FR acclimation that are not captured by the white light supercomplex.
The distances between ApcE2 and Chl f in the complex are discussed but it would be good to also describe/show the distance of terminal emitters from the APC to any other Chl a. Are the Chl f at a shorter distance compared to the closest Chl a? Also given the very long distance of >27 A exciton transfer is likely slow from ApcE2 to any of the Chl f. Would it be faster to a Chl a? Maybe good to discuss this. Are there any experimental data for the energy transfer times from APC to the core in white light versus far red light adapted complexes? This would be good to discuss here.

Comments on the SI:
Text of the SI in general should be checked carefully as there are many examples of bungled sentences or repetitions.
Section in the SI about nature of RC chlorophyll could move to the main text as well as fig. S13. Without that figure the panels D and E in Fig. 2 make no sense to the reader.
SI page 4, line 11: please include the reference for Modelangelo (Jamali e al, Nature 2024)
Chl f B614: H-bond to B Trp462 would result in an angle of 86 deg between CMB,OMB and Trp NE in the thermalis structure, not a likely geometry for an H-bond.
SI page 19 line 1-5 please rewrite this text as there are contradictory statements in these few sentences
SI page 19 line 12,13: "Calothrix sp. retains the white light conserved residues and the white light subunit PsbH and presents a Chl a molecule in this site."
This statement is very confusing as I thought from fig. S9 B that a Chl f was modeled in this site for Calothrix sp. Please explain/correct the text accordingly.
SI page 20, line 5: " ligand to the chlorophyll where the backbone carbonyl of PsbC Ser266."
Please fix this sentence
SI page 20, line 7:" The torsion angle of this residue is maintained by the presence of another conserved change, PsbC Gln267 (a Met in WL-PSII), that is likely to improve the stability of the site and possibly increase the electron withdrawing effect of PsbC Asn271 on the C2 formyl of Chl C507. "
This is not convincing. The two residues are about 9 A from each other and it is not clear how one could affect the torsion angle of the other over this distance. The other explanation for this change given further below is more convincing (changing site energy of the Chl a C505).

Comment on model quality:
The model provided does not seem to always be an optimum fit to the experimental ESP maps. Some examples are listed in the attached file and the authors should maybe reconsider parts of the modeling before final deposition.

Version 1:

Reviewer comments:

Reviewer #1

(Remarks to the Author)
The authors have done a great job at responding to all comments, providing clarifications where necessary, and revising the manuscript and SI accordingly. I have no further comments.

Reviewer #2

(Remarks to the Author)

I have reviewed the revised manuscript and the authors' rebuttal. I am satisfied that all of my concerns have been properly addressed. The key additions — particularly the confusion-matrix framework for the Z-score threshold, the updated HPLC data with individual peak spectra, the new subunit table, and the clarifications on the spectral comparison between the two species — strengthen the work considerably. The dedicated methods section on the Gaussian deconvolution and the deposited fitting script also improve reproducibility.
The lack of HPLC replicates is noted but acceptable given that the pigment data serve as supplementary evidence to the structural and spectroscopic conclusions.

I have no further concerns and recommend this manuscript for publication in Nature Communications.

Reviewer #3

(Remarks to the Author)
The authors have revised their manuscript thoroughly and addressed all major points raised. The detailed explanation now provided for the cone scanning method and the assignment of Chl f in their structures improved the accessibility of the work for non-specialists. The additional explanations provided regarding energy transfer between far red APC and the PSII core is also satisfactory and I support publication of the revised version.

Open Access This Peer Review File is licensed under a Creative Commons Attribution 4.0 International License, which permits use, sharing, adaptation, distribution and reproduction in any medium or format, as long as you give appropriate credit to the original author(s) and the source, provide a link to the Creative Commons license, and indicate if changes were made.
In cases where reviewers are anonymous, credit should be given to 'Anonymous Referee' and the source.
The images or other third party material in this Peer Review File are included in the article's Creative Commons license, unless indicated otherwise in a credit line to the material. If material is not included in the article's Creative Commons license and your intended use is not permitted by statutory regulation or exceeds the permitted use, you will need to obtain permission directly from the copyright holder.
To view a copy of this license, visit https://creativecommons.org/licenses/by/4.0/

REVIEWER COMMENTS

Reviewer #1 (Remarks to the Author):

The manuscript reports cryo-EM structures of two far-red photosystem II complexes from different organisms. Critical results include the confident identification of far-red chlorophylls (d and f), specifically their number and location in the two different PSII structures, and the detection of a previously unidentified protein subunit (PsbH2') that is far-red exclusive and contributes to the binding of a Chl f pigment. Spectroscopic analysis was able to disentangle and assign specific wavelengths to the far-red pigments.

The work is performed at the highest standard combines several methodological approaches (structural investigation, phylogenetic analysis, spectroscopy) to paint a complete and compelling picture of the systems. It provides substantial new insight into the mechanisms, structural basis, but also variability in far-red photoacclimation. The manuscript is well-referenced and the discussion highlights clearly the broader implications, importance, and connections (e.g. Chl f is found at a specific position hypothesized as the red-most chlorophyll of white light PSII). It is certain that the present study will have a significant impact in the field and will motivate further research, particularly in terms of modelling excitation energy transfer pathways.

1) A point that can be considered concerns clarifications about the labelling/numbering of chlorophylls to facilitate comparisons with the literature. Since it might not be immediately possible to correlate labels used here with other PSII models, it could be useful to indicate the standardized nomenclature for CP43 and CP47 proposed in Protein Sci. 2020;29:1090 and provide a supporting table listing corresponding nomenclature in major structures.

We agree with the reviewer that the standardized CP43 and CP47 nomenclature introduced by Muh and Zouni (2020) would facilitate comparison across PSII structures. However, all the previously published structural papers on FR-PSII have followed the nomenclature from PDB 3WU2 (previously 3ARC until updated and renumbered) (Umena et al doi.org/10.1038/nature09913). To maintain consistency with the existing FR-PSII literature (and to avoid potential re-numbering errors), we would like to retain the current nomenclature in the manuscript. Nevertheless, as suggested by the reviewer, to facilitate cross-comparison with other PSII models, we have now added a conversion table in the Supplementary Information (Table S6) listing the FR-PSII nomenclature, the suggested standard nomenclature and those used in other core depositions of PSII. We believe this provides clarity while preserving continuity with prior FR-PSII studies.

2) A figure with side-on views of each of the major proteins showing all chlorophylls individually labelled would also aid orientation.

In response to the reviewer's suggestion, we have provided a side-on figure (fig. S15) to aid orientation and facilitate comparisons. Side-on views with all chlorophylls individually labelled are inevitably more crowded; the figure provided is our best shot at minimising this problem.

3) Is any correlation with computational studies of WL-PSII models meaningful? [J Chem Phys. 2022;156(21):215101, Chem Sci. 2021;12(12):4463, Chem Sci. 2024;15(19):7269]. Have there been attempts at simulating similar FR-PSII systems?

As far as we are aware, there have been no published attempts at simulating the site energy of the pigments in FR-PSII. But that is mainly because, until the present study, there have been no reliable

evidence-based assignments of their wavelengths. The reviewer's question flags one of the key interests of the present work: it opens the door to calculations that relate the wavelengths of the chlorophylls to i) the protein environments that tune their wavelengths, and ii) their specific roles in excitation energy transfer. Calculations of this kind are of great interest to the field and are on-going with our collaborators.

4) The connection to FR-APC is interesting but I do not find the argumentation and statements on pages 10,11 entirely clear. Is it eventually proposed that Chl f C507 is the most plausible excitation injection point, whereas Chl f B617 serves as an excitation energy dissipation point?

The reviewer is justified to ask for clarification in those sections. We should have done better. But yes, we do propose that Chl *f* C507 is the most plausible excitation injection point from the FR-APC, and this agrees with the earlier suggestion of Mascoli et al. (DOI: 10.1038/s41477-020-0718-z). This conclusion leads to the suggestion that Chl *f* B617 may function as an excitation energy dissipation site, analogous to a suggested role of its counterpart in conventional PSII.

5) How was the alignment (docking?) of FR-PSII and FR-APC performed, and is there a fitness score for the two positions shown in Figure 4?

The FR-PSII–FR-APC model was not generated by docking. Instead, it was assembled by rigid-body alignment of FR-PSII (PDB 9T5T) and FR-APC (PDB 9I1R) onto the PSII - PBS supercomplex structure from *Athrospira platensis* (PDB 8WQL), solved by subtomagram averaging-guided single particle analysis and reported in Nature Communications (DOI: 10.1038/s41467-024-51460-0). The same alignment-based approach was previously employed in our earlier work (DOI: 10.1038/s42003-025-08326-y).
Accordingly, no docking procedure or independent fitting optimisation was performed for the two positions shown in Fig. 4. There is therefore no "fitness score" for these positions. The only quantitative metric available is the RMSD of the structural alignment between our models and the corresponding regions of the reference supercomplex used for alignment. This section has been modified to clarify the methodological approach used and the suggested roles for chlorophylls C507 and B617 in excitation energy transfer and energy dissipation.

Reviewer #2 (Remarks to the Author):

In this manuscript, the authors have provided high-resolution (2.17/2.33 Å) cryo-EM structures of the far-red type PSII from two cyanobacteria: Chroococcidiopsis thermalis PCC 7203 and Calothrix sp. NIES-3974, complementing the previous structural studies of Synechococcus sp. The authors identified a new far-red–exclusive subunit, PsbH2', and used the ESP approach to refine the site assignment to Chl f. Their method used genetic diversity from FaRLiP as a basis for determining the site energy shown in the comparison (Fig 3). The discovery of PsbH2, as well as the modelling of FR-APC (Fig 4), are both significant contributions to the understanding of oxygenic photosynthesis. At the same time, several major questions require certain clarification.

Major questions:

1) Since both Chroococcidiopsis thermalis PCC 7203 and Calothrix sp. NIES-3974 have been cultivated entirely under far-red light for more than three months, please clarify the following:

1(a) Is there any evidence that WL-PSII can be found or formed in any of these two strains after long-term growth in a far-red light environment, or does FaRLiP completely replace WL-PSII?

There is strong evidence that expression of WL paralogues of photosystems and allophycocyanin is fully suppressed following far-red light photoacclimation. Transcriptomic analyses have shown that, upon the transition from white light growth to far-red light growth, FaRLiP-associated genes are upregulated while WL-PSII and WL-PSI paralogues are effectively silenced (10.1126/science.1256963, 10.3389/fmicb.2019.00465).

Proteomic analysis of cells grown for longer periods in far-red light (weeks) showed a similar pattern of upregulating FaRLiP encoded proteins (~2-4 fold relative upregulated) and downregulating WL subunits (10.1186/s12864-019-5587-3). Comparing PSI and PSII subunit compositions between WL and FR light cells, the FaRLiP encoded subunits make up near identical percentages of reaction centers in FR light as the WL subunits compose in WL, again suggesting a near total replacement of WL subunits in FR photosystems by FaRLiP encoding genes. Consistent with these findings, long-term cultivation under far-red light results in functional replacement of WL-PSII and WL-PSI by their far-red counterparts, with no detectable formation of WL complexes in the low temperature fluorescence emission spectra (see Nurnberg et al 2018). Mass spectrometry analysis of previous samples we have isolated has also confirmed the absence of WL paralogues in the samples (unpublished).

1(b) Without direct structural comparisons between WL-PSII from either of the two strains and FaRLiP from both (i.e., the structural characteristics of WL-PSII and FaRLiP do not overlap), what are the criteria for identifying the alterations specific to far-red light adaptation (i.e., alterations such as PsbH2', Chl f binding sites, and sequence variations) attributable to the respective species, C. thermalis and Calothrix?

Criteria identifying far-red light specific changes:
The identification of far-red–specific alterations was first based on comparative sequence and genomic analysis: comparing sequences of FR-PSII subunits in the FaRLiP cluster with the "canonical" WL subunits. This was done before structural data was available and sequences-based models indicated near-identical structures with a series of conserved changes specific for the FR-PSII. This approach and model was validated as structural data became available, still leaning on sequence-based models.

The assignment of far-red–specific structural features in this work, even with its improved resolution, still relies on consistent sequence-level differences between WL and FR paralogues across multiple cyanobacterial species. For each far-red specific structural feature presented in this work, specific residues in the sequences of WL-PSII subunits are systematically substituted by conserved amino acids in their FR paralogues. This comparative framework allows us to confidently attribute each structural feature as characteristic of far-red photoacclimation rather than to species-specific variation. In addition, the chemical nature of these amino acid variations is analysed in terms of their potential functional role, for example in Chl *f* binding: e.g. H-bonding to the Chl-*f*-specific C2 formyl, group, and the known chemical preference of Chl *f* for non-standard Mg ligands

C. thermalis vs Calothrix:
The sequence-based approach described above, allowed us to identify *Calothrix* sp. as a rare outlier which lacked some far-red features that were conserved in other FR-PSII. We then obtained the species, grew it, isolated the FR-PSII, solved the structure and proved that Chl *a* was present in place of Chl *f* in 2 locations.

On comparisons to WL-PSII structures and species dependence:

It is important to note that the WL-PSII sequences of FaRLiP-capable strains are highly conserved compared to those of strains that do not possess FaRLiP (i.e. canonical WL-PSII which has been studied for decades). Therefore, solving the WL structures from the specific strains studied here would not be expected to reveal species-specific structural differences that are relevant to the conclusions of this work or relevant to far-red photoacclimation.

PsbH2′:
In the case of PsbH2′, this gene is included in the FaRLiP cluster and expressed only under far-red light conditions, though with limited discussion in the literature beyond its genomic existence. Our discovery of this subunit in FR-PSII from *C. thermalis* allows its possible role(s) to be determined now that its binding site is known.

1(c) Does the conclusion drawn from a comparative analysis of the two structures of Far-Red PSII presuppose that both structures are in the same physiological state (i.e., pure FaRLiP), or do species differences confound the FaRLiP interpretation?

The comparative analysis of the two FR-PSII structures assumes that both complexes were isolated from fully photoacclimated ("pure FaRLiP") cultures. As described in the Methods, cells were grown under strictly controlled far-red light conditions for several months prior to harvesting, ensuring complete induction of the FaRLiP acclimation before rapid isolation of the photosystems.

We do not consider species-specific differences are likely to confound the FaRLiP interpretation.

As discussed above, there is no evidence for mixed WL/FR-PSII complexes in this or previous work. In addition, as discussed in the manuscript (and in for PS1 in Consoli et al 2025), we consider that some of the conserved changes present in the FR paralogous subunits prevent binding of WL subunits into FR-Photosystems during FaRLiP.

Rather, the structural differences observed between *Calothrix* and *C. thermalis* suggest that, while the principal features of FaRLiP are conserved, there is scope for variation/tuning across species presumably in response to certain selection pressures (at present unknown). These variations therefore expand the current understanding of FaRLiP by showing a degree of evolutionary flexibility within the shared photoacclimation framework.

2. Could the authors provide additional information regarding their rationale for the selection of C. thermalis and Calothrix species as subjects for their studies. Additionally, was the presence of a monomeric PSII a priori apparent in Calothrix, or did it arise during a particular cultivation or purification technique?

C. thermalis was used because it is a well-established, arguably the best, model organism for Chl *f* based far-red photosynthesis. In our (biased) view, this status was established by the major study by Nurnberg et al 2018 (10.1126/science.aar8313), which reported the first isolation of FR-Photosystems, the first detailed spectroscopic and biophysical study of FR-photosystems, the first pigment quantifications, the first suggestion and evidence for long-wavelength photochemistry in both FR-photosystems, and the first sequence based models and pigment assignments. This work was followed up in several labs in the intervening years and has been the subject of several publications. To answer the reviewer's question, this species was originally chosen for that first study because it was available in culture collections and it grew pseudo-planktonically. It is obviously a great advantage that C. thermalis has been cultivated in our laboratory for many years, and robust growth and purification protocols were already established. It seems clear that determining the structure of

FR-PSII from the best studied species provides an important structural framework for interpreting the existing functional data.

Calothrix was selected at a later stage based on sequence comparisons suggesting differences in chlorophyll *f* binding sites relative to *C. thermalis*. Practical considerations also contributed, as the strain was available from a culture collection and turned out to be experimentally tractable (despite being filamentous). The purification procedures optimized for *C. thermalis* were adapted for *Calothrix*.

The presence of monomeric FR-PSII in *Calothrix* was not anticipated. We consider it likely that FR-PSII exists predominantly as a dimer *in vivo*, as observed for cyanobacterial PSII more generally. The monomeric state observed here most likely reflects increased fragility during isolation. We speculate that this may be related to the mesophilic nature of *Calothrix*, in contrast to the more thermotolerant *C. thermalis*, leading to reduced stability of the dimer under purification conditions. We hope to obtain experimental data relevant to the dimeric form of Calothrix PSII in our future work.

3. The manuscript identifies PsbH2' as a PSII subunit specific to far-red light. Could the authors tell us if PsbH2' has been identified in isolation from PsbH2 in any of the FaRLiP cyanobacteria? Or is PsbH2' always associated with PsbH2 (including fusion proteins)?

As stated in the text, based on our phylogenetic analysis, which includes most of the currently available far-red light (FaRLiP) sequences, as well as a previous study proposing PsbH2′ as a far-red–specific PSII subunit (10.1016/j.cub.2025.04.038), the presence of PsbH2′ is consistently linked to PsbH2 when the two are encoded as separate proteins. As far as we are aware, there are no FaRLiP strains in which PsbH2′ is present in the absence of PsbH2. In cases where they are not expressed as separate subunits, they occur as a fusion protein. Thus, PsbH2′ does not appear to exist independently of PsbH2 within the currently known diversity of FaRLiP cyanobacteria.

4. The HPLC analysis (Fig. S5) shows that the pigment compositions (Chl a/d/f) are highly consistent with previous reports. However, could you please provide additional information on any biochemical/proteomic techniques you may have used to verify the far-red PSII subunit composition at the protein level (e.g., mass spectrometry, immunoblotting)? Or was the identity of these subunits inferred largely from their genomic context and/or their densities in cryo-EM images?

Previous studies have reported mass-spectrometry characterization of isolated far-red (FR) photosystems. In the present work, we did not perform mass-spec analysis, as suitable samples were not available by the end of the study. Immunoblotting was also not performed, as reliable antibodies specific to far-red paralogues are currently unavailable. Instead, we assessed the integrity and composition of the complexes using native PAGE analysis of freshly solubilized thylakoids and purified samples prior to grid preparation. The migration patterns were consistent with those expected for far-red–adapted PSII and comparable to preparations routinely obtained in our group.

Subunit assignment in the final model was based on both the genomic context and the high-resolution cryo-EM density maps. The FaRLiP gene cluster contains single-copy far-red–specific paralogues, simplifying sequence attribution. Furthermore, the high resolution of our cryo-EM maps allowed confident sequence assignment using ModelAngelo (10.1038/s41586-024-07215-4), which confirmed the sequences in full. Together, the genomic context, map quality, and automated model building provide good confidence in the identity of the subunits incorporated into the final structure.

5. I would like to request a table that clearly outlines which subunits of PSII in each reviewed organism exhibit a far-red change or modification, as well as which core PSII subunits are conserved

with respect to white-light PSII in these studied organisms, so as to clarify the findings of the authors in a more conclusive manner.

This information is present in the Introduction (pp. 2, lines 11-20). Nonetheless, we agree that presenting it in a table would make it more immediately accessible. We have now included a supplementary table (Table S3) listing the subunits present in both structures. The table also indicates which subunits have far-red–specific paralogues and which are conserved with respect to white-light PSII, providing a clearer overview of the differences and similarities across the studied organisms.

6. Fig. 3A and 3B illustrate the application of Gaussian deconvolution to the absorbance spectra, with the fitted residuals shown; however, there is very little detail added to the methods section about the fitting procedures or the validations carried out to assess the methods. Could the authors provide more detailed information on the software used for model fitting, the fitted constraints, and how 'goodness-of-fit' was determined?

We thank the reviewer for highlighting this point and we agree that in the original submission, the details of the fitting procedure were not sufficiently described. We have now added a dedicated Methods section entitled "Constrained Gaussian deconvolution of FR-PSII low-temperature absorption spectra," which provides a detailed description of the fitting procedure, including the software used, applied constraints, and the criteria used to evaluate the fit.
In addition, the custom Python script used for the analysis has now been deposited and is available at: https://github.com/giovanniconsoli/77k-spectra-Gaussian-fitting.

7. The study describes the radiation-induced damage done, including the breakdown of disulfide bonds in PsbO (Fig. S4C) and changes in OEC interatomic distances (Fig. S4A/B), but does not include a discussion of the effects of these radiolytic degradation products on assignments of Chl f using ESP analysis.

These aspects have been discussed extensively in previous studies (10.1002/pro.3093; 10.1016/j.sbi.2019.04.006), and specifically for chlorophyll f (10.1126/science.ado6830; 10.1016/j.bbadva.2021.100019). These papers describe in detail how electron radiation damage affects electronegative moieties and why detection of Chl f formyl groups can be hindered under cryo-EM conditions. Nonetheless, the present work does not focus on the effect of the electron damage on the Chl f formyl groups, but on their assignment to specific structural sites. The apparent reduction of the Mn ions in the OEC etc does not influence the assignments of Chl f.

In this context, the discussion of disulfide bond reduction and Mn ion reduction is included in the supplementary materials to flag these potential radiation-induced effects to researchers interested in using the new structures for further analyses. Such effects have been described previously (10.1038/s42003-021-01919-3) and we consider the situation is similar in the present work. Our intention is therefore not to analyse electron-radiation associated damage per se, but to point out which structural features may be influenced by beam effects and thus should not be overinterpreted mechanistically.

I would suggest that the authors provide clarifications on a few points:

(a) Are there any limitations relating to beam effects on chlorophyll formyl groups?

Electron-beam damage to electronegative moieties is well documented. The C2 formyl group of Chl f is sensitive to accumulated dose, and attenuation of the corresponding signal can occur in later movie frames. However, modern dose-fractionation and frame-weighting approaches substantially

limit this effect in the final reconstructions (10.1126/science.ado6830; 10.1016/j.bbadva.2021.100019). Discussions of the effects of electron beam on chlorophylls f has been included in supplemental text 3.

(b) Are the Chl f ESP signals stable at the applied doses?

At the total dose used in this study, and following motion correction and dose-weighting, the ESP of Chl f formyl group features remain detectable and quantifiable with the cone scan method. This has been shown in FR-PSI already (10.1126/science.ado6830) for similar resolutions. Importantly, all Chl f molecules identified in this structure are hydrogen bonded at the C2 formyl position. The presence of a hydrogen bond partner makes the formyl group less electronegative increasing its signal in the map, as well as stabilizing the conformation and improving interpretability of the corresponding density.

(c) Can you provide a dose-fractionation analysis or add a discussion?

A systematic dose-dependent analysis of Chl *f* formyl-group behaviour is currently underway in our laboratory and we hope to publish it in future work. While such an analysis would be valuable, it lies beyond the scope of the present study, which focuses on structural assignment and comparative analysis of FR-PSII.

More generally, cryo-EM maps represent Coulomb potential rather than pure electron density. Because the Coulomb potential depends linearly on charge, negatively polarized atoms contribute less strongly to the reconstructed signal. As a result, highly electronegative substituents such as formyl oxygens are intrinsically more challenging to visualize, even at high resolution. In addition, when not stabilized by hydrogen bonding, conformational flexibility can further dilute the signal. These physical and methodological considerations have been described in detail in the references cited above. To avoid redundancy with prior publications, we will add a concise clarifying paragraph and explicit references in Supplementary text 3 to guide readers to the relevant literature and contextualize these limitations.

8. Fig. 3F and Supp. Text S3 reference papers/GitHub for the 4σ Z-score cutoff on Chl C2 classifications (Chl f vs. a) do not provide a rigorous empirical basis for using 4σ as opposed to other alternative cutoff values (i.e., Chl C2 = 3σ or Chl C2 = 3.5σ), nor do they provide any form of sensitivity/specificity on the results of using this cutoff and without confidence thresholds, nor do they confirm the use of known Chl a locations as being used for confirming corrected Chl a classifications for the data generated during the development of this work. Please provide additional information or cite another document in your response.

We agree that our manuscript did not provide sufficient information for a rigorous justification for the 4σ cutoff. A more formal framework for selecting an appropriate cutoff, together with the dose-fractionation analysis, is currently under development and will be presented in a future manuscript. In prior work the standard cutoff was 3σ . In the present study, we chose a slightly higher cutoff for the formyl groups to provide stronger confidence in interpreting our experimental data. The "improved cone scan" approach has been described in detail in Consoli et al., 2025 (10.1126/science.ado6830); for greater accessibility, we have now included the key principles of this analysis in the Supplementary text 3.

To address the reviewer's concerns further, we now provide some of our ongoing advances in chlorophyll substituent analysis (Supplementary text S3). For each map/PDB model combination, confusion matrices were generated for a Z-score cutoff range from 1 to 10, and precision, recall, and accuracy metrics were calculated. The Z-score cutoff at which accuracy is maximized provides

guidance for selecting an appropriate threshold and helps evaluate the significance of a given substituent relative to a methyl distribution. This analysis clarifies the rationale behind the chosen 4σ cutoff and demonstrates the robustness of the classification approach.

9. Differential Chl f content was identified using HPLC (Fig. S5), further supporting the proposed structures.
However, the methods did not provide the following:
(A complete record of this information is essential to ensure reproducibility of the results.)

(a) retention time of the Chl a/d/f plus standard compounds;
The retention times of Chl *a*, *d*, and *f* are now indicated in the figure and the legend of figure S5. The HPLC run was not spiked with a commercial standard, but rather the characteristic absorption spectra of each peak and their relative elution times were used as internal standards to confirm their identities. While the additional internal standard would have been necessary for absolute quantification, the objective here was to determine the relative amount of each chlorophyll present, for which the presence of an internal standard is not necessary.

(b) evidence that the retention of Chl d (696nm) and Chl f (707nm) was fully resolved;
The elution peaks of Chl *a*, *f*, and *d* are temporally separate, as shown in fig. S5. WE have now provided additional absorption spectra for each peak (fig. S5 panel D,E,F) also confirm the identity and purity (i.e., full resolution) of each peak.

(c) use of peak integration/baseline integration to quantify the concentrations;
We thank the reviewer for the suggestion. The method section has now been updated to describe the method of quantification.

(d) provides replicates (n=?) for all measurements and uncertainty (mean ± std);
Due to the scarcity of these samples, the HPLC analysis was performed only once. While we recognize the importance of replicates, the pigment analysis in this work is not central to the main conclusions but serves only as a supplementary line of evidence. The pigment count using HPLC verified that *Calothrix* far-red PSII lacks two of the four Chl *f* molecules present in other FR-PSII. This was also demonstrated by i) the structural work, ii) the amino acid alignment and iii) the absorption spectra, all of which are shown in the present work, so we consider that the HPLC is not needed for the conclusions reached in this paper. The text has been updated to clarify this point. Additional replicates will be performed in future studies. If this is seen as a problem this data can be removed without affecting our main conclusions.

(e) co-elution samples for Chl d and Chl f.
The elution of Chl *d* and Chl *f* in the described HPLC protocol is completely temporally separate, as demonstrated in the updated Figure S5. The lack of commercially available standards or methods to isolate these chlorophylls prevents direct co-elution testing. However, we have also performed HPLC runs using *Acaroychloris* chlorophyll extract (99% chlorophyll d, 1% chlorophyll a) as a replacement for the commercially unavailable pure chlorophyll d. This confirmed the elution time of chlorophyll d. The apparent co-elution of chlorophyll d and f in the chromatogram in Fig. S5 (i.e. 707 nm absorbance in the chlorophyll d peak, and 696 nm absorbance in the chlorophyll f peak) is due to the absorption spectra of each specific chlorophyll (the tail of the Qy peak of chlorophyll d goes into 707 nm, and vice versa for chlorophyll f). The absorption spectra of each peak confirms that chlorophyll a,d and f elute separately and there is no co-elution.

10. Your wavelengths have been assigned based solely on your comparison between C. thermalis (dimeric with four Chl f (completely extrinsic subunits)) and Calothrix sp. (monomeric with two Chl f (with PsbO, PsbU, and PsbV absent)).

For this comparison to be valid, all of the following must be addressed:

(a) Were the proteins PsbO, PsbU, and PsbV not present in the native Calothrix FR-PSII, or did they get removed during purification with 0.4% β-DM?

PsbO, PsbU, and PsbV are all present in the Calothrix genome (table S3) and are assumed to be part of the functioning PSII complex *in vivo*. These extrinsic subunits were likely lost during the solubilization step at 0.4% β-DM, or the following purification. In the cryo-EM dataset, a substack of particles exhibits ESP density corresponding to the extrinsic proteins, but the number of such particles is insufficient to produce a high-resolution reconstruction.

(b) When you removed the extrinsic subunits, did it not affect the water oxidation complex structure, the coordination of the Chl pigments, or any water networks?

The extrinsic subunits were lost during the purification procedure. This is often the case in PSII core preps from mesophyllic cyanobacteria (e.g. Synechocystis sp. PCC 6803 and FR Synechococcus sp. PCC 7335) and from plants. The absence of extrinsic subunits compromises the donor side and results in the loss water oxidation activity as seen by the lack of oxygen evolution (data not presented). The channels for incoming substrate (H2O) and outgoing products (H^+ and O_2) are also perturbed or lost. However, the locations, coordination and environments of all chlorophyll molecules remain unchanged. The FR-PSII structure from Calothrix retains all expected chlorophylls in identical positions, indicating that the pigment site energies are not perturbed by the loss of the extrinsic proteins. We know from years of experiments on *C. thermalis* FR-PSII (Nürnberg et al 2018) that variations in the intactness of the donor side (extrinsic polypeptides and Mn complex) have no noticeable effect on low temperature absorption and fluorescence spectra. Any variation in our spectra has come from different degrees of contamination from the FR-allophycocyanin (see the SI in Nürnberg et al 2018) and in rare cases, from FR-PS1 contamination. Furthermore, low-temperature absorption spectra previously reported in the literature are consistent across FR-PSII preparations (even from different organisms), regardless of the presence or absence of extrinsic subunits.

(c) What are the reasons that you can conclude that the loss of the peaks at 721 and 749 nm is due to the absence of Chl f and not to structural changes caused by purification?

The loss of extrinsic proteins and most of the Mn are not expected to influence the site energy of the chlorophyll pigments, (see answer to 10B above) as not significant structural perturbations to the chlorophyll binding sites. The RMSD of the two complexes isolated is low (0.484 Å), and no changes are evident that might result in a site energy shift in proximity of any of the chlorophyll pigments. In both structures, a chlorophyll molecule is present in both the B608 and B617 sites, indicating that the loss of absorbance at 721 nm and 749 nm is related to the presence of Chl a in these sites in Calothrix while Chl-f molecules are present in these sites in *C. thermalis* PSII. It is clear that the lack of chlorophyll f in these positions in Calothrix cannot be attributed to the preparation nor is it associated with the loss of the Mn or extrinsic polypeptides.

(d) If you compare a completely dimeric structure (C. thermalis) to an incompletely monomeric

structure (Calothrix), should Calothrix also possess complete extrinsic proteins to allow a valid comparison?

The question is answered in above for 10 b and c. For the comparisons made (absorption spectra), the comparison is valid based on earlier spectroscopy and supported now by well resolved structural data. All chlorophylls are present and occupy comparable chemical environments.
Ideally, comparisons would be between complexes of identical intactness, we have not yet been able to purify fully intact FR-PSII from *Calothrix (*nor for *C thermalis* FR-PSII though we have made much progress) and consequently, comparisons of functional metrics (e.g., quantum efficiency, O_2 evolution, charge recombination, photodamage), cannot yet be made. We are confident we can improve the preps. But for now the absorption spectra are not compromised, and they provide remarkable new insights that allow the 4 long wavelength antenna chlorophylls to be both localised and their wavelengths assigned. This is a remarkable step forward in the field.

(e) Have you looked at the absorption spectra of intact Calothrix cells or thylakoids derived from Calothrix sp. NIES-3974 to determine if your purified Calothrix structure resembles that of the native Calothrix?

Direct measurement of intact cells or thylakoids is complicated by overlapping long-wavelength absorption peaks originating from FR-PSII and FR-PSI (8 Chl *f* molecules absorbing between 725 nm and 800 nm), as well as contamination from FR-APC (with absorption peaking at 713 nm) making deconvolution impractical if not impossible.

To verify the spectral properties of isolated FR-PSII in comparison with *in vivo* FR-PSII, low-temperature fluorescence emission spectra were measured in whole cells of both strains. These data reproduce the behaviour observed in the isolated complexes (Fig. 3C), confirming that those changes are not related to changes occurring during the isolation procedure.

We conclude that, considering the evidence available, the degree of intactness of the *Calothrix* FR-PSII samples presented in this work is appropriate to draw the conclusions regarding Chl f location and color assignments.

Minor issues.

1. Could you please add the scale bar to Fig. 1 and the panels in the proper way?
Structural snapshots in Fig. 1 were acquired in “mono” perspective in chimerax (apparent size decreasing with distance from the viewer). Therefore, the scalebar would not be meaningful.
2. Fig. 1 D – the authors use the term “Stromal”. Perhaps it could be a bit more accurate to use the term “cytoplasmic” for cyanobacteria.
We agree with the reviewer the term stromal is inappropriate, it has been changed to “cytoplasmic”
3. Fig. 3 in the title, a small typo - “assignement”. Please change to “assignment”
Fixed
4. Fig. S9 – In the figure description, we see “FRL-PSII”. Is it the same as FR-PSII used in the main manuscript? Please correct or define.
Fixed. Sorry about the mixed abbreviations.

5. Grid preparation – the authors mentioned “Gold quantifoil 2 µm hole size and 1 µm hole spacing” Did they mean Quantifoil Au R2/1, 300 mesh?

Fixed.

6. The microscope used in the acknowledgement part is specified as Krios G3i, but in the Data collection table, as Krios III. Please use consistent nomenclature throughout.
The nomenclature was unified.

7. Please check the particle number consistency throughout the text and tables again.
We apologise for the inconsistency and have updated the text accordingly.

In summary, this study offers new structural information for a far-red light-responsive form of PSII with atomic resolution and provides valuable information on how organisms can adapt their photosynthetic function (far-red adaptation) under changing conditions (far-red light intensity), even if certain aspects of this study require additional explanation and clarification with respect to methodological aspects.
Therefore, I recommend this manuscript for publication in Nature Communications upon satisfactory revisions.

Reviewer #3 (Remarks to the Author):

The manuscript by Leong and coworkers describes new results regarding the molecular layout and specific properties of photosystem II (PSII) from far-red adapted organisms. The authors combine structural insights based on cryo electron microscopic studies and results from spectroscopic and phylogenetic analysis to describe the energy transfer network used in the red light adapted version of PSII. The authors describe the structure of far red PSII from C. thermalis 7203 at 2.17 Å and from Calothrix 3974 at 2.33 Å resolution using single particle cryo electron microscopy.

In the Thermalis structure the position of 4 Chl f in the antenna system of the complex was described, 3 of them matching with previous reports from structures of FR adapted PSII from other organisms and one assigned differently from previous work. In addition, the presence of a new subunit, PsbH2', that has not been described before is described. In contrast, the Calothrix structure does not have PsbH2' and only shows two Chl f molecules in the antenna system. By directly comparing spectroscopic data for both together with the structural information Leong et al are able to specifically assign the different far red absorption bands in both complexes to specific Chl f molecules. In addition the authors suggest a model of the cofactor arrangement in a far red adapted PSII allophycocyanin super-complex and depicted energy transfer pathways from the phycobilin external antenna towards the reaction center involving specific Chl f molecules that show red shifted spectral properties. Several recent studies have also targeted similar far-red acclimated versions of PSII and this is a very active field of investigation in the photosynthesis research community, trying to decipher the changes in the entire photosynthetic apparatus, including PSII, Photosystem I and the antenna complexes upon shift from normal to far red light conditions.

Assignment of Chl f:

1) A previous study by Gisriel and colleagues already provided assignment of four Chl f in the far red light adapted PS II complex from Synechococcus 7335. While the authors of the current manuscript state that the assignment in that work (ref. 4, Gisriel et al, 2023) was not conclusive based on electrostatic potential maps for all four Chl f, the Gisriel paper actually shows ESP scans for all four Chl f and provides assignment of these based on the scans. The local resolution for Chl f 507 in Gisriel

et al 2023 was reported to be only 2.74 Å and hence the ESP scans are less conclusive compared to the high resolution data presented in the current manuscript. But the authors here should maybe more clearly state that previous work also used ESP scans for assignment of four Chl f. Also it would be nice to report the local resolution of the current data for each of the Chl f that are discussed in the manuscript so that it is easier for the reader to appreciate the local data quality.

We thank the reviewer for this suggestion; the original text stated: "However, the improved resolution allowed only some Chl f molecules to be assigned by analysis of the electrostatic potential (ESP)." We agree that the work by Gisriel et al. presented ESP scans for all four assigned Chl f molecules and used these analyses to support their assignments. Our original wording did not sufficiently reflect this, and we have revised the manuscript to clarify that ESP scans were indeed employed in that study.

Since Gisriel's publication, we have further refined the cone-scan methodology used for ESP analysis (Consoli et al 2025). While the conceptually is basically similar, the updated approach applied here evaluates a broader sampling region and incorporates a modified outlier-detection strategy with a more robust statistical framework. These methodological differences are now clarified in the revised manuscript. In addition, as suggested, we now report the local resolution values for each Chl f discussed. The legend of fig. S14 (fig. S15 in previous version) has been updated to include the local resolution at the Mg^{2+} position of each chlorophyll, allowing readers to directly assess the data quality underlying the assignments.

2) The authors describe the possible location of a water next to Ser B107 in the Synechococcus structure by Gisriel et al as an indication that the Chl B617 could also be a Chl f in Synechococcus. Does this imply that the authors conclude that the Chl f assignment by Gisriel and colleagues in their Synechococcus RL structure is not correct? Do you suggest that instead one of the other Chl's assinged to Chl f by Gisriel et al should be instead assigned as Chl a to keep the number of Chl f at 4 in both the Synechococcus and the Thermalis structure? If I understand it correctly the authors suggest that Chl B605 is not a Chl f in Synechoccus. But is surprising that Chl B605 in the Synechococcus structure showed the clearest result of the cone scanning procedure and has also the highest local resolution reported.

We acknowledge that the cone scan analysis by Gisriel et al. identified B605 as a statistical outlier, whereas B617 was not detected as such. However, as noted by the reviewer, we believe that in Synechococcus, B605 may have been incorrectly assigned as Chl f, and that B617 may not have been detected due to its comparatively lower local resolution.

We have performed our improved cone scan method (Consoli et al. 2025) on 8EQM. Inspection of the raw ESP patterns from the C2 substituent of B605 (figure below) shows that the *Synechococcus* and *C. thermalis* are qualitatively similar in features but differ markedly in signal intensity and centring of the density. The relative increase in ESP observed around the C2 substituent of B605 in the *Synechococcus* structure could plausibly arise from suboptimal model fitting or sharpening artefacts. It is not clearly stated whether sharpened or unsharpened maps were used in the original analysis, and such processing differences can significantly influence local ESP features.
Furthermore, B605 exhibits the least convincing set of conserved phylogenetic changes among the sites proposed as Chl *f*. In particular, the proposed hydrogen bond between the formyl group of Chl *f* B605 and the phenylalanine ring hydrogen appears chemically unlikely.
It also remains possible that the difference in Chl *f* sites is due to species differences (akin to *C. thermalis* vs. *Calothrix* sp.) and therefore, in absence of more conclusive evidence on Synechococcus. we have decided not to insist on this aspect in our manuscript.
Nonetheless, whether the assignment of B605 instead of B617 as a Chl *f* is correct in Synechococcus does not affect the conclusions of this manuscript, as either the absence of Chl *f* B617 (as per Gisriel

et al.) or the altered Chl *f* B617 site (modified hydrogen bonding network) in Synechococcus can both be interpreted functionally with the shift of fluorescence emission from 750 nm to 740 nm, indicating that B617 is the 749 nm pigment.

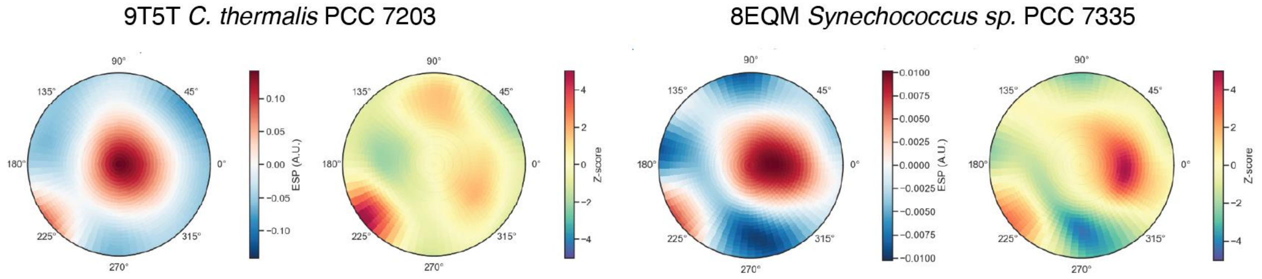

3) If at the same time this Chl was wrongly assigned as Chl f, as indicated in the current manuscript, then it becomes questionable if the cone scanning method is actually useable to decide about the nature of Chl in these ESP maps. The authors should comment on this.

We do share the reviewer's view on this point. We believe that quantitative methods in cryo-EM, especially those that rely on the fitted model, such as the cone scan, should not be used prescriptively, but rather as tools to identify statistical outliers in the strict statistical sense of the term. Differences in ESP can arise from multiple factors, not solely from the presence of an additional formyl oxygen. Therefore, the overall shape of the ESP density, as well as the general quality of the map and the fitted model, should be considered when drawing conclusions about whether a substituent corresponds to Chl f.

This consideration has motivated our efforts in previous work developing the cone scan method (Consoli et al. 2025). In particular, we aimed to provide a more solid statistical framework and a more explicit visual/spatial analysis to complement the conceptual approach originally introduced by Gisriel et al. In the latest iterations of our methodology, a more formal framework for selecting an appropriate Z-score cutoff and for assessing the quality of the map/model combination has been implemented. This was not discussed in the supplementary section of this paper, but the reviewer's opinions convinced us that this has to be more clearly stated and was added in the methods section and in the initial section of supplementary text S3 but is reported here for simplicity.

The standard cutoff adopted by Gisriel et al. for outlier detection was 3σ. In the present study, to assess the quality of the analysis for a given map and PDB model combination and to determine an appropriate standard deviation cutoff for classifying a substituent as significantly different from a methyl distribution, confusion matrices were calculated across a Z-score classification range from 1 to 10. Precision, recall, and accuracy metrics were computed for each threshold. The Z-score cutoff at which accuracy is maximised for a given map/model combination should be the one used as an indication of the most appropriate threshold and helps in interpreting the nature of individual substituents.

To compute these metrics, moieties common to different chlorophyll types were used as internal references. In both chlorophyll a and chlorophyll f, C3 and C8 are non-methyl substituents and are therefore expected to show variation in their ESP relative to a methyl distribution. In contrast, C7 and C12 are methyl substituents and are therefore not expected to show variation relative to the methyl distribution.

Within this framework, true positives (TP) are defined as non-methyl substituents that present ESP values outside the methyl normal distribution at a given Z-score threshold. False positives (FP) are methyl substituents that present ESP values outside the methyl normal distribution. True negatives (TN) are methyl substituents that do not present ESP values outside the methyl normal distribution. False negatives (FN) are non-methyl substituents that do not present ESP values outside the methyl normal distribution.

Compared with our present map/model combinations, the deposited map/model pair from Gisriel et al. (8EQM) yields substantially lower precision, recall, and accuracy values, indicating reduced confidence in outlier detection for that dataset (figure below). Moreover, irregular trends in the accuracy metrics suggest that factors other than atomic ESP are likely contributing to the final classification results.

[REDACTED]

In conclusion, assignments should not be made solely on the basis of cone scan ESP analysis, but must be supported by additional lines of evidence, particularly conserved amino acid changes around key chlorophyll substituents (e.g., C2 and C13 hydrogen bonds) and changes in Mg-coordinating residues. All assignments presented here are further supported by comparison between the two species: the conserved changes around B614 and C507, as well as the loss or reversion of amino acid substitutions around B608 and B617 to the white-light chlorophyll a configuration, fully correspond with the observed differences in pigment composition and low-temperature absorption spectra. These independent lines of evidence strongly support the assignments proposed in this work.

4) The fig. 2 showing the location of a Chl f at position B617 needs to be improved. The figure or the figure legend should state that you are actually showing data for C. thermalis here. The enlarged view shown in panel C should actually be connected with panel B and not with panel A as the view direction is similar in B and C (maybe you could swap orientation in A and B?). Panels D and E are not clear for non-expert readers. I suggest if space permits to include parts of figure S13 into figure 2 to illustrate the density scanning approach that is utilized in the present manuscript to decide about Chl identity. Accordingly it would be good to include some text in the main manuscript explaining the density scanning and the resulting polar and Z score plots. Also it might be nice to show the structures of Chl a, Chl d and Chl f in either the main text figures or in the SI as many non-expert readers might not know the subtle structural differences between them.

We thank the reviewer for these constructive suggestions. Fig. 2 has been revised accordingly. The figure legend now explicitly states that the structural data shown correspond to *C. thermalis*. Panels A and B have been swapped to ensure that the orientation in panel C aligns with panel B, improving visual continuity between the overview and enlarged view. To improve clarity of panels D and E for

non-expert readers, additional visual guides have been incorporated to facilitate interpretation of the cone scan representation. Furthermore, elements of former Figure S13 illustrating the density scanning approach have been integrated into Fig. 2 to better contextualize the methodology used to determine chlorophyll identity. A brief explanatory paragraph describing the ESP cone scan method, including interpretation of the polar plots and Z-score analysis, has been added to the main text (page 5, line 2-11). In addition, a figure depicting the structural differences between chlorophyll a, d, and f has been included in the Supplementary Information to aid readers unfamiliar with their distinguishing features.

5) In figure 3 E the monomeric structure of Calothrix 3974 PSII is shown but the legend says dimeric. Please correct.

The text has been corrected.

6) LHG416 (fig. 1) is not modelled well into the map, not sure if it can be clearly identified based on the map (see below). Please check and comment on this.

We thank the reviewer for drawing attention to this point. We agree that the ESP density in this region does not allow an unambiguous assignment of the lipid headgroup. Indeed, the density does not perfectly match the headgroup geometry of any of the known lipid species typically found in cyanobacterial thylakoid membranes. It is possible that this lipid adopts more than one conformation and therefore shows a more ambiguous and apparently anomalous ESP around the head group.

To reflect this uncertainty more clearly, we have modified Fig. 1 to show the ESP density around this site and have revised the figure legend to describe the fitted lipid as "tentative." Our intention in modelling this lipid was to account for the observed density and maintain structural completeness, while explicitly acknowledging the lower level of confidence in its specific identity.

7 The additional PsbH2' subunit: The reported identification of the additional subunit PsbH2' is very interesting. It seems to modulate the monomer-monomer interface and while no direct contacts are formed between PsbH2' with residues from the other monomer in a dimer its presence clearly reduces the volume in the monomer-monomer interface accessible to lipids. Are there any indications that the presence of PsbH2' influences the dimer stability? Could the authors compare the yields of dimeric and monomeric PSII obtained from samples under FR and WL conditions for C. thermalis?

Currently we have no indications that PsbH2' influences dimer stability, as this is the first reported instance where PsbH2'-contaning PSII was isolated. Although interesting, its influence on PSII assembly remains outside the scope of this study, as it requires mutagenesis work for a sufficiently rigorous experiment (which is also currently underway).

8 Connection to the FR-APC:
It would be nice to actually show where PsbH and PsbH' is located in the APC-PSII complex to allow the show where PsbH and PsbH' is located in the APC-PSII complex to allow the reader to better follow the argument about the importance of PsbH' for better interaction with the FR form of APC. One caveat in the modeling is that the overall phycobilisome-PSII structure from a white light acclimated sample was used to fit the FR-APC and the FR-PSII together. It should be stated that there might be differences in the overall assembly upon FR acclimation that are not captured by the white light supercomplex.

We thank the reviewer for the suggestions, and we have added an additional figure in the SI to show the relative positions of PsbH2 and PsbH2' and the APC (fig S17).

9 The distances between ApcE2 and Chl f in the complex are discussed but it would be good to also describe/show the distance of terminal emitters from the APC to any other Chl a. Are the Chl f at a shorter distance compared to the closest Chl a?

We thank the reviewer for this suggestion. The text has been updated to indicate that several Chl a lie at similar distances from the ApcE2 bilin as the Chl f pigments (CP43 side: ~23 Å to C513, ~24 Å to C511, ~29 Å to C512; intermonomer side: ~29 Å to B610).

Nonetheless, experimental data show that far-red absorbing APC (FR-APC) can efficiently transfer excitation energy to Chl f molecules in vivo (https://doi.org/10.1038/s41467-022-31099-5; https://doi.org/10.1038/s42003-025-08326-y). These observations indicate that a direct connection from the bilin in ApcE2 to Chl f in FR-PSII is both feasible and functionally relevant, even in the presence of similarly distant Chl a molecules (see further discussion below).

10 Also given the very long distance of >27 A exciton transfer is likely slow from ApcE2 to any of the Chl f. Would it be faster to a Chl a? Maybe good to discuss this. Are there any experimental data for the energy transfer times from APC to the core in white light versus far red light adapted complexes? This would be good to discuss here.

While the center-to-center distance between ApcE2 and the nearest Chl f exceeds 27 Å, excitation energy transfer rates are determined not only by distance but also by spectral overlap, orientation factors, and energetic alignment.

Yes, that data is presented mainly in a work from Mascoli et al. (https://doi.org/10.1038/s41467-022-31099-5). In this time-resolved spectroscopic study, the authors have shown that charge separation is faster in FR-PSII in the presence of FR-APC. This acceleration indicates efficient energy transfer from far-red bilins to the red-shifted chlorophyll network and ultimately to the reaction center Chl d. If excitation routes involved preferentially the uphill transfer event to Chl a prior to reaching Chl f, the presence of the far-red antenna would not be expected to accelerate trapping.

The experimentally observed faster trapping in FR-PSII therefore supports a functional energy-transfer pathway involving Chl f rather than a Chl a mediated route. Since the transfer event from Chl a to Chl f is definitely slower than the one from the Bilin in ApcE2 to the network of chl f molecules in FR-PSII.

Therefore, excitation energy transfer from the FR-APC to the FR-PSII must be dominated by direct transfer between the bilin in ApcE2 and C507. Nonetheless, it is not possible to exclude that a minor portion of the excitation energy is first transferred uphill to chlorophyll *a* and then rapidly transferred to the low energy sink that is the chlorophyll *f* molecules. However, the emission of FR-APC at ~730 nm means that there is a ~50 nm energy gap between the FR-APC emitter and any chlorophyll *a* in the system, making the transfer likely slower than directly to chlorophyll *f*.

Comments on the SI:

i) Text of the SI in general should be checked carefully as there are many examples of bungled sentences or repetitions.
We have now improved the writing of the SI.

ii) Section in the SI about nature of RC chlorophyll could move to the main text as well as fig. S13. Without that figure the panels D and E in Fig. 2 make no sense to the reader.
The explanation of the cone scan ESP method has been added to the main text as well as guidance on how to interpret the cone scan plots in Fig.2. Therefore, removing the need to add fig. S13 to the main text.

iii) SI page 4, line 11: please include the reference for Modelangelo (Jamali e al, Nature 2024)
This has been rectified.

iv) Chl f B614: H-bond to B Trp462 would result in an angle of 86 deg between CMB, OMB and Trp NE in the thermalis structure, not a likely geometry for an H-bond.

A study surveying the equivalent H-bond angle in the PDB databank (Figure 4, bottom panel in 10.1073/pnas.0307578101) shows that although 120° is canonical and frequent, there are instances where this angle is closer to 80°. However, we agree with the reviewer that the CMB-OMB-HNE1 angle should have a theoretical optimum of 120°, and the 34° deviation from this optimum would weaken this interaction. Nonetheless, it can be appreciated in fig. S14 that the features present in the Z-score analysis of the ESP density of the C2 of B614 that the orientation of the density compared to the Chl ring plane is extremely similar between *C. thermalis* and *Calothrix*. To address this, we have added a sentence to the section discussing the chemical environment of Chl B614.
"The hydrogen-bond geometry between the CMB/OMB atoms and PsbB Trp^{462} in *C. thermalis* appears suboptimal, suggesting that this arrangement may reflect refinement limitations rather than a true structural feature. However, the cone-scan analysis reveals increased C2 ESP density oriented toward Trp^{462}, supporting the presence of a formyl group engaged in hydrogen bonding (or at least, favorable electrostatic interactions). Together, these observations are consistent with both formyl substituents interacting with PsbB Trp^{462}."
v) SI page 19 line 1-5 please rewrite this text as there are contradictory statements in these few sentences
We apologise for the confusion; the text has been rewritten and the contradictions removed.

vi) SI page 19 line 12,13: "Calothrix sp. retains the white light conserved residues and the white light subunit PsbH and presents a Chl a molecule in this site."
This statement is very confusing as I thought from fig. S9 B that a Chl f was modelled in this site for Calothrix sp. Please explain/correct the text accordingly.
We apologise for the typo and confusion; this sentence has been removed entirely.

SI page 20, line 5: " ligand to the chlorophyll where the backbone carbonyl of PsbC Ser266."
The sentence has been removed

Please fix this sentence: SI page 20, line 7:" The torsion angle of this residue is maintained by the presence of another conserved change, PsbC Gln267 (a Met in WL-PSII), that is likely to improve the stability of the site and possibly increase the electron withdrawing effect of PsbC Asn271 on the C2 formyl of Chl C507. "
This is not convincing. The two residues are about 9 A from each other and it is not clear how one could affect the torsion angle of the other over this distance. The other explanation for this change given further below is more convincing (changing site energy of the Chl a C505).
The sentence has been removed.

Comment on model quality:
The model provided does not seem to always be an optimum fit to the experimental ESP maps. Some examples are listed in the attached file and the authors should maybe reconsider parts of the modeling before final deposition.

We thank the reviewer for the careful evaluation of the deposited model. The highlighted regions were re-examined in detail, and the structure has been updated accordingly. The C-terminal region of chain R was rebuilt, and water molecules lacking clear supporting ESP density were removed. The model was subsequently re-refined in Phenix, and the updated coordinates have been redeposited.

Comment on model quality for Leong et al:

The model provided does not seem to always be an optimum fit to the experimental ESP maps. Some examples for the Calothrix 3974 model are listed below and the authors should maybe reconsider parts of the modeling before final deposition.

Loop region of subunit PsbO: Residues O80 to O90 are modelled badly

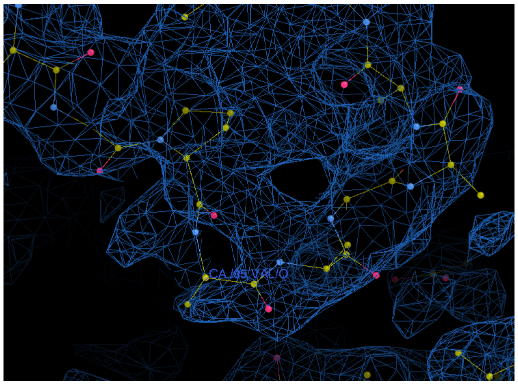

Lipid LHG416 (listed as LHG I101 in the pdb file): The map for 9T5T is not matching the modeled head group of this phospholipid. Maybe reconsider assignment of LHG to this density.

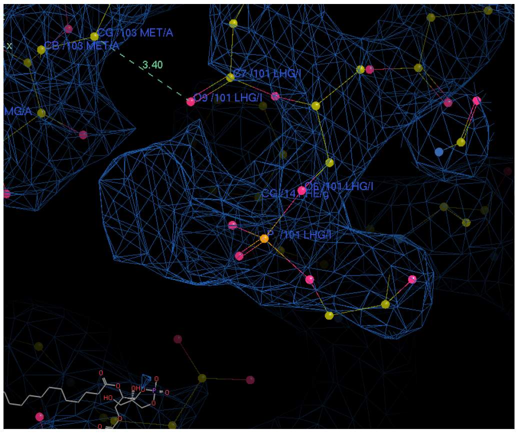

Chain R: The C-term of chain R is not matching with the map.

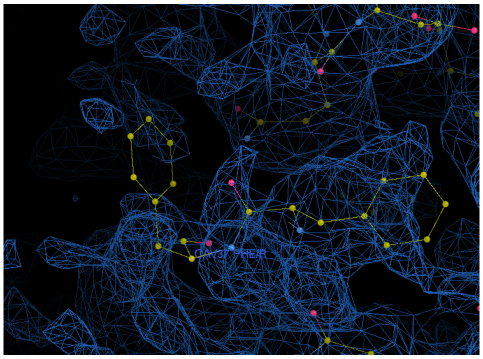

Waters: There are several instances where no density is visible for waters included in the model. In these cases it would be better to remove the waters from the model. One example are waters next to the OEC. Why are these even included in the model if there is no map visible at all? In other instances it seems like the modeled water positions are shifted from the position of peaks in the map. Please check carefully for such errors before final deposition.

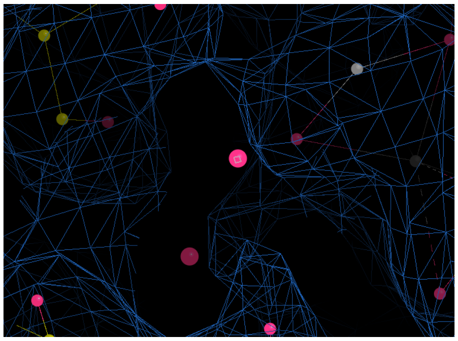

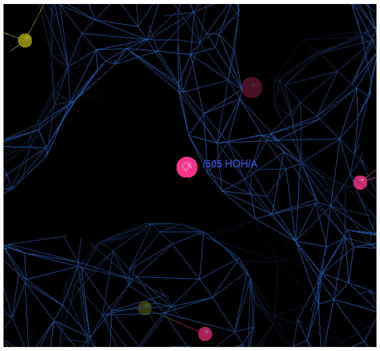

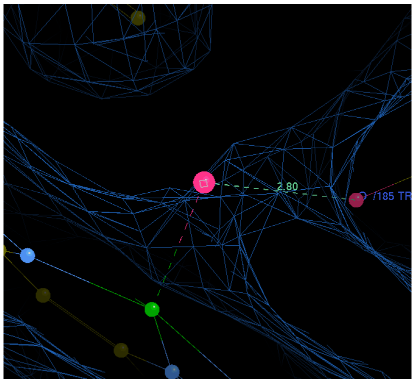

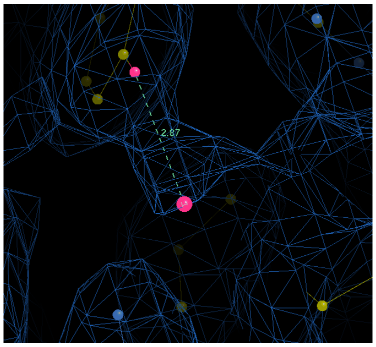

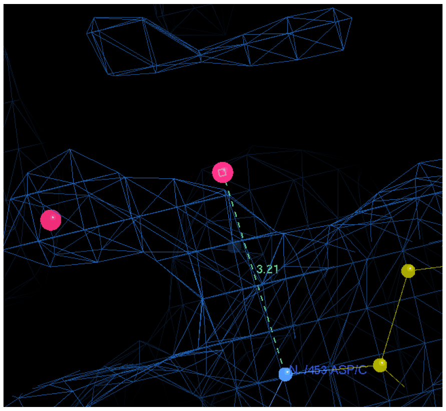

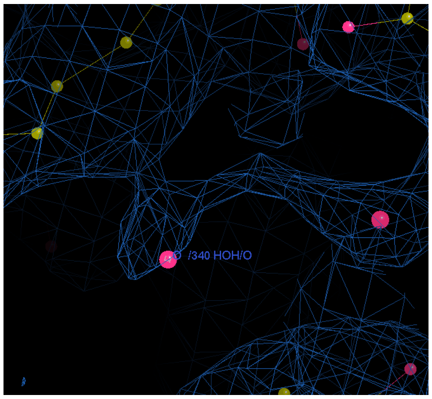

Supplement: Supplementary file 3 — Transparent Peer Review file [file 41467_2026_73964_MOESM3_ESM.pdf]
